# Dehydroabietic oximes halt pancreatic cancer cell growth in the G1 phase through induction of p27 and downregulation of cyclin D1

**DOI:** 10.1038/s41598-018-34131-1

**Published:** 2018-10-29

**Authors:** Laura E. Kolsi, Ana S. Leal, Jari Yli-Kauhaluoma, Karen T. Liby, Vânia M. Moreira

**Affiliations:** 1 Drug Research Program, Division of Pharmaceutical Chemistry and Technology, Faculty of Pharmacy, University of Helsinki, Viikinkaari 5 E, (P.O. Box 56), FI-00014 Helsinki, Finland; 2 Department of Pharmacology and Toxicology, Michigan State University, 1355 Bogue Street, East Lansing, MI 48824 USA; 3 Strathclyde Institute of Pharmacy and Biomedical Sciences, University of Strathclyde, 161 Cathedral Street, Glasgow, G4 0RE UK

## Abstract

Low 5-year survival rates, increasing incidence, as well as the specific challenges of targeting pancreatic cancer, clearly support an urgent need for new multifunctional drugs for the prevention and treatment of this fatal disease. Natural products, such as abietane-type diterpenoids, are widely studied as promiscuous anticancer agents. In this study, dehydroabietic oximes were identified as potential compounds to target pancreatic cancer and cancer-related inflammation. The compounds inhibited the growth of human pancreatic cancer Aspc-1 cells with IC_50_ values in the low micromolar range and showed anti-inflammatory activity, measured as the inhibition of nitric oxide production, an important inflammatory mediator in the tumour microenvironment. Further studies revealed that the compounds were able to induce cancer cell differentiation and concomitantly downregulate cyclin D1 expression with upregulation of p27 levels, consistent with cell cycle arrest at the G1 phase. Moreover, a kinase profiling study showed that one of the compounds has isoform-selective, however modest, inhibitory activity on RSK2, an AGC kinase that has been implicated in cellular invasion and metastasis.

## Introduction

Pancreatic cancer is the fourth leading cause of death by cancer in Europe and in the US^1^. In the US alone, it is estimated that over 55 000 new cases of pancreatic cancer will be diagnosed in 2018. The increasing incidence and death rates alarmingly suggest that it will become the second leading cause of cancer-related deaths before 2030^2,3^. The current treatment protocols for patients with pancreatic cancer include surgery, with partial or total removal of the pancreas, radiation therapy, and mixed-drug chemotherapy, depending on the type and stage of the diagnosed cancer. Nonetheless, the 5-year survival rates for this fatal disease still remain at 9% in the US and 3% in Europe, with most patients succumbing to the disease between 4.6 months and 2 years after diagnosis, clearly demonstrating the need to improve early diagnosis and to provide more effective and safer treatments. Pancreatic cancer is particularly hard to target because 67–100% of tumours contain on average 63 genetic mutations per cancer, involving the impressive number of 12 altered cellular signalling pathways and processes^4^. Therefore, developing multifunctional compounds able to reach several relevant drug targets, that modulate entire regulatory networks or multiple pathways, being both preventive and therapeutic, is much more likely to serve as an effective treatment for this devastating disease^5^,^6^.

Nature is an important source for finding new anticancer drugs. Between 1981 and 2014, 83% of all approved small molecule anticancer drugs were either natural products or their derivatives or natural product mimicks^7^. Terpenoids are a large group of phytochemicals that have been explored as potential cytoprotective and chemopreventive agents. According to several preclinical animal model studies, both naturally occurring and semi-synthetic terpenoids act at various stages of tumour development including inhibiting initiation and promotion of carcinogenesis, inducing tumour cell differentiation and apoptosis, and suppressing tumour angiogenesis^8–13^. For instance, two derivatives of the triterpenoid oleanolic acid, 2-cyano-3,12-dioxoolean-1,9-dien-28-oic acid (CDDO) and 2-cyano-3,12-dioxooleana-1,9(11)-dien-28-oic acid methyl ester (bardoxolone methyl or CDDO-Me), progressed into phase I clinical trials for the treatment of leukaemia as well as solid tumours and lymphoid malignancies^6,14,15^. Highly oxygenated abietane-type diterpenoids such as triptolide and minnelide, and tanshinone A, have been studied for the treatment of pancreatic cancer^16–20^. Minnelide is a prodrug of triptolide with improved solubility and is currently being tested in clinical trials for advanced tumours including pancreatic cancer^21,22^.

**Figure 1:**
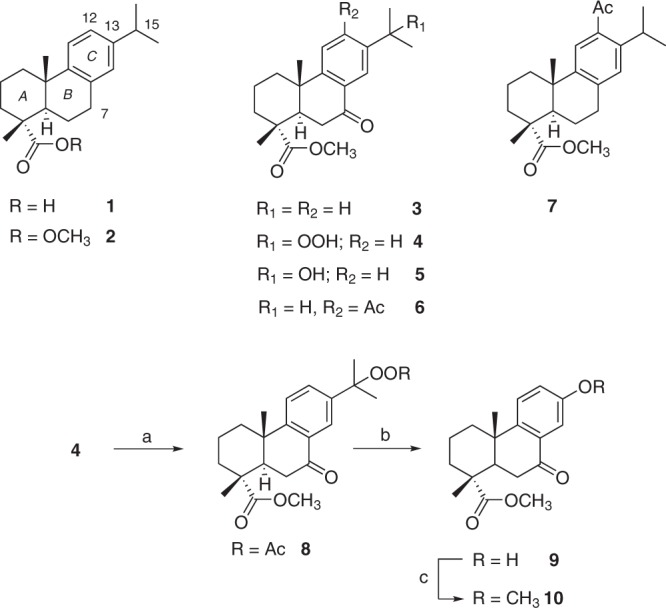
Oxidised derivatives of **1**. Reagents and conditions. (**a**) Ac_2_O, DMAP, CH_2_Cl_2_, r.t.; (**b**) CH_3_COOH, reflux; (**c**) CH_3_I, K_2_CO_3_, DMF, r.t.

Dehydroabietic acid (**1**, Fig. 1) is an aromatic abietane-type diterpenoid which has been reported to possess anticancer activities against several cancer types, and using it as a starting material in an attempt to improve its bioactive properties, a number of semi-synthetic derivatives have been produced^19,20^. However, to the best of our knowledge, very little is known about the potential effects of **1** and its derivatives against pancreatic cancer. More importantly, **1** is an agonist of the peroxisome proliferator-activated receptor γ (PPAR-γ) and suppresses the production of pro-inflammatory mediators, such as monocyte chemoattractant protein-1 (MCP-1/CCL2), tumour necrosis factor α (TNF-α) and nitric oxide (NO), making it potentially relevant for the treatment of cancer-related inflammation^23,24^. Thus, **1** is a highly promising molecular scaffold for the development of innovative multifunctional drugs for the treatment and prevention of cancer. Herein we report the design, synthesis and evaluation of **1** and a panel of its semisynthetic derivatives against pancreatic cancer cells. We tested the ability of the compounds to block inflammation and induce cancer differentiation and carried out target deconvolution studies to propose a possible mode of action for the most promising compounds in the study.

## Results and Discussion

### Oxidised derivatives of 1 inhibit the proliferation of pancreatic cancer cells and supress NO formation

We first tested our in-house library of oxidised derivatives of **1** (Fig. 1)^25^ for their potential to inhibit the proliferation of human Aspc-1 and mouse PanAsc 2159 pancreatic cancer cell lines using the MTT (3-(4,5-dimethylthiazol-2-yl)-2,5-diphenyltetrazolium bromide) assay (Table 1). As the inflammatory process within the tumour microenvironment has been demonstrated to have a major role in the pathogenesis and progression of cancer^26^, we also tested the ability of the compounds to block the formation of NO, an important mediator of inflammation, using mouse macrophage-like cells (RAW 264.7) treated with various concentrations of the compounds and stimulated with interferon-γ (INFγ). The starting material **1** was inactive against all the tested cell lines, even when tested at the high concentration of 60 μM. Among the oxidised products we found that only **3** and **6**, two ester derivatives bearing a carbonyl group at position 7 were active against the tested cell lines, yet none of the prepared compounds inhibited the production of pro-inflammatory NO, representing still a modest improvement in activity compared to the parent compound.

**Table 1:** Anti-proliferative and anti-inflammatory activity screening of the compounds in this study.

Compound^a^	IC_50_ (μM)		
	PanAsc 2159^b^	Aspc-1^b^	NO^c^
**3**	28.3 ± 3.1	31.0 ± 0.4	N.A.^d^
**5**	N.A.	19.3 ± 1.8	N.A.
**6**	27.6 ± 5.0	22.5 ± 3.6	N.A.
**12**	22.9 ± 1.9	20.0 ± 2.4	23.0 ± 0.3
**13**	22.0 ± 2.3	20.3 ± 2.7	23.5 ± 0.2
**14**	N.A.	N.A.	27.8 ± 0.7
**15**	34.0 ± 3.5	30.3 ± 3.6	31.2 ± 2.0
**17**	38.5 ± 7.1	35.9 ± 3.0	32.9 ± 0.6
**20**	23.9 ± 4.4	N.A.	29.9 ± 1.3
**23**	10.6 ± 3.0	8.6 ± 1.1	6.8 ± 1.6
**24**	15.9 ± 4.6	14.0 ± 1.1	12.2 ± 4.6
**25**	11.2 ± 3.5	8.6 ± 2.6	11.6 ± 1.6
**26**	14.8 ± 1.4	8.9 ± 1.2	1.1 ± 0.6
**29**	16.4 ± 1.1	13.1 ± 0.6	10.8 ± 2.8

^a^Compounds **1, 4, 7, 9, 10, 18, 21, 22, 28, 27, 30** and **31** were inactive against all parameters measured at the highest concentration tested (60 μM). ^b^IC_50_ = Concentration of compound that inhibits 50% of cellular growth. IC_50_ values were determined by MTT assay after 72 hours of treatment. The values are the mean ± SD of at least three independent experiments. Pancreatic cancer cell lines: Aspc-1 (human) and PanAsc 2159 (mouse). ^c^IC_50_ =Concentration of compound that inhibits the formation of NO by 50%. IC_50_ values were determined using RAW 246.7 mouse macrophage-like cells after treatment with the compound for 20 minutes and stimulation with INFγ (10 ng/mL) for 24 hours. The values are the mean ± SD of at least three independent experiments. ^d^N.A. = not active at 60 μM.

### 7-Pyridyl derivatives of 1 have improved anti-proliferative and anti-inflammatory profiles against pancreatic cancer cells

**Figure 2:**
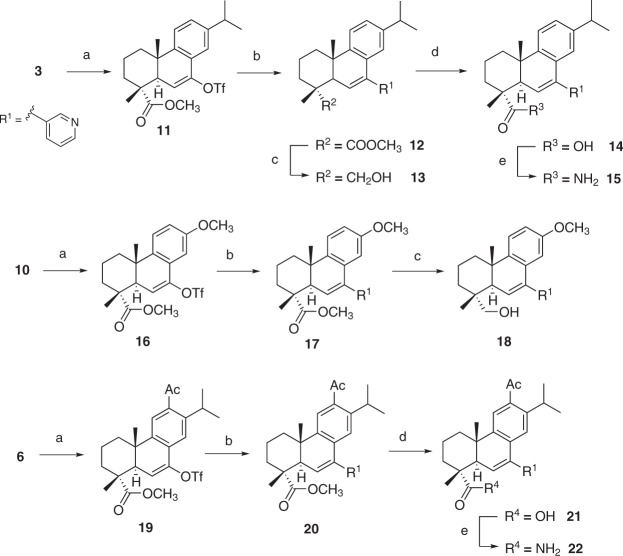
Synthesis of pyridyl derivatives **12–15**, **17**, **18** and **20–22**. Reagents and conditions: (**a**) Tf_2_O, DTBMP, CH_2_Cl_2_, reflux; (**b**) (Ph_3_P)_2_PdCl_2_, diethyl(3-pyridyl)borane, Na_2_CO_3_ in H_2_O (0.8 M), THF, reflux; (**c**) LiAlH_4_, anhydrous THF, 0 °C to r.t. (**d**) KOH, ethylene glycol/water 10:1, 120 °C; (**e**) EDC·HCl, HOBt, NH_3_, DMF, 0 °C to r.t.

A recent analysis revealed that 59% of all small-molecule drugs contain a nitrogen heterocycle, clearly demonstrating their relevance as privileged moieties among pharmaceuticals^27^. In this analysis, the top three spots were ruled by pyridine, piperidine, and piperazine. Indeed, pyridyl groups tend to increase metabolic stability^28^ and have been used to improve the drug-like properties of natural products^29^. Moreover, pyridyl group additions to several terpenoids have resulted in an improvement of their anticancer properties^28^. Therefore, we hypothesized that coupling of a nitrogen heterocycle to **1** could result in a set of compounds with improved bioactivity. Insertion of a pyridyl group at C7 of compounds **3**, **6** and **10** was accomplished by Suzuki cross-coupling (Fig. 2) via the preparation of the corresponding vinyl triflate intermediates **11**, **16** and **19**^30^. The preparation of **10** is depicted in Fig. 1 and proceeded via a two-step procedure for removal of the C13 isopropyl side chain of **4** to give **9**, followed by methylation. Coupling of the heterocycle was made with bis(triphenylphosphine)palladium(II) dichloride and diethyl(3-pyridyl)borane, in the presence of base (Fig. 2)^30^. Compounds **12**, **17** and **20** were obtained in 63%, 74% and 61% yields, respectively, after chromatographic purification. Reduction of the ring A ester in **12** and **17** with LiAlH_4_ gave **13** and **18** whereas hydrolysis with KOH in a mixture of ethylene glycol and water (10:1) gave **21** and **14** that were further converted into **15** and **22** through carbodiimide coupling.

**Figure 3:**
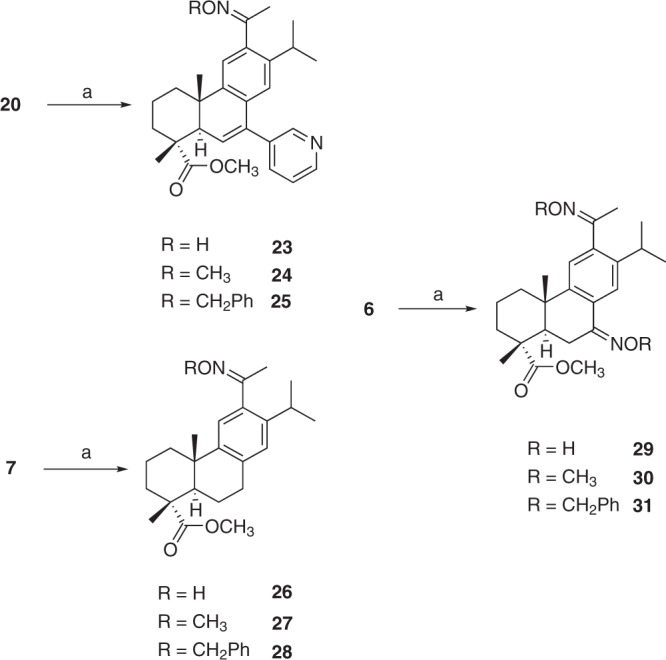
Synthesis of oxime derivatives **23–31**. Reagents and conditions: (a) H_2_NOR**·**HCl, pyridine, EtOH, reflux.

In this set of compounds, the esters **12** and **17**, the alcohol **13** and the amide **15** were active against both pancreatic cell lines with IC_50_ values ranging from of 22.0 to 38.5 μM that correlated well with their ability to inhibit NO production in mouse macrophages (Table 1). The same effect was, however, not observed for the 12-acetyl derivatives **21** and **22** and among the 12-substituted compounds prepared. Only **20** was active against mouse PanAsc 2159 cells with an IC_50_ value of 23.9 μM and could in addition block NO production within the same concentration range. The presence of a carboxyl group in ring A (**14** and **21**) had a negative impact on the anti-proliferative activity of these compounds; however, the ability to inhibit NO production was retained in **14**. Overall, the presence of the pyridyl group proved advantageous for the activity of the compounds derivatised from the initial in-house set. Nonetheless, the observed potency was still in the high micromolar range and thus further synthetic efforts were placed to produce an additional set of compounds (Fig. 3).

### Oximes derivatives of 1 are potent anti-proliferative and anti-inflammatory compounds against pancreatic cancer cells

Oximes are well-known to interfere with reactive oxygen species and many oximes and *O*-substituted oximes have been reported to have anti-inflammatory and anticancer properties, including against multi-drug resistant cancer cell lines^31–33^. Derivatives **20** (Fig. 2), **6** and **7** (Fig. 1), bearing a 12-acetyl group, were selected for further modification by conversion of carbonyl groups into either a simple oxime or an *O*-substituted oxime of increasing bulkiness according to Fig. 3. Compounds **23–31** were synthesised via reaction with hydroxylamine hydrochloride or the corresponding *O*-alkyl hydroxylamine in the presence of pyridine.

Except for compound **28**, which consists of a mixture of isomers, all other compounds were obtained as a single isomer after chromatographic purification. Introduction of an oxime at position 12 clearly increased the anti-proliferative activity against the pancreatic cancer cell lines as well as the inhibition of NO production, with and without the pyridyl group at position 7 (Table 1). Compound **26** displayed the most potent anti-inflammatory effects, with an IC_50_ value of 1.1 μM. In Aspc-1 cells, the IC_50_ values were reduced to low micromolar range in **23** (8.6 μM) and **26** (8.9 μM). The introduction of bulkier oximes in **27**, **28**, **30** and **31** resulted in inactive compounds. However, in the presence of the 7-pyridyl group, **24** and **25** showed improved activity in all assays with IC_50_ values ranging from 8.6 to 15.9 μM.

### Dehydroabietic oximes arrest pancreatic cancer cell growth in the G1 phase by up-regulation of p27 and down-regulation of cyclin D1 levels

**Figure 4:**
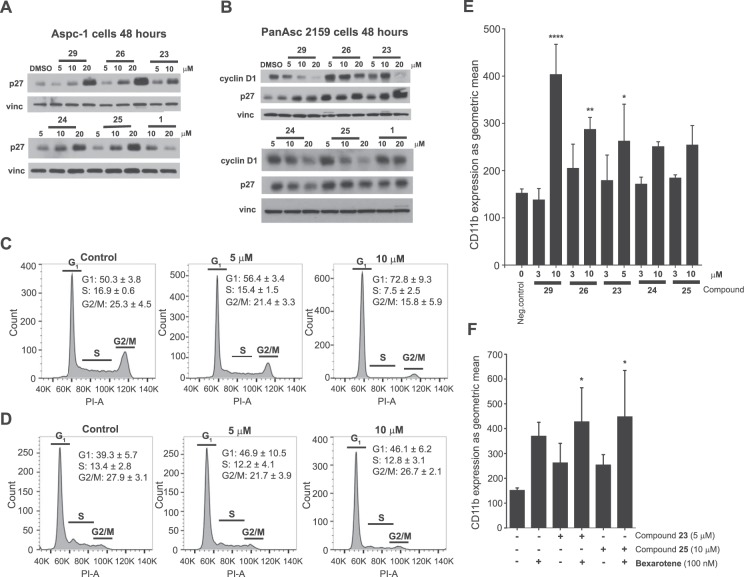
Effects of compounds **23–26**, **29** and **1** on the expression of cell cycle proteins p27 in Aspc-1 cells (**A**) and p27 and cyclin D1 in PanAsc 2159 cells (**B**). Blots have been grouped from different parts of the same gels. Full-length blots and multiple exposures are found in the Supplementary Information. For compound **23**, concentration 20 μM was cropped out (from Aspc-1 treatment) as there was not enough protein to get a comparable result (most cells died at 20 μM). Cell cycle analysis of pancreatic cancer cells treated with **29**. Aspc-1 (**C**) and PanAsc 2159 (**D**) cells were treated with **29** at 5 and 10 μM for 48 hours. The histogram of one experiment is illustrated (results for all compounds are in the supporting info). Results for the different stages of cell cycle are indicated for 3–4 independent experiments (mean ± SEM). Ability of compounds **23–26**, **29** and **1** to induce cancer cell differentiation in U937 cells alone (**E**) and ability of compounds **23** and **25** to induce cancer cell differentiation in U937 cells in combination with bexarotene (**F**). *P < 0.05, **P < 0.01, ****P < 0.0001 vs. vehicle (DMSO) alone. Vinc: vinculin (loading control).

To define whether the growth inhibitory properties of the compounds were associated with regulation of the cell cycle, western blot and cell cycle analyses were made with five selected compounds from the *in vitro* assays namely, the oximes **23–26** and **29** (Fig. 4). PanAsc 2159 and Aspc-1 cells were treated for 48 hours with the compounds and the protein expression of p27 and cyclin D1 was investigated. p27 is a cyclin-dependent kinase inhibitor that regulates the activity of cyclin-dependent kinases (CDKs). Low levels of p27 are generally associated with several cancers, including that of the pancreas^34^. The amount of cyclin D1 is important for the cells to progress from the G1 phase forward into S phase and start copying their DNA. If the balance of stimulatory versus inhibitory signals is towards cell growth and division the amount of cyclin D1 increases which leads to its binding to CDK4 and CDK6. Overexpression of cyclin D1 is common in pancreatic cancer^35,36^.

The western blot analysis on PanAsc 2159 cells (Fig. 4B) revealed a dose-dependent down-regulation of cyclin D1 for and induction of p27 after treatment with **23**, **26**, **29** however not with **24** and **25**. Compound **1** did not affect any of the measured markers even at the high concentration of 20 μM. Aspc-1 cells (Fig. 4A) did not express cyclin D1 but p27 was up-regulated in a dose-dependent manner for all tested compounds apart from **1** where the effect was the opposite at 20 μM. The cell cycle analysis on Aspc-1 cells showed that with compounds **23–26** and **29**, especially at 10 μM concentration, the majority of the cells are in the G1 phase. The histogram (Fig. 4C) shows the results for compound **29** vs. control. In PanAsc 2159 cells (Fig. 4D) a similar trend is observed, although not so evident. These findings support the results from the anti-proliferation assays and show that the growth inhibitory properties are likely due to cell cycle arrest in G1 phase, observed in the cell cycle analysis and expressed here as changed levels of cyclin D1 and p27. Of note, for compound **23**, only 5 and 10 μM samples were included for the treatment of Aspc-1 cells in western blot analysis because at 20 μM many cells died. At this concentration, cleaved poly (ADP-ribose) polymerase (PARP) was clearly observed, suggesting that cells were dying by apoptosis (data not shown).

### Dehydroabietic oximes promote cancer cell differentiation

Regulating cell differentiation is highly desirable in the context of cancer prevention and treatment^37^. The ability to induce malignant cells to overcome their block of differentiation in order to undergo apoptosis may circumvent the need for a direct cytotoxic effect of drugs and has been correlated with a higher possibility of achieving complete remission and cure. In order to evaluate the ability of the compounds to induce monocyte differentiation, we used human leukaemia cells as a model and CD11b, a cell surface antigen, as a differentiation biomarker. This marker is weakly expressed on leukaemia U937 cells but can be induced with various established differentiation agents such as retinoids or rexinoids.

After 4 days of treatment with the compounds, the expression of CD11b was measured by flow cytometry. As shown in Fig. 4, induction of the expression of CD11b occurred after treatment with all compounds, in a dose-dependent manner, when compared with the control. Compound **29** was the most efficient in inducing CD11b expression, which was particularly evident at 10 μM. Compounds **23** and **25** were also combined with bexarotene, the only FDA-approved rexinoid for the treatment of cutaneous T-cell lymphoma, which has well-established cell differentiation and growth suppression effects^38,39^, to look for possible synergism. However, no synergistic effects were observed in combination with bexarotene.

### Dehydroabietic oximes target the p90 ribosomal S6 kinase 2 (RSK2)

The oxime derivatives **23** and **26**, differing at the C7 position by the presence of the pyridyl group in **23**, were screened against a comprehensive panel of 140 kinases at the International Centre for Kinase Profiling (ICKP), in Dundee, UK (Table S1, Supporting information). Neither compound was found to be a very potent kinase inhibitor as the maximum level of enzyme inhibition was 30–40% at the single concentration tested of 10 μM. However, **26** stood out as being able to inhibit a single kinase by 35%, namely p90 ribosomal S6 kinase 2 (RSK2), an AGC kinase of the RSK family^40,41^. Notably, concomitant inhibition of RSK1, one of the 4 known isoforms, was not observed. In contrast, the 7-pyridyl containing **23** displayed, in addition to RSK2 inhibition, comparable activity against of a few other kinases especially protein kinase PKBβ (also an AGC kinase), as well as inhibitor of nuclear factor kappa-B kinase β (IKKβ), mammalian sterile 20-like 2 (MST2), proto-oncogene tyrosine kinase protein (Src) and Bruton agammaglobulinemia tyrosine kinase (BTK).

RSKs 1–4 are important downstream mediators of the Ras-ERK signal transduction pathway which, when activated, phosphorylate many substrates, including p27, in both the cytoplasm and nucleus, resulting in cell differentiation, survival, growth and proliferation^40–42^. RSK2 is abundant in the skeletal muscle, heart and pancreas, and associates with T-cell-restricted intracellular antigen-1 (TIA-1) to localize to stress granules upon oxidative stress. RSK2 can also be activated by phosphorylation on tyrosine residues in response to fibroblast growth factor (FGF) receptor and Src activation. Inhibition of RSKs has been implicated in prevention of cellular invasion and metastasis. Currently there are four classes of RSK inhibitors reported in the literature^41–44^. However, none is selective towards single enzyme isoforms and addressing isoform selectivity to differentiate between the specific role of each RSK in cancer remains a challenge. In this regard, the selective effect of compound **26** on RSK2 activity seems very promising. In addition, the loss of selectivity in **23** provides important preliminary data to small chemical modifications around the diterpenoid core that can greatly impact this parameter. Thus, dehydroabietic oximes can be regarded as a new chemical class with potential to develop isoform-selective RSK inhibitors. In particular, concomitant inhibition of RSK2 and PKBβ will need to be tackled due to the specific role of the latter in insulin signalling^40^.

## Conclusion

Abietane-type diterpenoids such as **1** are excellent scaffolds to develop in search for innovative multifunctional agents for cancer treatment and prevention. In this study, we revealed that dehydroabietic oximes in particular, have multiple desirable actions on cancer cells including anti-proliferative, anti-inflammatory and differentiation-inducing effects. These effects are mediated by depletion of cell cycle proteins such as cyclin D1 and induction of key proteins typically downregulated in many cancers such as p27. Selective inhibition of the AGC kinase RSK2 was also observed, however modest. Nonetheless, this finding paves the way for future investigations of this class of compounds as potential isoform-selective inhibitors of RSKs, which will help to shed light onto their specific roles in cell death and survival. Taken together, our findings suggest that compounds such as dehydroabietic oximes are worth pursuing when searching for novel therapies for cancers such as those of the pancreas, where there is the need to target multiple pathways concomitantly for a better chance of therapeutic success.

## Methods

### Chemistry

Commercially available reagents and solvents were used without further purification. Reagents were obtained from Pfaltz & Bauer, Sigma Aldrich Co, VWR International Oy or Fluorochem Ltd. All reactions were monitored with thin layer chromatography (TLC) using Silica gel 60 F254 TLC plates. Flash column chromatography (FCC) was made with a Biotage High-Performance Flash Chromatography Sp4-system (Uppsala, Sweden) using a 0.1-mm path length flow cell UV detector/recorder module (fixed wavelength: 254 nm), and 10 g, 25 g or 50 g SNAP cartridges (10–50 mL/min flow rate). Infra-red (IR) spectra were obtained using a Vertex 70 (Bruker Optics Inc., MA, USA) FTIR instrument. The measurements were made with a horizontal attenuated total reflectance (ATR) accessory (MIRacle, Pike Technology, Inc, WI, USA). The transmittance spectra were recorded at a 4 cm^−1^ resolution between 4000 and 600 cm^−1^ using the OPUS 5.5 software (Bruker Optics Inc., MA, USA). Nuclear magnetic resonance (NMR) spectra were obtained using a Varian Mercury Plus 300 spectrometer or Bruker Ascend 400 spectrometer, in CDCl_3_, with tetramethylsilane (TMS) as the internal standard. The chemical shifts were reported in parts per million (ppm) and on the *δ* scale from tetramethylsilane (TMS) as an internal standard. The coupling constants *J* are quoted in Hertz (Hz). ESI-MS was performed by direct injection or UPLC-MS using a Synapt G2 HDMS (Waters, Milford MA, USA) instrument. Compounds **9–25** and **27–31** are novel. The synthesis of compounds **2–7** was reported previously^23^.

### Methyl 13-hydroxy-7-oxo-podocarpa-8,11,13-trien-18-oate (9)

To a stirred solution of **4** (0.960 g, 2.66 mmol) in dichloromethane (15 mL) was added 4-dimethylaminopyridine (DMAP, 195 mg, 1.60 mmol) and acetic anhydride (1.63 g, 16.0 mmol). The solution was stirred at room temperature for 24 h after which it was diluted with diethyl ether (90 mL) and water was added (30 mL). The aqueous phase was extracted with diethyl ether (2 × 40 mL) and the combined organic phase was washed with water (40 mL) and brine (30 mL) and dried over anhydrous Na_2_SO_4_. After removal of the solvent under reduced pressure a crude compound **8** (1.32 g) was obtained. The crude product was dissolved in acetic acid (10 mL) and the temperature raised to 100 °C. After stirring the solution for 3 h, acetic acid was evaporated and water (30 mL) and diethyl ether (50 mL) were added. The aqueous phase was extracted with diethyl ether (2 × 35 mL) and the combined organic phase was washed with a saturated aqueous solution of NaHCO_3_ (25 mL), water (25 mL) and brine (20 mL) and dried over anhydrous Na_2_SO_4_. After removal of the solvent under reduced pressure, the resulting crude product was purified by flash chromatography on silica gel using ethyl acetate in *n*-hexane (0 to 100%) as the eluent giving compound **9** (510 mg, 63%) as a yellowish solid. ^1^H NMR (300 MHz, CDCl_3_) *δ* 1.24 (d, *J* = 0.7 Hz, 3 H), 1.34 (s, 3 H), 1.70 (m, 6 H), 2.35 (m, 2 H), 2.71 (m, 2 H), 3.66 (s, 3 H), 5.87 (s, 1 H), 7.07 (dd, *J = *8.6, 2.9 Hz, 1 H), 7.25 (d, *J* = 8.6 Hz, 1 H), 7.52 (d, *J* = 2.9 Hz, 1 H). ^13^C NMR (75 MHz, CDCl_3_) *δ* 16.5, 18.3, 23.9, 36.8, 37.3, 37.4, 38.0, 44.2, 46.8, 52.4, 113.0, 122.1, 125.3, 132.0, 148.3, 154.5, 178.1, 198.8. IR (ATR) 3375, 2947, 1722, 1666, 1605, 1440, 1221, 835 cm^−1^. HRMS *m/z*: calcd. for C_25_H_32_NO 303.1596 [M + H]^+^, found 303.1597.

### Methyl 13-methoxy-7-oxo-podocarpa-8,11,13-trien-18-oate (10)

To a stirred solution of **9** (61.6 mg, 0.204 mmol) and K_2_CO_3_ (70.4 mg, 0.509 mmol) in DMF (60 mL), MeI (25 μL, 0.41 mmol) was added. The solution was stirred at room temperature for 5 h. The solution was diluted with diethyl ether (20 mL) and a 1 M aqueous solution of HCl (2 mL) was carefully added. The aqueous phase was extracted with diethyl ether (2 × 10 mL) and the combined organic phase was washed with a 1 M aqueous solution of HCl (10 mL), a saturated aqueous solution of NaHCO_3_ (10 mL), water (10 mL) and brine (10 mL) and dried over anhydrous Na_2_SO_4_. After removal of the solvent under reduced pressure, the resulting crude product was purified by flash chromatography on silica gel using ethyl acetate in *n*-hexane (0 to 100%) as the eluent giving compound **10** (57 mg, 88%) as a colourless oil. ^1^H NMR (400 MHz, CDCl_3_) *δ* 1.25 (s, 3 H), 1.34 (s, 3 H), 1.74 (m, 5 H), 2.34 (m, 2 H), 2.71 (m, 2 H), 3.66 (s, 3 H), 3.83 (s, 3 H), 7.10 (dd, *J* = 8.7, 3.0 Hz, 1 H), 7.29 (d, *J* = 8.7 Hz, 1 H), 7.48 (d, *J* = 2.9 Hz, 1 H). ^13^C NMR (75 MHz, CDCl_3_) *δ* 16.5, 18.3, 23.9, 36.7, 37.3, 37.4, 37.9, 44.2, 46.8, 52.3, 55.6, 109.7, 122.2, 125.0, 131.9, 148.4, 158.1, 165.6, 177.9, 198.2. IR (ATR) 2943, 1722, 1680, 1246, 1111, 1032, 829 cm^−1^. HRMS *m/z*: calcd. for C_19_H_25_O_4_ 317.1753 [M + H]^+^, found 317.1754.

### Methyl 7-[[(trifluoromethyl)sulfonyl]oxy]-abieta-6,8,11,13-tetraen-18-oate (11)

To an argon-flushed flask with **3** (0.337 g, 1.03 mmol) in dichloromethane (10 mL) were added trifluoromethanesulfonic anhydride (Tf_2_O, 209 μL, 1.24 mmol) and 2,6-di-*tert*-butyl-4-methylpyridine (DTBMP, 295 mg, 1.44 mmol) after which temperature was raised to reflux. The resulting solution was stirred for 3 h, at reflux, after which the solvent was evaporated and the residue was diluted with diethyl ether (60 mL) and water (25 mL). The aqueous phase was extracted with ethyl acetate (3 × 60 mL) and the combined organic phases were washed with a 1 M aqueous solution of HCl (2 × 30 mL), a saturated aqueous solution of NaHCO_3_ (2 × 30 mL), water (30 mL), and brine (30 mL), and dried over anhydrous Na_2_SO_4_. After removal of the solvent under reduced pressure, the resulting crude product was purified by flash chromatography on silica gel using ethyl acetate in *n*-hexane (0 to 40%) as the eluent giving compound **11** (404 mg, 85%) as a colourless oil. ^1^H NMR (300 MHz, CDCl_3_) *δ* 1.15 (s, 3 H), 1.24 (d, *J* = 6.9 Hz, 6 H), 1.42 (s, 3 H), 1.75 (m, 6 H), 2.19 (m, 2 H), 2.90 (sept, *J* = 6.9 Hz, 1 H), 3.03 (d, *J* = 2.9 Hz, 1 H), 3.67 (s, 3 H), 5.75 (d, *J* = 2.9 Hz, 1 H), 7.12 (d, *J* = 8.0 Hz, 1 H), 7.20 (dd, *J* = 8.0, 1.9 Hz, 1 H), 7.23 (d, *J* = 1.7 Hz, 1 H). ^13^C NMR (75 MHz, CDCl_3_) *δ* 18.2, 18.4, 21.0, 23.8, 24.1, 33.9, 35.3, 35.6, 37.8, 46.3, 46.6, 52.4, 116.7, 119.6, 120.1, 121.0, 122.3, 127.5, 128.3, 145.6, 145.9, 147.2, 177.8. IR (ATR) 2957, 1728, 1420, 1205, 1138, 1011, 827 cm^−1^. HRMS *m/z*: calcd. for C_22_H_28_O_5_SF_3_ 461.1610 [M + H]^+^, found 461.1610.

### Methyl 7-pyridyl-abieta-6,8,11,13-tetraen-18-oate (12)

To an argon-flushed flask with **11** (0.208 g, 0.452 mmol) in THF (6 mL) were added bis(triphenylphosphine)palladium(II) dichloride (22.2 mg, 0.0316 mmol) and diethyl(3-pyridyl)borane (99.5 mg, 0.677 mmol). The temperature was raised to 45 °C and a 0.8 M aqueous solution of Na_2_CO_3_ (2.2 mL, 3.84 mmol) was added with vigorous stirring. The temperature was further raised to 75 °C and stirring continued for 3.5 h. Solvents were evaporated and the residue was diluted with diethyl ether (30 mL) and a 1 M aqueous solution of HCl was added (5 mL). The aqueous phase was extracted with diethyl ether (2 × 25 mL), and the combined organic phases were washed with a 1 M aqueous solution of HCl (10 mL), water (20 mL) and brine (10 mL), and dried over anhydrous Na_2_SO_4_. After removal of the solvent under reduced pressure, the resulting crude product was purified by flash chromatography on silica gel using ethyl acetate in *n*-hexane (0 to 80%) as the eluent giving compound **12** (131 mg, 74%) as a white solid. ^1^H NMR (300 MHz, CDCl_3_) *δ* 1.16 (m, 9 H), 1.41 (s, 3 H), 1.79 (m, 6 H), 2.24 (m, 1 H), 2.77 (sept, *J* = 6.9 Hz, 1 H), 2.98 (d, *J* = 3.1 Hz, 1 H), 3.70 (s, 3 H), 5.84 (d, *J* = 3.1 Hz, 1 H), 6.81 (d, *J* = 1.9 Hz, 1 H), 7.13 (dd, *J* = 8.0, 1.9 Hz, 1 H), 7.18 (d, *J* = 8.0 Hz, 1 H), 7.31 (ddd, *J* = 7.9, 4.8, 0.9 Hz, 1 H), 7.66 (dt, *J* = 7.8, 2.0 Hz, 1 H), 8.60 (m, 2 H). ^13^C NMR (75 MHz, CDCl_3_) *δ* 18.2, 18.5, 21.2, 24.0, 24.1, 33.8, 35.8, 35.8, 37.4, 46.4, 47.1, 52.2, 122.1, 123.2, 124.3, 126.2, 130.6, 132.7, 136.1, 136.4, 136.8, 146.2, 146.5, 148.6, 149.8, 178.4. IR (ATR) 2953, 1724, 1223, 1124, 827, 808 cm^−1^. HRMS *m/z*: calcd. for C_26_H_32_NO_2_ 390.2433 [M + H]^+^, found 390.2437.

### 7-Pyridyl-abieta-6,8,11,13-tetraen-18-ol (13)

To a suspension of lithium aluminium hydride (21.9 mg, 0.578 mmol) in anhydrous THF (2 mL) was added dropwise, at 0 °C, a solution of **12** (0.150 g, 0.385 mmol) in anhydrous THF (2 mL). The suspension was stirred at room temperature for 2.5 h after which the reaction mixture was quenched with methanol (1 mL). Solvents were evaporated and the residue was diluted with diethyl ether (15 mL) and ethyl acetate (5 mL) and water (10 mL). Aqueous phase was extracted with diethyl ether (3 × 15 mL) and the combined organic phases were washed with water (3 × 15 mL) and dried over anhydrous Na_2_SO_4_. After removal of the solvent under reduced pressure, the resulting crude product was purified by flash chromatography on silica gel using ethyl acetate in *n*-hexane (0–80%) as the eluent to give the product **13** as a white solid (49 mg, 35%). ^1^H NMR (400 MHz, CDCl_3_) *δ* 1.04 (s, 3 H), 1.15 (m, 9 H), 1.50 (m, 2 H), 1.65 (td, *J* = 12.7, 4.3 Hz, 1 H), 1.81 (m, 2 H), 1.98 (br. s, 1 H), 2.22 (dt, *J* = 12.7, 3.2 Hz, 1 H), 2.50 (d, *J* = 3.1 Hz, 1 H), 2.77 (sept, *J* = 7.0 Hz, 1 H), 3.34 (d, *J* = 11.0 Hz, 1 H), 3.55 (d, *J* = 11.0 Hz, 1 H), 6.05 (d, *J* = 3.1 Hz, 1 H), 6.81 (d, *J* = 1.9 Hz, 1 H), 7.13 (dd, *J* = 8.0, 1.9 Hz, 1 H), 7.19 (d, *J* = 8.0 Hz, 1 H), 7.31 (dd, *J* = 7.9, 4.8 Hz, 1 H), 7.68 (dt, *J* = 7.8, 2.0 Hz, 1 H), 8.57 (dd, *J* = 4.9, 1.7 Hz, 1 H), 8.63 (d, *J* = 2.2 Hz, 1 H). ^13^C NMR (75 MHz, CDCl_3_) *δ* 18.4, 18.5, 21.2, 24.0, 24.1, 33.8, 34.7, 36.2, 37.5, 37.6, 45.7, 71.7, 122.2, 123.2, 124.3, 126.2, 129.9, 132.8, 136.1, 136.9, 137.0, 146.3, 146.8, 148.5, 149.7. IR (ATR) 3258 (br.), 2924, 1410, 1057, 808, 717 cm^−1^. HRMS *m/z*: calcd. for C_25_H_32_NO 362.2484 [M + H]^+^, found 362.2487.

### 7-Pyridyl-abieta-6,8,11,13-tetraen-18-oic acid (14)

A mixture of **12** (0.415 g, 1.07 mmol) and potassium hydroxide (0.179 g, 3.20 mmol) in ethylene glycol/water 10:1 (330 μL) was heated to 120 °C. After 4.5 h, diethyl ether (30 mL) and water (20 mL) were added. The aqueous phase was extracted with diethyl ether (3 × 15 mL). Some of the product remained in the aqueous phase so the aqueous phase was acidified with a 1 M aqueous solution of HCl and extracted again with diethyl ether (2 × 15 mL). The combined organic phases were washed with water (50 mL) and brine (40 mL) and dried over anhydrous Na_2_SO_4_. After removal of the solvent under reduced pressure, the resulting crude product was purified by flash chromatography on silica gel using ethyl acetate in *n*-hexane (0 to 100%) as the eluent to give compound **14** as a white solid (337 mg, 84%). ^1^H NMR (300 MHz, CDCl_3_) *δ* 1.11 (m, 6 H), 1.19 (s, 3 H), 1.49 (s, 3 H), 1.81 (m, 6 H), 2.24 (d, *J = *12.5 Hz, 1 H), 2.74 (sept, *J* = 6.9 Hz, 1 H), 3.07 (d, *J* = 3.1 Hz, 1 H), 6.14 (d, *J* = 3.1 Hz, 1 H), 6.73 (d, *J* = 1.9 Hz, 1 H), 7.11 (dd, *J* = 8.0, 1.9 Hz, 1 H), 7.18 (d, *J* = 8.0 Hz, 1 H), 7.37 (ddd, *J* = 7.9, 5.0, 0.8 Hz, 1 H), 7.76 (dt, *J* = 7.9, 1.9 Hz, 1 H), 8.55 (dd, *J* = 4.9, 1.6 Hz, 1 H), 8.77 (dd, *J* = 2.3, 0.8 Hz, 1 H).^13^C NMR (75 MHz, CDCl_3_) *δ* 18.5, 18.7, 21.3, 24.0, 24.1, 33.8, 36.0, 36.1, 37.4, 45.9, 47.3, 122.2, 123.3, 124.0, 126.2, 132.6, 132.8, 135.4, 137.6, 137.7, 146.2, 146.3, 146.5, 148.1, 181.2. IR (ATR) 2926, 2478 (br.), 1909 (br.), 1699, 1263, 1128, 810, 713 cm^−1^. HRMS *m/z*: calcd. for C_25_H_30_NO_2_ 376.2277 [M + H]^+^, found 375. 376.2282.

### 7-Pyridyl-abieta-6,8,11,13-tetraen-18-amide (15)

Compound **14** (0.150 g, 0.399 mmol) was dissolved in DMF (2 mL) followed by the addition of 1-ethyl-3-(3-dimethylaminopropyl)carbodiimide (EDC) hydrochloride (84.2 mg, 0.439 mmol) and 1-hydroxybenzotriazole (HOBt) monohydrate (59.3 mg, 0.439 mmol), at 0 °C. After stirring the solution for 5 min at 0 °C and 35 min at room temperature, a 25% aqueous solution of ammonia (154 μL, 1.00 mmol) was added. The reaction mixture was stirred for additional 23 h at room temperature after which the reaction was quenched with a mixture of water (10 mL) and ethyl acetate (20 mL). The aqueous phase was extracted with ethyl acetate (3 × 20 mL), and the combined organic phases were washed with a 1 M aqueous solution of HCl (2 × 20 mL), water (20 mL), and brine (20 mL) and dried over anhydrous Na_2_SO_4_. After removal of the solvent under reduced pressure, compound **15** (78 mg, 52%) was obtained as a white solid. ^1^H NMR (300 MHz, CDCl_3_) *δ* 1.15 (m, 6 H), 1.19 (s, 3 H), 1.43 (s, 3 H), 1.79 (m, 6 H), 2.26 (m, 1 H), 2.77 (sept, *J* = 7.0 Hz, 1 H), 2.91 (m, 1 H), 5.78 (s, 2 H), 5.88 (d, *J* = 3.1 Hz, 1 H), 6.81 (d, *J* = 1.8 Hz, 1 H), 7.13 (dd, *J* = 8.0, 1.8 Hz, 1 H), 7.18 (d, *J* = 8.0 Hz, 1 H), 7.31 (ddd, *J* = 7.8, 4.9, 0.9 Hz, 1 H), 7.68 (dt, *J* = 7.8, 1.9 Hz, 1 H), 8.58 (dd, *J* = 4.8, 1.7 Hz, 1 H), 8.62 (d, *J* = 1.9 Hz, 1 H).^13^C NMR (75 MHz, CDCl_3_) *δ* 18.3, 18.7, 21.2, 24.0, 24.1, 33.8, 35.8, 36.9, 37.5, 46.1, 47.4, 122.1, 123.4, 124.4, 126.4, 130.1, 132.6, 136.4, 137.2, 146.2, 146.6, 148.3, 149.4, 181.0. IR (ATR) 3335, 3182, 2928, 1659, 808, 716 cm^−1^. HRMS *m/z*: calcd. for C_25_H_31_N_2_O 375.2436 [M + H]^+^, found 375.2436.

### Methyl 7-[[(trifluoromethyl)sulfonyl]oxy]-13-methoxy-podocarpa-6,8,11,13-tetraen-18-oate (16)

Following the procedure for compound **11**, compound **16** was prepared from **10** (0.517 g, 1.63 mmol), Tf_2_O (333 μL, 1.98 mmol) and DTBMP (0.470 g, 2.29 mmol) in dichloromethane (16 mL). Compound **16**: colourless oil (581 mg, 79%). ^1^H NMR (300 MHz, CDCl_3_) *δ* 1.13 (d, *J* = 0.7 Hz, 3 H), 1.41 (s, 3 H), 1.74 (m, 6 H), 2.18 (m, 2 H), 3.02 (d, *J* = 2.9 Hz, 1 H), 3.68 (s, 3 H), 3.80 (s, 3 H), 5.79 (d, *J* = 2.9 Hz, 1 H), 6.86 (dd, *J* = 8.5, 2.7 Hz, 1 H), 6.92 (d, *J = *2.7 Hz, 1 H), 7.11 (d, *J* = 8.5 Hz, 1 H). ^13^C NMR (75 MHz, CDCl_3_) *δ* 18.2, 18.4, 21.2, 35.4, 35.6, 37.7, 46.2, 46.8, 52.4, 55.5, 107.6, 115.6, 116.7, 120.5, 120.9, 123.6, 128.7, 140.5, 145.5, 158.3, 177.8. IR (ATR) 2947, 1726, 1420, 1207, 1138, 1007, 825 cm^−1^. HRMS *m/z*: calcd. for C_20_H_24_O_6_SF_3_ 449.1246 [M + H]^+^, found 449.1248.

### Methyl 13-methoxy-7-pyridyl-podocarpa-6,8,11,13-tetraen-18-oate (17)

Following the procedure for compound **12**, compound **17** was prepared from **16** (0.552 g, 1.23 mmol), (Ph_3_P)_2_PdCl_2_ (60.3 mg, 0.0861 mmol), diethyl(3-pyridyl)borane (271 mg, 1.85 mmol) and 0.8 M Na_2_CO_3_ in H_2_O (5.9 mL, 4.72 mmol) in THF (16 mL). Compound **17**: yellowish solid (292 mg, 63%). ^1^H NMR (400 MHz, CDCl_3_) *δ* 1.16 (s, 3 H), 1.41 (s, 3 H), 1.79 (m, 5 H), 2.23 (d, *J* = 11.5 Hz, 1 H), 2.96 (d, *J* = 3.1 Hz, 1 H), 3.69 (s, 3 H), 3.70 (s, 3 H), 5.86 (d, *J* = 3.0 Hz, 1 H), 6.51 (d, *J* = 2.7 Hz, 1 H), 6.80 (dd, *J* = 8.6, 2.7 Hz, 1 H), 7.17 (d, *J* = 8.6 Hz, 1 H), 7.32 (ddd, *J* = 7.8, 4.9, 0.8 Hz, 1 H), 7.67 (dt, *J* = 7.8, 2.0 Hz, 1 H), 8.59 (dd, *J* = 4.9, 1.7 Hz, 1 H), 8.61 (dd, *J* = 2.3, 0.8 Hz, 1 H). *δ* ^13^C NMR (75 MHz, CDCl_3_) *δ* 18.2, 18.5, 21.4, 35.9, 35.9, 37.2, 46.3, 47.3, 52.3, 55.4, 112.2, 113.1, 123.3, 123.4, 131.7, 134.0, 136.3, 136.4, 136.7, 141.3, 147.9, 149.0, 157.8, 178.4. IR (ATR) 2939, 1722, 1221, 1030, 808, 717 cm^−1^. HRMS *m/z*: calcd. for C_24_H_28_NO_3_ 378.2069 [M + H]^+^, found 378.2069.

### 13-Methoxy-7-pyridyl-podocarpa-6,8,11,13-tetraen-18-ol (18)

Following the procedure for compound **13**, compound **18** was prepared from **17** (0.130 g, 0.344 mmol) and LiAlH_4_ (26.1 mg, 0.689 mmol) in THF (3.5 mL). Compound **18**: white solid (42 mg, 35%). ^1^H NMR (400 MHz, CDCl_3_) *δ* 1.04 (s, 3 H), 1.17 (s, 3 H), 1.48 (m, 1 H), 1.65 (td, *J* = 12.6, 4.6 Hz, 2 H), 1.82 (m, 1 H), 2.21 (d, *J* = 12.6 Hz, 2 H), 2.48 (d, *J* = 3.1 Hz, 1 H), 3.34 (d, *J* = 11.1 Hz, 1 H), 3.55 (d, *J* = 11.1 Hz, 1 H), 3.69 (s, 3 H), 6.07 (d, *J* = 3.1 Hz, 1 H), 6.51 (d, *J* = 2.7 Hz, 1 H), 6.80 (dd, *J* = 8.5, 2.7 Hz, 1 H), 7.18 (d, *J* = 8.5 Hz, 1 H), 7.31 (ddd, *J* = 7.8, 4.8, 0.9 Hz, 1 H), 7.68 (dt, *J* = 7.8, 2.0 Hz, 1 H), 8.58 (dd, *J* = 4.8, 1.7 Hz, 1 H), 8.63 (d, *J* = 1.9 Hz, 1 H). ^13^C NMR (75 MHz, CDCl_3_) *δ* 18.4, 18.5, 21.4, 34.6, 36.3, 37.4, 45.9, 55.4, 71.6, 112.2, 113.0, 123.2, 123.3, 130.7, 134.2, 136.2, 136.6, 136.7, 141.9, 148.5, 149.7, 157.7. IR (ATR) 3271 (br.), 2926, 1229, 1038, 808, 717 cm^−1^. HRMS *m/z*: calcd. for C_23_H_28_NO_2_ 350.2120 [M + H]^+^, found 350.2120.

### Methyl 12-acetyl-7-[[(trifluoromethyl)sulfonyl]oxy]-abieta-6,8,11,13-tetraen-18-oate (19)

Following the procedure for compound **11**, compound **19** was prepared from **6** (1.00 g, 2.70 mmol), Tf_2_O (549 μL, 3.27 mmol) and DTBMP (0.776 g, 3.78 mmol) in dichloromethane (30 mL). Compound **19**: yellowish oil (1.02 g, 75%). ^1^H NMR (300 MHz, CDCl_3_) *δ* 1.17 (s, 3 H), 1.23 (m, 6 H), 1.43 (s, 3 H), 1.80 (m, 6 H), 2.19 (m, 1 H), 2.56 (s, 3 H), 3.05 (d, *J = *2.9 Hz, 1 H), 3.37 (sept, *J* = 7.1 Hz, 1 H), 3.69 (s, 3 H), 5.86 (d, *J* = 2.9 Hz, 1 H), 7.23 (s, 1 H), 7.39 (s, 1 H).^13^C NMR (75 MHz, CDCl_3_) *δ* 18.1, 18.4, 21.0, 23.8, 24.4, 29.4, 31.0, 35.2, 35.6, 37.8, 46.2, 46.5, 52.5, 116.7, 120.3, 120.9, 121.4, 121.9, 129.7, 140.5, 145.1, 145.2, 145.9, 177.6, 203.4. IR (ATR) 2951, 1726, 1688, 1421, 1207, 1140, 964 cm^−1^. HRMS *m/z*: calcd. for C_24_H_30_O_6_SF_3_ 503.1715 [M + H]^+^, found 503.1717.

### Methyl 12-acetyl-7-pyridyl-abieta-6,8,11,13-tetraen-18-oate (20)

Following the procedure for compound **12**, compound **20** was prepared from **19** (0.252 g, 0.501 mmol), (Ph_3_P)_2_PdCl_2_ (24.6 mg, 0.0351 mmol), diethyl(3-pyridyl)borane (111 mg, 0.752 mmol) and 0.8 M Na_2_CO_3_ in H_2_O (2.4 mL, 1.93 mmol) in THF (7 mL). Compound **20**: yellowish solid (132 mg, 61%). ^1^H NMR (400 MHz, CDCl_3_) *δ* 1.04 (d, *J* = 6.8 Hz, 3 H), 1.14 (d, *J* = 6.8 Hz, 3 H), 1.19 (s, 3 H), 1.42 (s, 3 H), 1.81 (m, 5 H), 2.26 (d, *J* = 11.9 Hz, 1 H), 2.59 (s, 1 H), 2.99 (d, *J* = 3.1 Hz, 1 H), 3.38 (sept, *J* = 6.8 Hz, 1 H), 3.72 (s, 2 H), 5.97 (d, *J* = 3.1 Hz, 1 H), 7.00 (s, 1 H), 7.33 (s, 1 H), 7.36 (dd, *J* = 8.0, 5.0 Hz, 1 H), 7.67 (dt, *J* = 7.9, 2.0 Hz, 1 H), 8.62 (m, 2 H). ^13^C NMR (75 MHz, CDCl_3_) *δ* 18.2, 18.4, 21.2, 24.0, 24.3, 29.2, 30.9, 35.7, 35.8, 37.4, 46.2, 47.0, 52.3, 121.7, 123.5, 124.1, 132.8, 135.1, 135.9, 136.1, 136.5, 138.8, 145.5, 145.8, 148.1, 148.9, 178.2, 203.5. IR (ATR) 2947, 1724, 1682, 1243, 1138, 716 cm^−1^. HRMS *m/z*: calcd. for C_28_H_34_ NO_3_ 432.2539 [M + H]^+^, found 432.2541.

### 12-Acetyl-7-pyridyl-abieta-6,8,11,13-tetraen-18-oic acid (21)

Following the procedure for compound **14**, compound **21** was prepared from **20** (0.450 g, 1.04 mmol) and KOH (176 mg, 3.13 mmol) in a mixture of ethylene glycol/water 10:1 (6.6 mL). Compound **21**: yellowish solid (306 mg, 70%). ^1^H NMR (300 MHz, CDCl_3_) *δ* 0.98 (d, *J* = 6.8 Hz, 3 H), 1.12 (d, *J* = 6.8 Hz, 3 H), 1.21 (s, 3 H), 1.51 (s, 3 H), 1.84 (m, 6 H), 2.26 (d, *J* = 11.9 Hz, 1 H), 2.58 (s, 3 H), 3.08 (d, *J* = 3.0 Hz, 2 H), 3.38 (sept, *J* = 6.7 Hz, 1 H), 6.28 (d, *J* = 3.1 Hz, 1 H), 6.92 (s, 1 H), 7.35 (s, 1 H), 7.40 (dd, *J = *8.0, 5.0 Hz, 1 H), 7.72 (dt, *J* = 7.9, 1.9 Hz, 1 H), 8.56 (dd, *J* = 5.0, 1.6 Hz, 1 H), 8.85 (dd, *J* = 2.2, 0.8 Hz, 1 H). ^13^C NMR (75 MHz, CDCl_3_) *δ* 18.5, 18.6, 21.2, 23.9, 24.4, 29.1, 30.8, 35.8, 36.1, 37.3, 45.7, 47.1, 121.9, 123.3, 123.8, 134.7, 135.0, 135.1, 137.1, 137.4, 138.5, 145.5, 146.1, 146.4, 148.1, 181.0, 203.5. IR (ATR) 2930, 2488 (br.), 1911 (br.), 1682, 1238, 812, 714 cm^−1^. HRMS *m/z*: calcd. for C_27_H_32_NO_3_ 418.2382 [M + H]^+^, found 418.2386.

### 12-Acetyl-7-pyridyl-abieta-6,8,11,13-tetraen-18-amide (22)

Following the procedure for compound **15**, compound **22** was prepared from **21** (0.150 g, 0.358 mmol), EDC hydrochloride (75.3 mg, 0.393 mmol), HOBt monohydrate (53.1 mg, 0.393 mmol) and a 25% aqueous solution of ammonia (138 μL, 0.89 mmol) in DMF (2 mL). Compound **22**: white solid (75 mg, 50%). ^1^H NMR (300 MHz, CDCl_3_) *δ* 1.03 (d, *J* = 6.8 Hz, 3 H), 1.14 (d, *J* = 6.8 Hz, 3 H), 1.21 (s, 3 H), 1.45 (s, 3 H), 1.85 (m, 6 H), 2.28 (d, *J* = 10.4 Hz, 1 H), 2.58 (s, 3 H), 2.96 (d, *J = *3.2 Hz, 1 H), 3.36 (sept, *J* = 6.9 Hz, 1 H), 5.68 (d, *J* = 66.4 Hz, 2 H), 6.01 (d, *J* = 3.1 Hz, 1 H), 6.99 (s, 1 H), 7.33 (m, 2 H), 7.66 (dt, *J* = 7.8, 2.0 Hz, 1 H), 8.61 (m, 2 H). ^13^C NMR (75 MHz, CDCl_3_) *δ* 18.4, 18.6, 21.2, 23.9, 24.4, 29.3, 30.9, 35.7, 36.9, 37.5, 46.1, 47.2, 121.6, 123.4, 124.2, 132.2, 135.2, 135.8, 136.2, 136.6, 138.8, 145.5, 145.8, 148.8, 149.5, 180.1, 203.5. IR (ATR) 3341, 3182, 2928, 1666, 810, 715 cm^−1^. HRMS *m/z*: calcd. for C_27_H_33_N_2_O_2_ 417.2542 [M + H]^+^, found 417.2542.

### Methyl 12-hydroxyimino-7-pyridyl-abieta-6,8,11,13-tetraen-18-oate (23)

Compound **20** (0.300 g, 0.695 mmol) was dissolved in EtOH (3 mL) followed by the addition of hydroxylamine hydrochloride (77.3 mg, 1.11 mmol) and pyridine (85 μL). The solution was stirred at reflux for 2.5 h after which solvents were evaporated and the resulting crude mixture was purified by flash chromatography on silica gel using ethyl acetate in *n*-hexane (20 to 80%) as the eluent giving compound **23** (150 mg, 48%) as a white solid. ^1^H NMR (300 MHz, CDCl_3_) *δ* 1.03 (d, *J* = 6.9 Hz, 3 H), 1.12 (d, *J* = 6.8 Hz, 3 H), 1.17 (s, 3 H), 1.41 (s, 3 H), 1.80 (m, 6 H), 2.23 (m, 4 H), 2.99 (m, 2 H), 3.70 (s, 3 H), 5.88 (d, *J* = 3.1 Hz, 1 H), 6.92 (s, 1 H), 7.03 (s, 1 H), 7.32 (ddd, *J* = 7.9, 4.9, 0.8 Hz, 1 H), 7.66 (dt, *J* = 7.9, 1.9 Hz, 1 H), 8.61 (m, 3 H). ^13^C NMR (75 MHz, CDCl_3_) *δ* 16.9, 18.2, 18.4, 21.2, 24.2, 24.4, 29.9, 35.7, 35.8, 37.4, 46.3, 47.0, 52.3, 122.2, 123.3, 123.5, 131.2, 133.2, 136.2, 136.4, 136.6, 144.3, 146.1, 148.6, 149.6, 158.4, 178.4. IR (ATR) 2956, 1724, 1238, 1137, 922, 752, 714 cm^−1^. HRMS *m/z*: calcd. for C_28_H_35_N_2_O_3_ 447.2648 [M + H]^+^, found 447.2648.

### Methyl 12-methoxyimino-7-pyridyl-abieta-8,11,13-trien-18-oate (24)

Following the procedure for compound **23**, compound **24** was prepared from **20** (0.250 g, 0.58 mmol), methoxyamine hydrochloride (77.4 mg, 0.927 mmol) and pyridine (71 μL) in EtOH (3.1 mL). Compound **24**: yellowish solid (62 mg, 23%). ^1^H NMR (300 MHz, CDCl_3_) *δ* 1.04 (d, *J* = 6.9 Hz, 3 H), 1.14 (d, *J* = 6.8 Hz, 3 H), 1.17 (s, 3 H), 1.41 (s, 3 H), 1.81 (m, 6 H), 2.18 (s, 3 H), 2.21 (m, 1 H), 2.98 (m, 2 H), 3.69 (s, 3 H), 3.97 (s, 3 H), 5.88 (d, *J* = 3.1 Hz, 1 H), 6.92 (s, 1 H), 7.02 (s, 1 H), 7.32 (dd, *J* = 7.8, 4.7 Hz, 1 H), 7.63 (dt, *J* = 7.8, 1.8 Hz, 1 H), 8.59 (m, 2 H). ^13^C NMR (75 MHz, CDCl_3_) *δ* 17.4, 18.2, 18.4, 21.1, 24.2, 24.3, 30.0, 35.7, 35.8, 37.4, 46.3, 47.0, 52.3, 61.9, 122.2, 123.3, 123.6, 131.1, 133.2, 136.0, 136.1, 136.4, 136.6, 144.2, 146.1, 148.6, 149.6, 157.3, 178.4. IR (ATR) 2934, 1724, 1240, 1138, 1045, 864, 715 cm^−1^. HRMS *m/z*: calcd. for C_28_H_35_N_2_O_3_ 461.2804 [M + H]^+^, found 461.2804.

### Methyl 12-benzyloxyimino-7-pyridyl-abieta-8,11,13-trien-18-oate (25)

Following the procedure for compound **23**, compound **25** was prepared from **20** (0.100 g, 0.23 mmol), *O*-benzylhydroxylamine hydrochloride (59.0 mg, 0.37 mmol) and pyridine (28 μL) in EtOH (1 mL). Compound **25**: white solid (27 mg, 21%). ^1^H NMR (300 MHz, CDCl_3_) *δ* 0.95 (d, *J* = 6.9 Hz, 3 H), 1.04 (d, *J = *6.8 Hz, 3 H), 1.16 (s, 3 H), 1.40 (s, 3 H), 1.78 (m, 6 H), 2.23 (s, 3 H), 2.94 (m, 2 H), 3.70 (s, 3 H), 5.21 (s, 2 H), 5.87 (d, *J* = 3.1 Hz, 1 H), 6.90 (s, 1 H), 7.00 (s, 1 H), 7.36 (m, 5 H), 7.62 (dt, *J* = 7.9, 1.9 Hz, 1 H), 8.59 (m, 2 H). ^13^C NMR (75 MHz, CDCl_3_) *δ* 17.7, 18.2, 18.4, 21.1, 24.0, 24.3, 29.8, 35.7, 35.8, 37.4, 46.3, 47.0, 52.2, 76.0, 122.2, 123.2, 123.6, 127.8, 128.2, 128.4, 131.1, 133.2, 136.0, 136.1, 136.5, 136.5, 138.6, 144.3, 146.0, 148.7, 149.7, 157.8, 178.3. IR (ATR) 2928, 1724, 1238, 1138, 1024, 914, 858, 735, 716, 698 cm^−1^. HRMS *m/z*: calcd. for C_35_H_41_N_2_O_3_ 537.3117 [M + H]^+^, found 537.3117.

### Methyl 12-hydroxyimino-abieta-8,11,13-trien-18-oate (26)

Following the procedure for compound **23**, compound **26** was prepared from **7** (0.200 g, 0.561 mmol), hydroxylamine hydrochloride (62.4 mg, 0.898 mmol) and pyridine (69 μL) in EtOH (2.4 mL). Compound **26**: white solid (168 mg, 81%). ^1^H NMR (400 MHz, CDCl_3_) *δ* 1.20 (m, 9 H), 1.27 (s, 3 H), 1.42 (m, 1 H), 1.51 (td, *J* = 12.6, 4.1 Hz, 1 H), 1.76 (m, 5 H), 2.19 (s, 3 H), 2.22 (dd, *J* = 12.6, 2.2 Hz, 1 H), 2.28 (d, *J* = 12.6 Hz, 1 H), 2.89 (m, 2 H), 2.99 (sept, *J* = 6.8 Hz, 1 H), 3.66 (s, 3 H), 6.96 (s, 1 H), 7.01 (s, 1 H). ^13^C NMR (75 MHz, CDCl_3_) *δ* 16.7, 17.1, 18.7, 21.8, 24.4, 24.5, 25.2, 29.7, 29.9, 36.8, 37.1, 38.1, 44.9, 47.8, 52.1, 124.2, 126.3, 134.5, 135.8, 143.6, 147.1, 158.8, 179.2. IR (ATR) 3225, 2930, 1724, 1244, 905, 756 cm^−1^. HRMS *m/z*: calcd. for C_21_H_27_O_3_ 327.2539 [M + H]^+^, found 327.2542.

### Methyl 12-methoxyimino-abieta-8,11,13-trien-18-oate (27)

Following the procedure for compound **23**, compound **27** was prepared from **7** (0.200 g, 0.561 mmol), methoxylamine hydrochloride (61.9 mg, 0.898 mmol) and pyridine (68 μL) in EtOH (2.3 mL). Compound **27**: white solid (88.3 mg, 39%). ^1^H NMR (400 MHz, CDCl_3_) *δ* 1.20 (m, 9 H), 1.27 (s, 3 H), 1.41 (m, 1 H), 1.52 (td, *J* = 12.5, 4.2 Hz, 1 H), 1.74 (m, 5 H), 2.14 (s, 3 H), 2.21 (dd, *J* = 12.6, 2.3 Hz, 1 H), 2.28 (d, *J* = 12.6 Hz, 1 H), 2.88 (dd, *J* = 9.1, 4.7 Hz, 2 H), 3.01 (sept, *J* = 6.9 Hz, 1 H), 3.65 (s, 3 H), 3.94 (s, 3 H), 6.96 (s, 1 H), 7.00 (s, 1 H).^13^C NMR (101 MHz, CDCl_3_) *δ* 16.6, 17.5, 18.6, 21.7, 24.3, 24.4, 25.1, 29.7, 29.8, 36.7, 37.0, 38.0, 44.8, 47.7, 52.0, 61.7, 124.0, 126.3, 134.4, 135.7, 143.5, 146.9, 157.5, 179.1. IR (ATR) 2928, 1720, 1437, 1246, 1049, 862 cm^−1^. HRMS *m/z*: calculated for C_24_H_36_NO_3_ 386.2695 [M + H]^+^ found 386.2697.

### Methyl 12-benzyloxyimino-abieta-8,11,13-trien-18-oate (28)

Following the procedure for compound **23**, compound **28** was prepared from **7** (0.150 g, 0.421 mmol), *O*-benzylhydroxylamine hydrochloride (107 mg, 0.673 mmol) and pyridine (53 μL) in EtOH (2 mL). Compound **28**: colourless oil (142 mg, 73%, inseparable mixture of two isomers in a ratio of 0.8:10). ^1^H NMR (400 MHz, CDCl_3_) *δ* 1.11 (m, 6 H), 1.20 (s, 3 H), 1.27 (s, 3 H), 1.41 (m, 1 H), 1.52 (dd, *J* = 12.6, 4.2 Hz, 1 H), 1.74 (m, 5 H), 2.19 (m, 4 H), 2.26 (d, *J* = 12.7 Hz, 1 H), 2.89 (m, 3 H), 3.66 (s, 3 H), 5.19 (s, 2 H), 6.93 (s, 1 H), 6.98 (s, 1 H), 7.36 (m, 5 H). ^13^C NMR (75 MHz, CDCl_3_) *δ* 16.7, 17.9, 18.7, 21.7, 24.3, 24.4, 25.2, 29.6, 29.9, 36.8, 37.1, 38.1, 44.9, 47.8, 52.0, 75.8, 124.1, 126.4, 127.7, 128.2, 128.4, 134.5, 135.7, 138.7, 143.6, 147.0, 158.0, 179.2. IR (ATR) 2930, 1724, 1244, 1109, 1024, 754, 698 cm^−1^. HRMS *m/z*: calcd. for C_23_H_28_NO_2_ 462.3008 [M + H]^+^, found 462.3008.

### Methyl 7,12-di(hydroxyimino)-abieta-8,11,13-trien-18-oate (29)

Following the procedure for compound **23**, compound **29** was prepared from **6** (0.150 g, 0.405 mmol), hydroxylamine hydrochloride (0.0900 g, 1.30 mmol) and pyridine (98 μL) in EtOH (3 mL). Compound **29**: white solid (168 mg, 81%). ^1^H NMR (400 MHz, CDCl_3_) *δ* 1.13 (s, H), 1.25 (dd, *J* = 14.8, 6.8 Hz, 6 H), 1.37 (s, 3 H), 1.71 (m, 5 H), 2.22 (s, 3 H), 2.30 (m, 2 H), 2.65 (m, 2 H) 3.03 (sept, *J* = 6.8 Hz, 1 H), 3.65 (s, 3 H), 7.06 (s, 1 H), 7.81 (s, 1 H), 8.49 (brs, 2 H).^13^C NMR (75 MHz, CDCl_3_) *δ* 16.7, 17.0, 18.2, 23.1, 23.9, 24.3, 24.4, 30.1, 36.8, 37.2, 37.3, 41.8, 46.7, 52.3, 122.0, 123.1, 129.7, 138.1, 144.6, 148.7, 155.4, 158.5, 178.4. IR (ATR) 3175 (br.), 2956, 1724, 1244, 943, 752 cm^−1^. HRMS *m/z*: calcd. for C_23_H_33_N_2_O_4_ 401.2440 [M + H]^+^, found 401.2441.

### Methyl 7,12-di(methoxyimino)-abieta-8,11,13-trien-18-oate (30)

Following the procedure for compound **23**, compound **30** was prepared from **6** (0.324 g, 0.875 mmol), methoxyamine hydrochloride (0.193 g, 2.80 mmol) and pyridine (212 μL) in EtOH (3.6 mL). Compound **30**: white solid (101 mg, 27%). ^1^H NMR (400 MHz, CDCl_3_) *δ* 1.11 (s, 3 H), 1.25 (dd, *J* = 15.5, 6.8 Hz, 6 H), 1.35 (s, 3 H), 1.70 (m, 5 H), 2.15 (s, 3 H), 2.26 (m, 2 H), 2.55 (m, 2 H), 3.02 (sept, *J* = 6.8 Hz, 1 H), 3.64 (s, 3 H), 3.96 (s, 3 H), 4.01 (s, 3 H), 7.02 (s, 1 H), 7.86 (s, 1 H). ^13^C NMR (101 MHz, CDCl_3_) *δ* 16.7, 17.5, 18.2, 23.0, 24.3, 24.3, 24.4, 30.2, 36.6, 37.2, 37.2, 41.7, 46.7, 52.2, 61.9, 62.2, 122.0, 122.9, 129.7, 137.9, 144.4, 148.4, 153.8, 157.5, 178.4. IR (ATR) 2935, 1726, 1242, 1045, 893 cm^−1^. HRMS *m/z*: calculated for C_25_H_37_N_2_O_4_ 429.2753 [M + H]^+^ found 429.2749.

### Methyl 7,12-di(benzyloxyimino)-abieta-8,11,13-trien-18-oate (31)

Following the procedure for compound **23**, compound **31** was prepared from **6** (0.150 g, 0.422 mmol), *O*-benzylhydroxylamine hydrochloride (216 mg, 1.35 mmol) and pyridine (102 μL) in EtOH (1.75 mL). Compound **31**: white solid (105 mg, 45%). ^1^H NMR (400 MHz, CDCl_3_) *δ* 1.09 (s, 3 H), 1.12 (d, *J* = 6.8 Hz, 3 H), 1.18 (d, *J* = 6.8 Hz, 3 H), 1.33 (s, 3 H), 1.69 (m, 5 H), 2.20 (s, 3 H), 2.26 (m, 2 H), 2.59 (m, 2 H), 2.93 (sept, *J* = 6.8 Hz, 1 H), 3.61 (s, 3 H), 5.20 (s, 2 H), 5.24 (s, 2 H), 6.99 (s, 1 H), 7.36 (m, 10 H), 7.83 (s, 1 H). ^13^C NMR (101 MHz, CDCl_3_) *δ* 16.7, 17.8, 18.2, 23.1, 24.2, 24.3, 24.5, 30.0, 36.6, 37.2, 41.8, 46.7, 52.2, 76.0, 76.5, 122.2, 122.9, 127.8, 127.9, 128.2, 128.4, 128.4, 129.8, 137.9, 138.3, 138.5, 144.4, 148.4, 154.2, 157.9, 178.4. IR (ATR) 2928, 1722, 1246, 1013, 906, 731 cm^−1^. HRMS *m/z*: calculated for C_37_H_45_N_2_O_4_ 581.3379 [M + H]^+^ found 581.3381.

### Biology

U937 cells were cultured in RPMI 1640 supplemented with 5% fetal bovine serum (FBS) and 1% penicillin-streptomycin, at 37 °C and 5% CO_2_. RAW 264.7 and PanAsc 2159 cells were grown in DMEM/10% FBS/1% penicillin-streptomycin. Aspc-1 cells were cultured in RPMI 1640 supplemented with 10% FBS and 1% penicillin-streptomycin.

### NO assay

RAW 264.7 cells were plated in 96-well plates 2 × 10^5^ per well. The next day, cells were treated with various concentrations of compounds for 20 min, and then stimulated with 10 ng/mL of interferon-γ for a total of 24 h. Twenty-four hours later NO production was measured in the medium as nitrite using the Griess reaction^45^, and plates were read at 550 nm.

### MTT assay

Aspc-1 or PanAsc 2159 cells were plated in 96-well plates, 2500 cells/well for PanAsc 2159 and 3000 cells/well for Aspc-1. The next day, cells were treated with various concentrations of compounds and incubated for 72 h. Then, MTT solution (thiazolyl blue tetrazolium bromide, M5655, Sigma-Aldrich, St. Louis, MO) at 10 mM in PBS, pH 7.4 was added to each well and cells were incubated for 3 h. The supernatant was removed and a developing solution (0.04 M HCl in isopropanol) was added. Plates were read at 630–570 nm. 100% of cell viability was considered for control cells.

### Flow cytometry

Monocytic differentiation was evaluated in U937 cells, which were treated with the compounds for 4 days and then stained for 30 min with a CD11b-FITC monoclonal antibody from BD Pharmingen. Cells were resuspended in 500 μL PBS/BSA/sodium azide prior to FACS analysis using LSRII-Diva 6.2 (BD). The geometric mean for each sample was determined using FlowJo V10.0 7r2 software (Tree Star).

### Western blot

Aspc-1 or PanAsc 2159 cells were plated 2.5 × 10^5^ or 2.0 × 10^5^ per 100 mm^3^ dish and treated the next day with three different concentrations of the compounds for 48 h. Proteins were extracted with RIPA lysis buffer supplemented with protease inhibitors (1 mM phenylmethylsulfonyl fluoride, 10 μg/mL leupeptin, 50 μg/mL aprotinin), and concentrations were determined using the bicinchoninic acid (BCA) assay (Sigma). 10–15 micrograms of proteins were resolved on 7.5% or 10% SDS-polyacrylamide gels. Goat anti-rabbit lgG HRP (horseradish peroxidase)-conjugated secondary antibody was purchased from Santa Cruz Biotechnology; cyclin D1, p27 and vinculin antibodies were obtained from Cell Signaling Technology. Developing images: GE Detection reagent 1 and 2, Fisher Scientific Supersignal west femto luminol enhancer solution and West Femto Stable Peroxide buffer.

### Cell cycle

Cell cycle was assessed by flow cytometry using a fluorescence-activated cell sorter (FACS-BD-LSRII). PanAsc 2159 and Aspc-1 cells were treated with compounds **1**, **23**, **24**, **25**, **26** and **29** at 5 and 10 µM for 48 hours, fixed in ice-cold 70% ethanol overnight and treated with PI (Biolegend, 10 μg/mL)/RNase (Worthington, 0.1 mg/mL) solution (40 minutes at 37 °C). DNA content was quantified by flow cytometry. Data was analyzed in FlowJo x.10.0.7r2 software (Tree Star).

### Statistical analysis

Results are described as mean ± SD unless otherwise noted on the figure legend. Results were analyzed using one-way ANOVA followed by the Dunnett’s multiple comparisons test (GraphPad Prism 7.0). *, P < 0.05 was considered statistically significant.

### Kinase assay

The kinase screening was performed with a radioactive filter binding assay using ^33^P ATP by the International Centre for Kinase Profiling (ICKP). Detailed description of the protocol is reported elsewhere^46,47^.

## Electronic supplementary material

Supporting Information

## Data Availability

The datasets generated during and/or analysed during the current study are available from the corresponding author on reasonable request.
