# Supplementary material for: Dehydroabietic oximes halt pancreatic cancer cell growth in the G1 phase through induction of p27 and downregulation of cyclin D1
Source: Sci Rep. 2018 Oct 29;8:15923. doi: 10.1038/s41598-018-34131-1 (PMC6206059; doi:10.1038/s41598-018-34131-1)
Supplement: Supplementary file 1 — Supporting Information [file 41598_2018_34131_MOESM1_ESM.pdf]

Dehydroabietic oximes halt pancreatic cancer cell growth in the G1 phase through induction of p27 and downregulation of cyclin D1

Laura E. Kolsi[1], Ana S. Leal[2], Jari Yli-Kauhaluoma[1], Karen T. Liby[2,*], Vânia M. Moreira[1,3,*]

[1]Drug Research Program, Division of Pharmaceutical Chemistry and Technology, Faculty of Pharmacy, University of Helsinki, Viikinkaari 5 E (P.O. Box 56), FI-00014, Helsinki, Finland

[2]Department of Pharmacology and Toxicology, Michigan State University, 1355 Bogue Street, East Lansing, MI 48824, USA

[3]Strathclyde Institute of Pharmacy and Biomedical Sciences, University of Strathclyde, 161 Cathedral Street, Glasgow G4 0RE, UK

*vania.moreira@strath.ac.uk

*libykare@msu.edu

Supplementary information

Table of Contents

NMR spectra……………………………………… S3
^{1}H-NMR spectrum of compound **9** recorded in $CDCl_3$……… S3
^{13}C-NMR spectrum of compound **9** recorded in $CDCl_3$……… S3
^{1}H-NMR spectrum of compound **10** recorded in $CDCl_3$……… S4
^{13}C-NMR spectrum of compound **10** recorded in $CDCl_3$……… S4
^{1}H-NMR spectrum of compound **11** recorded in $CDCl_3$……… S5
^{13}C-NMR spectrum of compound **11** recorded in $CDCl_3$……… S5
^{1}H-NMR spectrum of compound **12** recorded in $CDCl_3$……… S6
^{13}C-NMR spectrum of compound **12** recorded in $CDCl_3$……… S6
^{1}H-NMR spectrum of compound **13** recorded in $CDCl_3$……… S7
^{13}C-NMR spectrum of compound **13** recorded in $CDCl_3$……… S7
^{1}H-NMR spectrum of compound **14** recorded in $CDCl_3$……… S8
^{13}C-NMR spectrum of compound **14** recorded in $CDCl_3$……… S8
^{1}H-NMR spectrum of compound **15** recorded in $CDCl_3$……… S9
^{13}C-NMR spectrum of compound **15** recorded in $CDCl_3$……… S9
^{1}H-NMR spectrum of compound **16** recorded in $CDCl_3$……… S10
^{13}C-NMR spectrum of compound **16** recorded in $CDCl_3$……… S10
^{1}H-NMR spectrum of compound **17** recorded in $CDCl_3$……… S11
^{13}C-NMR spectrum of compound **17** recorded in $CDCl_3$……… S11
^{1}H-NMR spectrum of compound **18** recorded in $CDCl_3$……… S12
^{13}C-NMR spectrum of compound **18** recorded in $CDCl_3$……… S12
^{1}H-NMR spectrum of compound **19** recorded in $CDCl_3$……… S13
^{13}C-NMR spectrum of compound **19** recorded in $CDCl_3$……… S13
^{1}H-NMR spectrum of compound **20** recorded in $CDCl_3$……… S14
^{13}C-NMR spectrum of compound **20** recorded in $CDCl_3$……… S14
^{1}H-NMR spectrum of compound **21** recorded in $CDCl_3$……… S15
^{13}C-NMR spectrum of compound **21** recorded in $CDCl_3$……… S15
^{1}H-NMR spectrum of compound **22** recorded in $CDCl_3$……… S16
^{13}C-NMR spectrum of compound **22** recorded in $CDCl_3$……… S16
^{1}H-NMR spectrum of compound **23** recorded in $CDCl_3$……… S17
^{13}C-NMR spectrum of compound **23** recorded in $CDCl_3$……… S17
^{1}H-NMR spectrum of compound **24** recorded in $CDCl_3$……… S18
^{13}C-NMR spectrum of compound **24** recorded in $CDCl_3$……… S18
^{1}H-NMR spectrum of compound **25** recorded in $CDCl_3$……… S19
^{13}C-NMR spectrum of compound **25** recorded in $CDCl_3$……… S19
^{1}H-NMR spectrum of compound **26** recorded in $CDCl_3$……… S20
^{13}C-NMR spectrum of compound **26** recorded in $CDCl_3$……… S20
^{1}H-NMR spectrum of compound **27** recorded in $CDCl_3$……… S21
^{13}C-NMR spectrum of compound **27** recorded in $CDCl_3$……… S21
^{1}H-NMR spectrum of compound **28** recorded in $CDCl_3$……… S22
^{13}C-NMR spectrum of compound **28** recorded in $CDCl_3$……… S22
^{1}H-NMR spectrum of compound **29** recorded in $CDCl_3$……… S23
^{13}C-NMR spectrum of compound **29** recorded in $CDCl_3$……… S23
^{1}H-NMR spectrum of compound **30** recorded in $CDCl_3$……… S24
^{13}C-NMR spectrum of compound **30** recorded in $CDCl_3$……… S24
^{1}H-NMR spectrum of compound **31** recorded in $CDCl_3$……… S25
^{13}C-NMR spectrum of compound **31** recorded in $CDCl_3$……… S25

Original bands for Western blot studies……………………… S26

Cell cycle analysis data……………………………………… S27

Kinase assay data……………………………………………… S28

NMR spectra

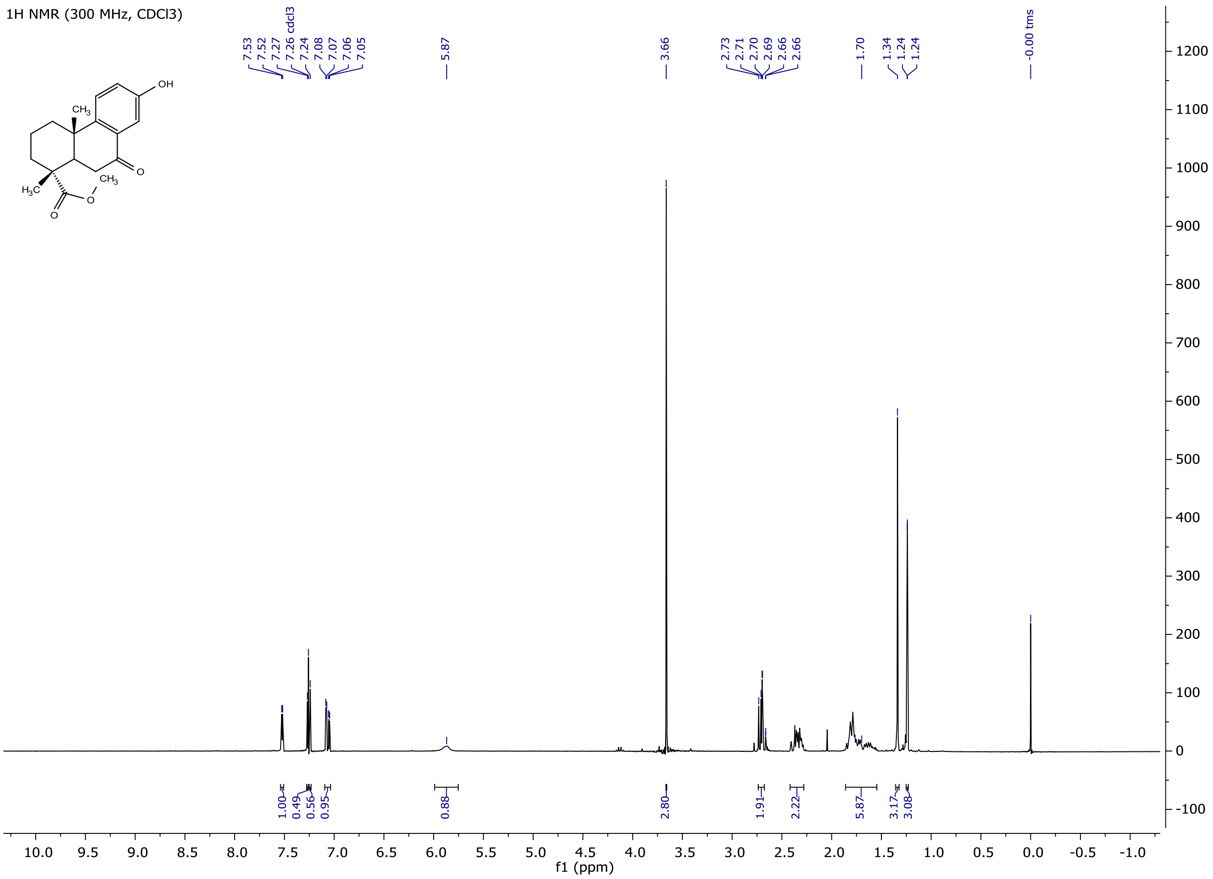

^{1}H-NMR spectrum of compound **9** recorded in $CDCl_3$

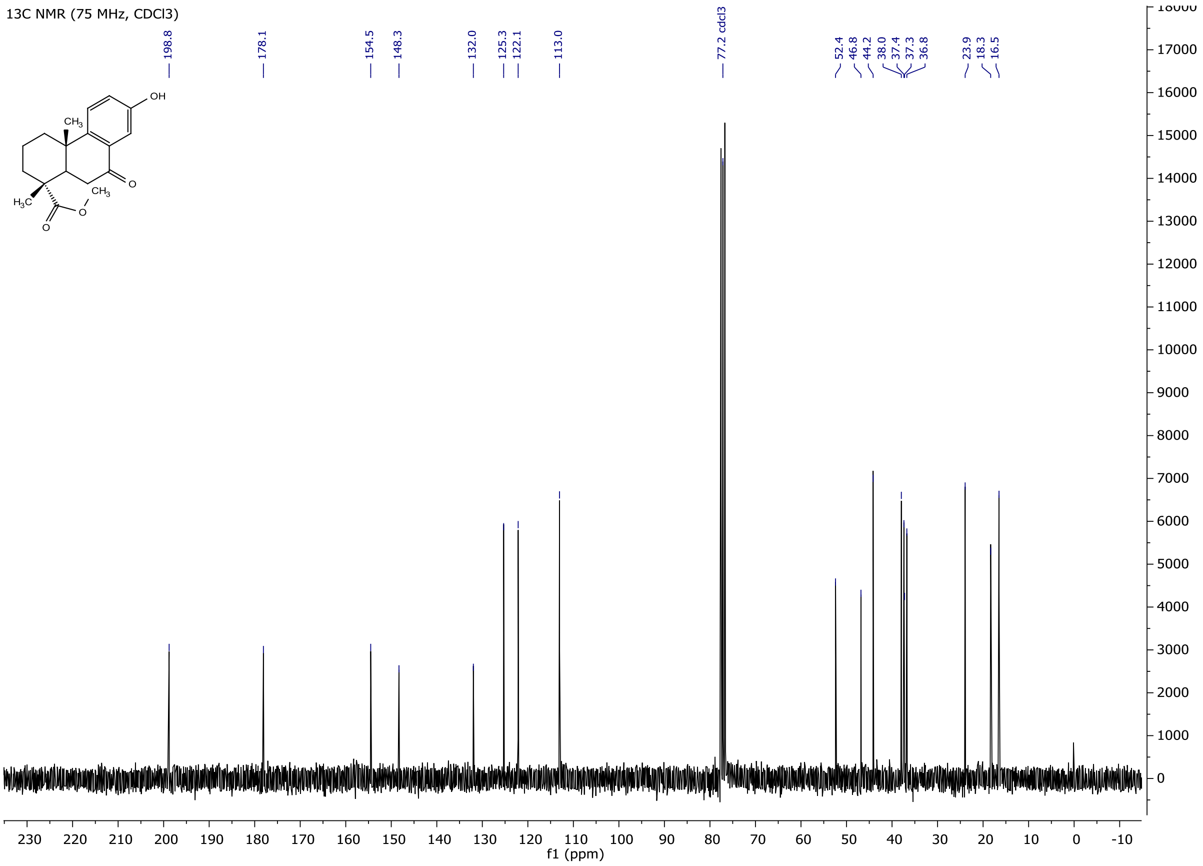

^{13}C-NMR spectrum of compound **9** recorded in $CDCl_3$

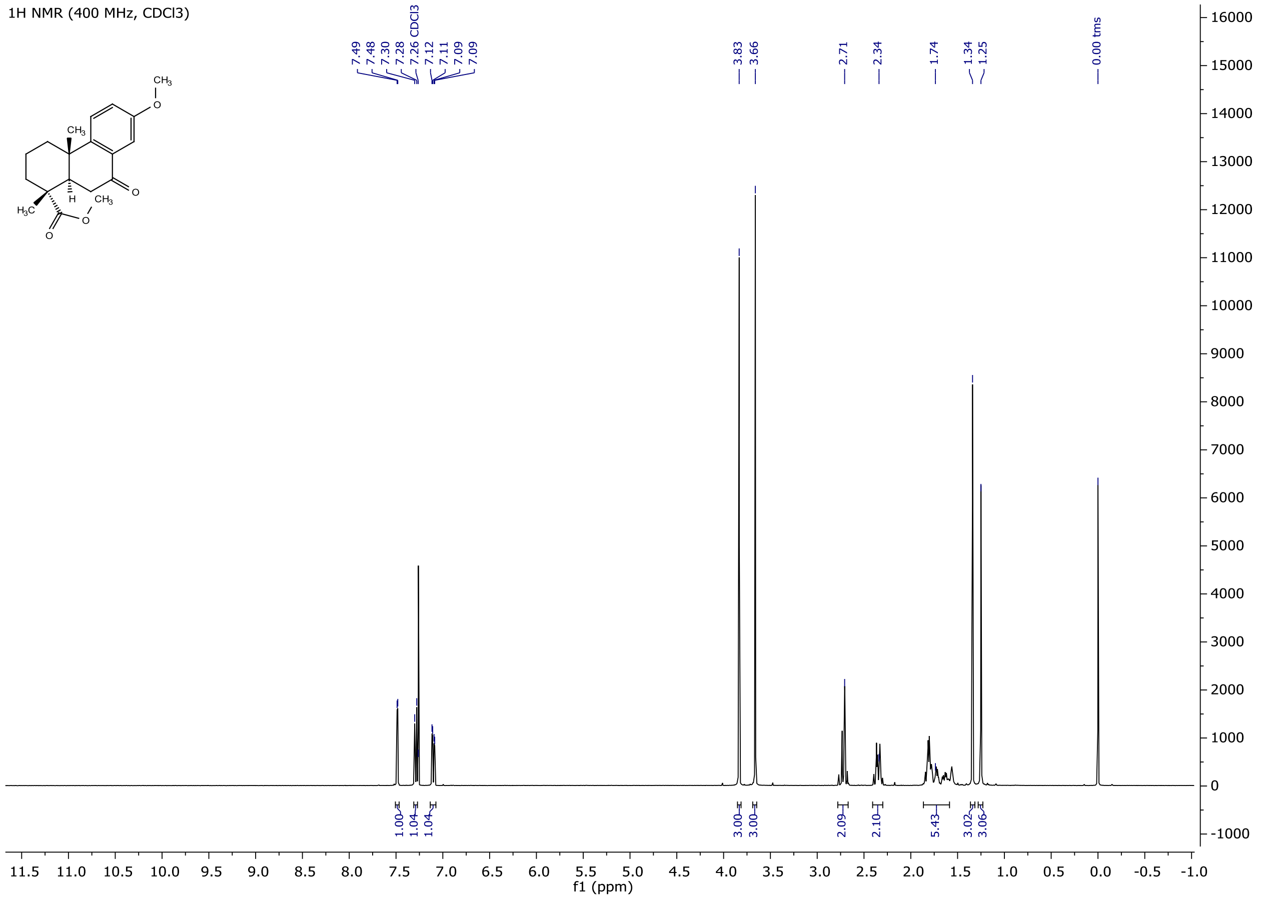

^{1}H-NMR spectrum of compound **10** recorded in $CDCl_3$

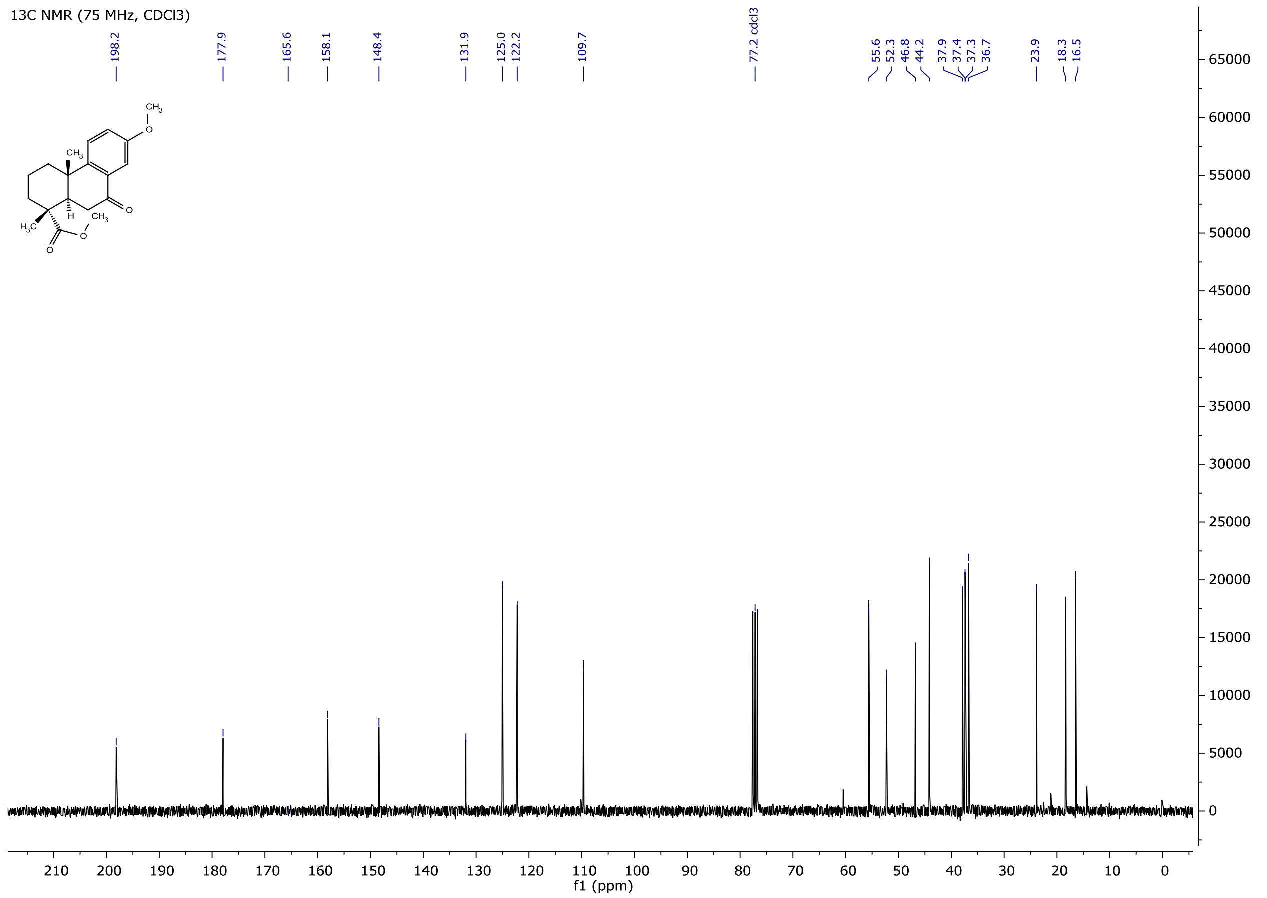

^{13}C-NMR spectrum of compound **10** recorded in $CDCl_3$

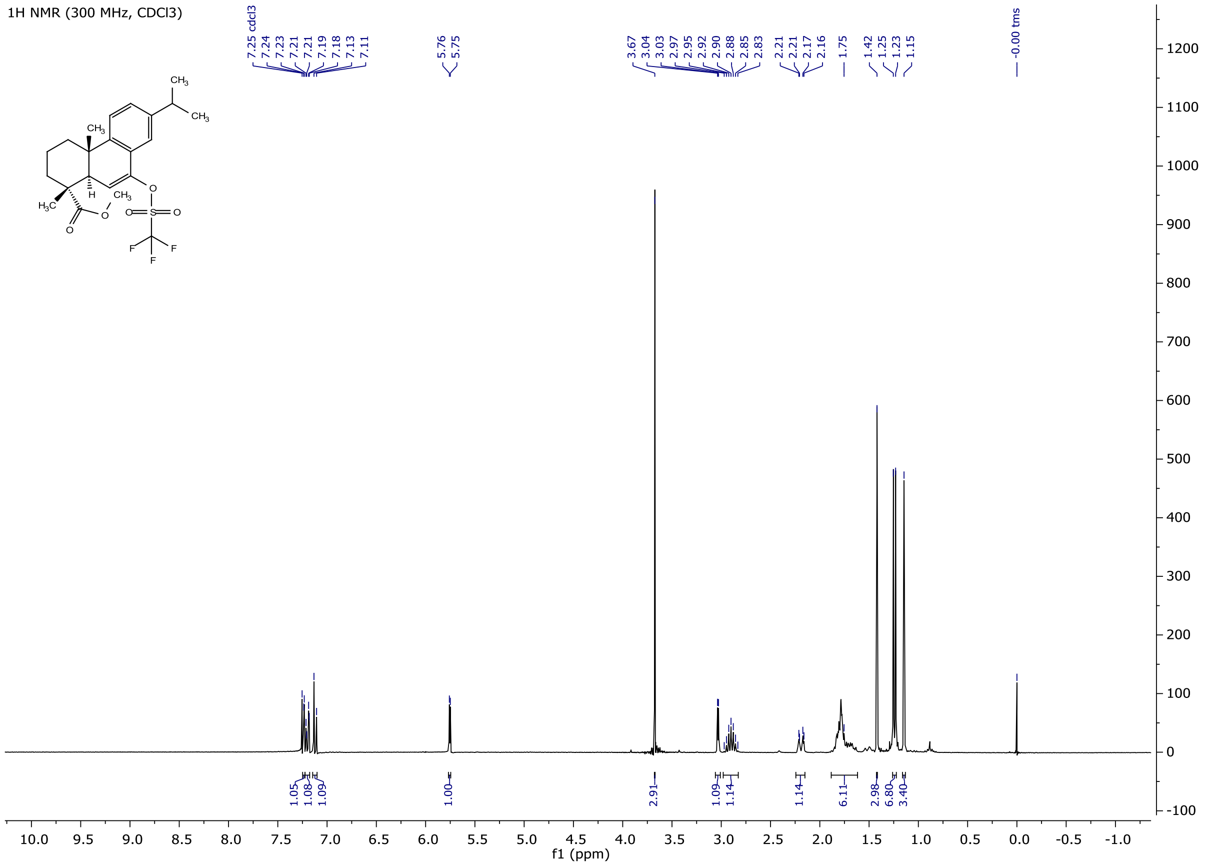

^{1}H-NMR spectrum of compound **11** recorded in $CDCl_3$

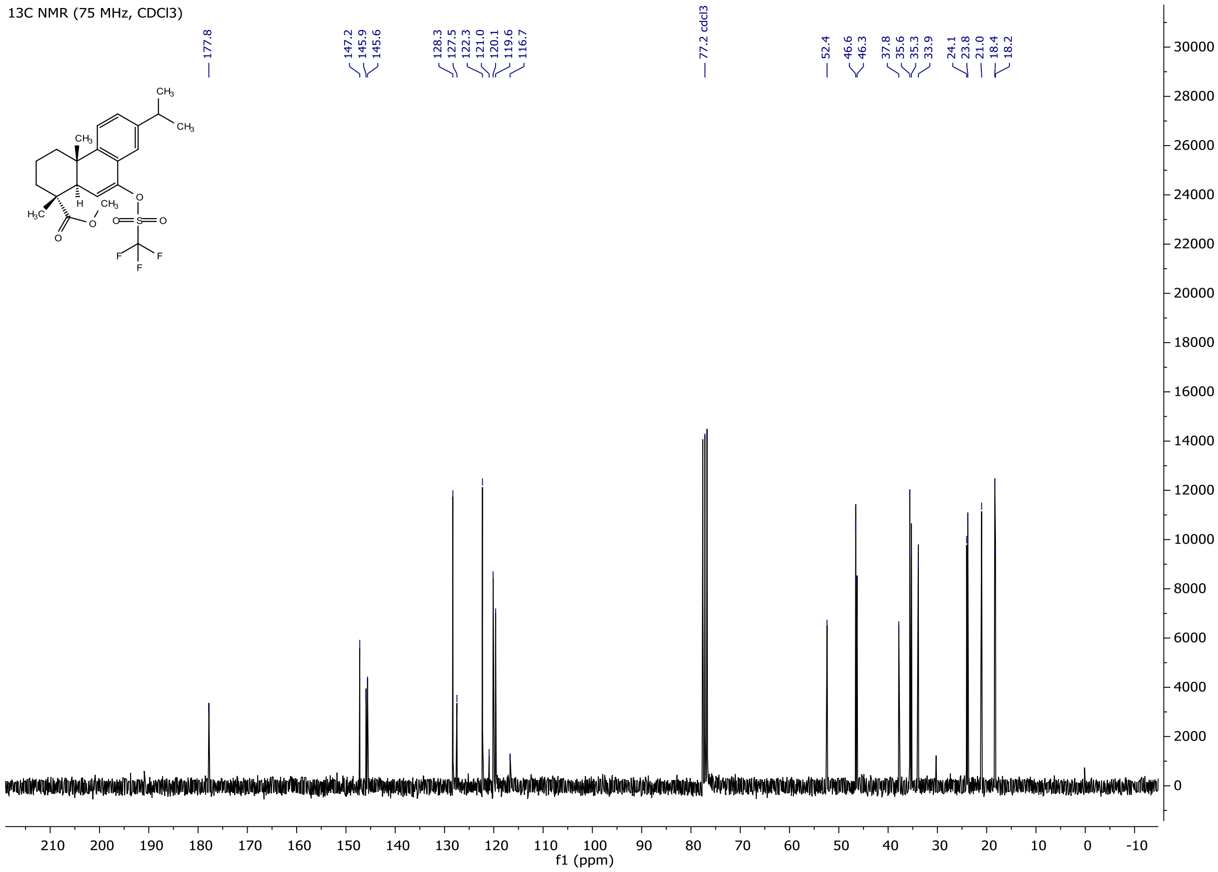

^{13}C-NMR spectrum of compound **11** recorded in $CDCl_3$

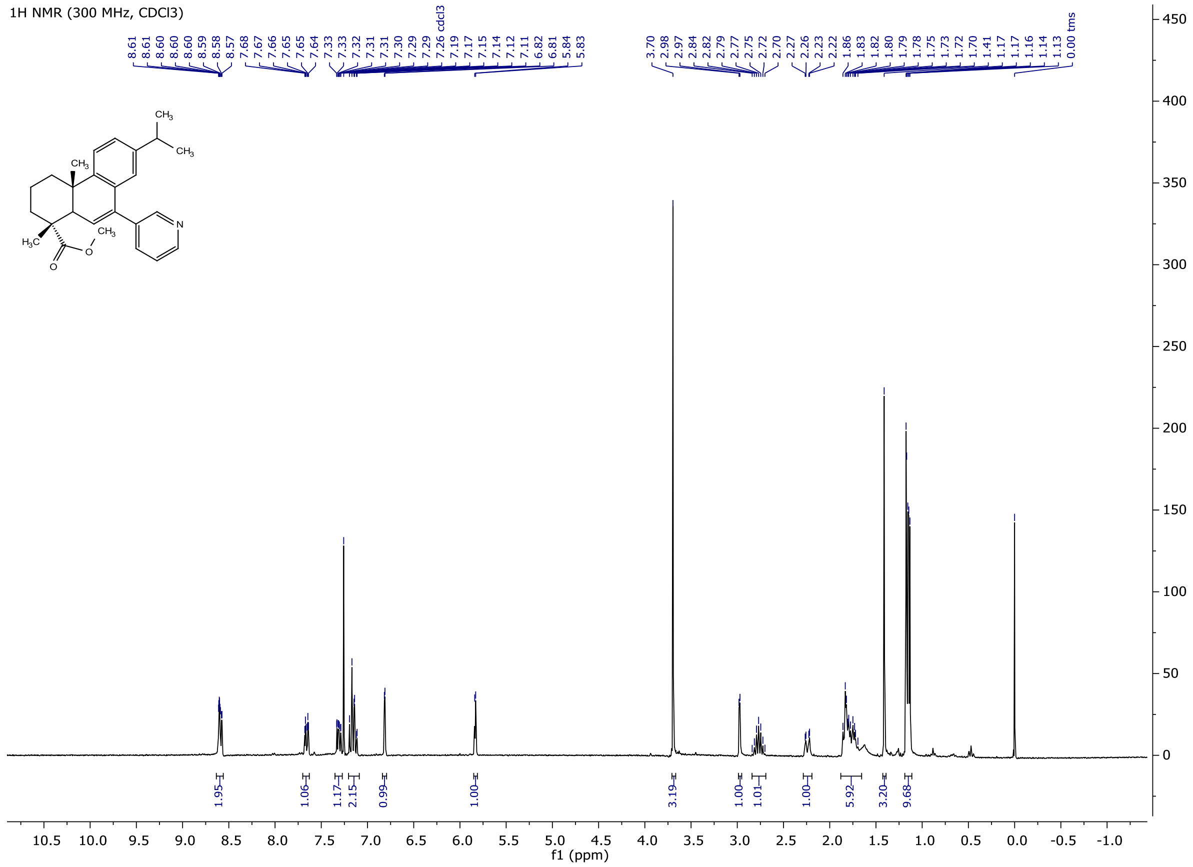

^{1}H-NMR spectrum of compound **12** recorded in $CDCl_3$

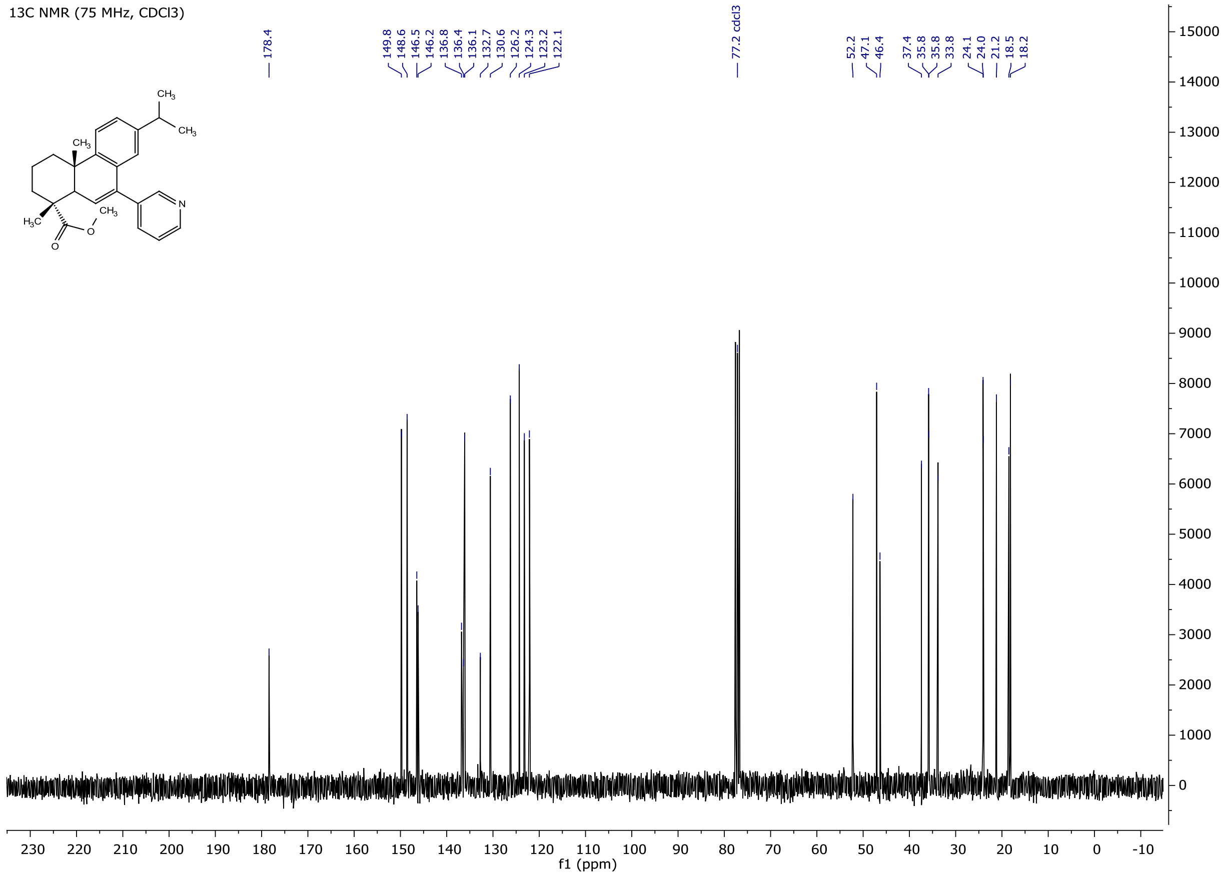

^{13}C-NMR spectrum of compound **12** recorded in $CDCl_3$

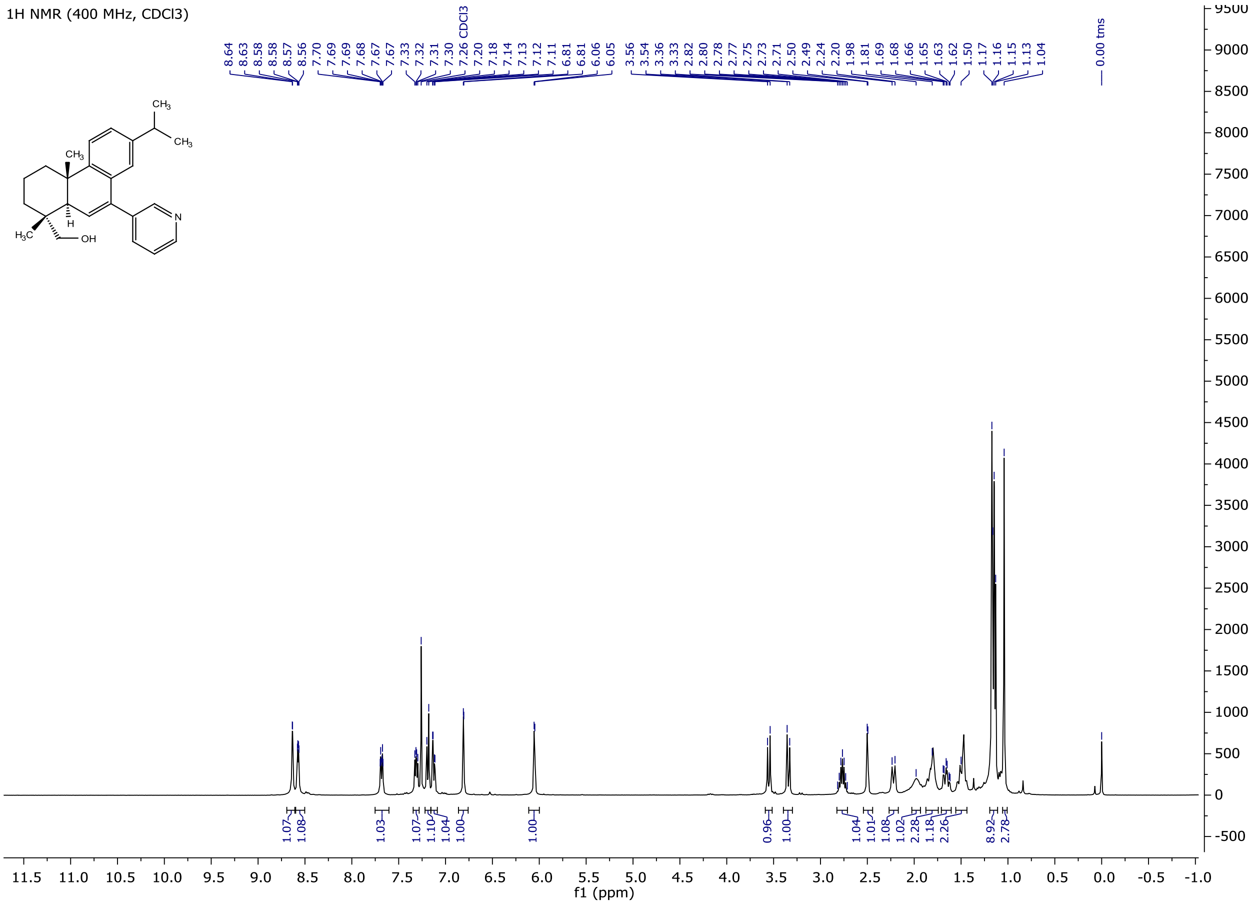

^{1}H-NMR spectrum of compound **13** recorded in $CDCl_3$

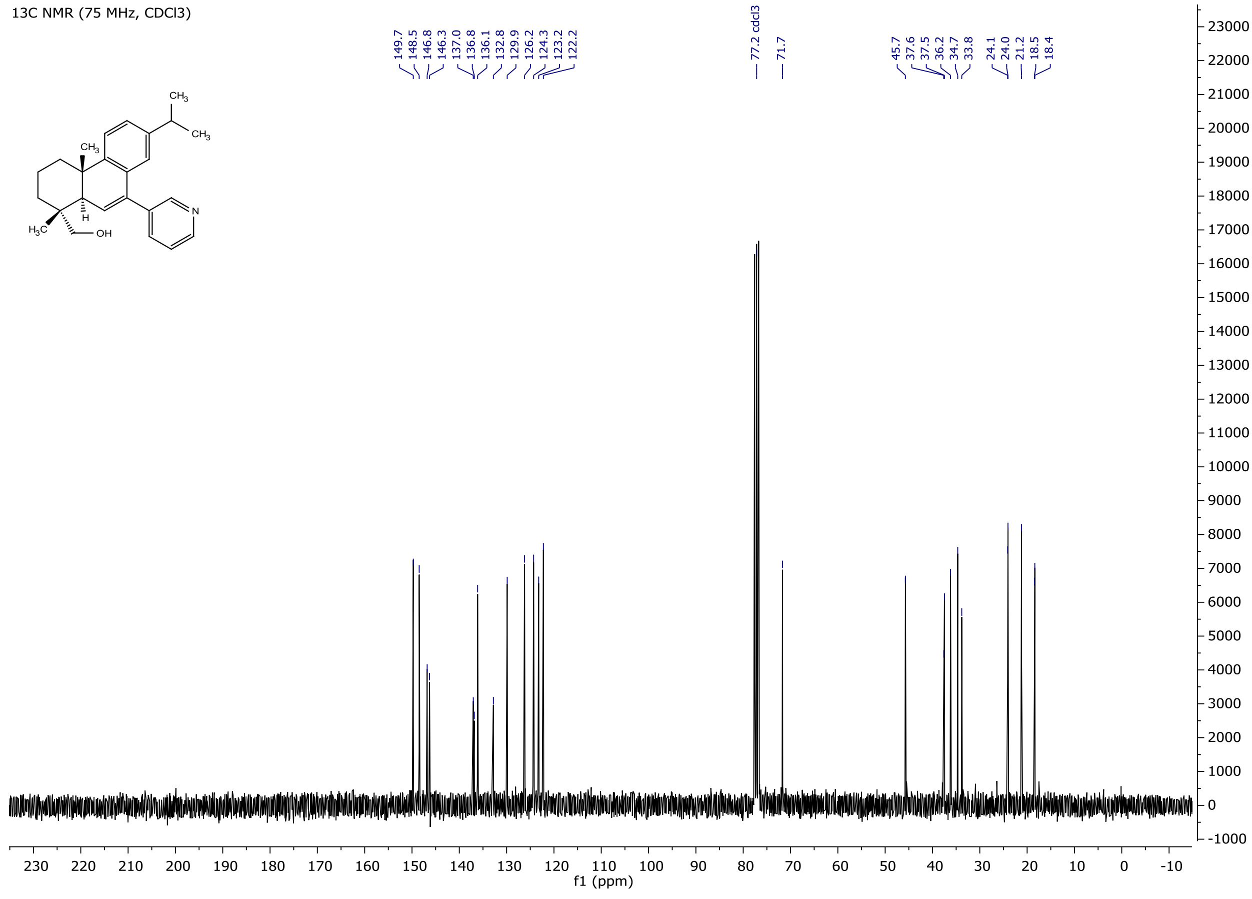

^{13}C-NMR spectrum of compound **13** recorded in $CDCl_3$

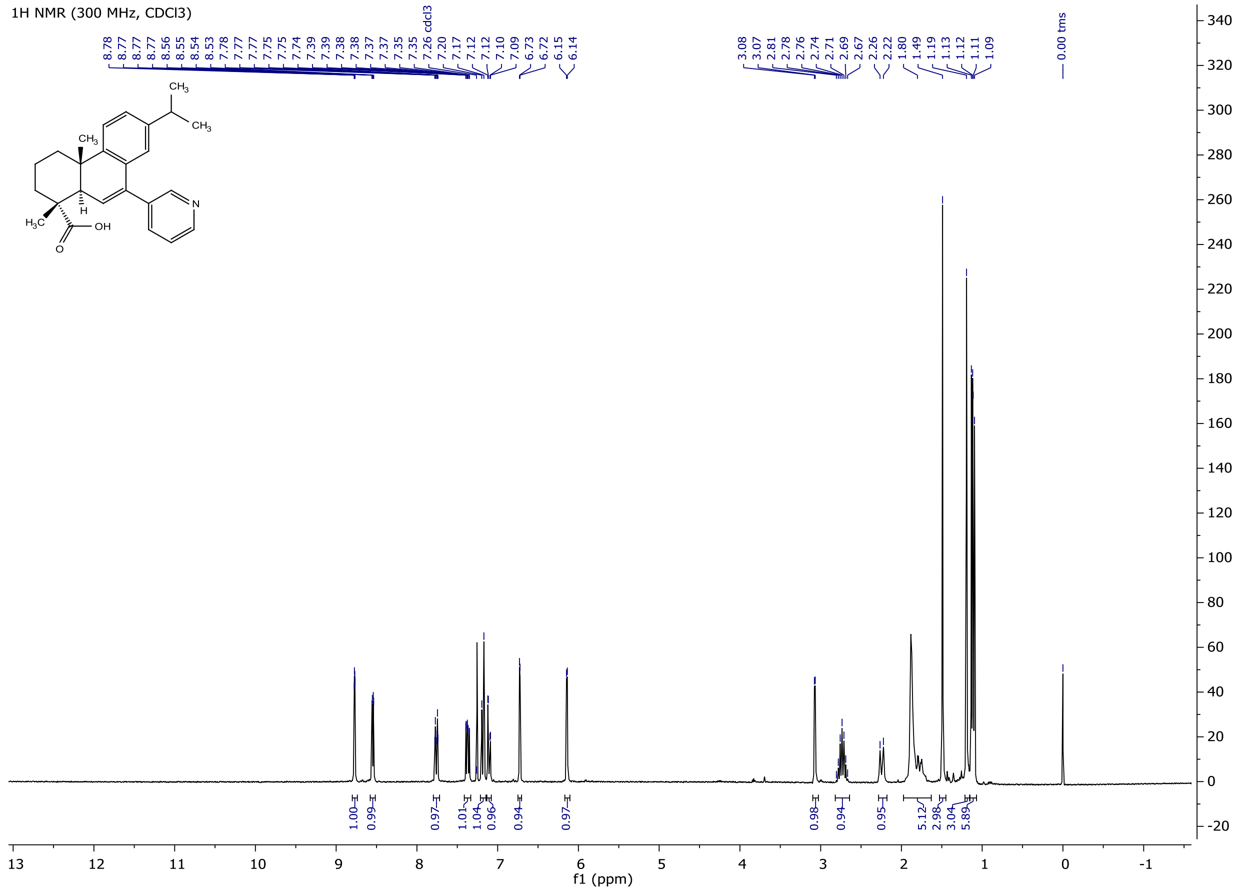

^{1}H-NMR spectrum of compound **14** recorded in $CDCl_3$

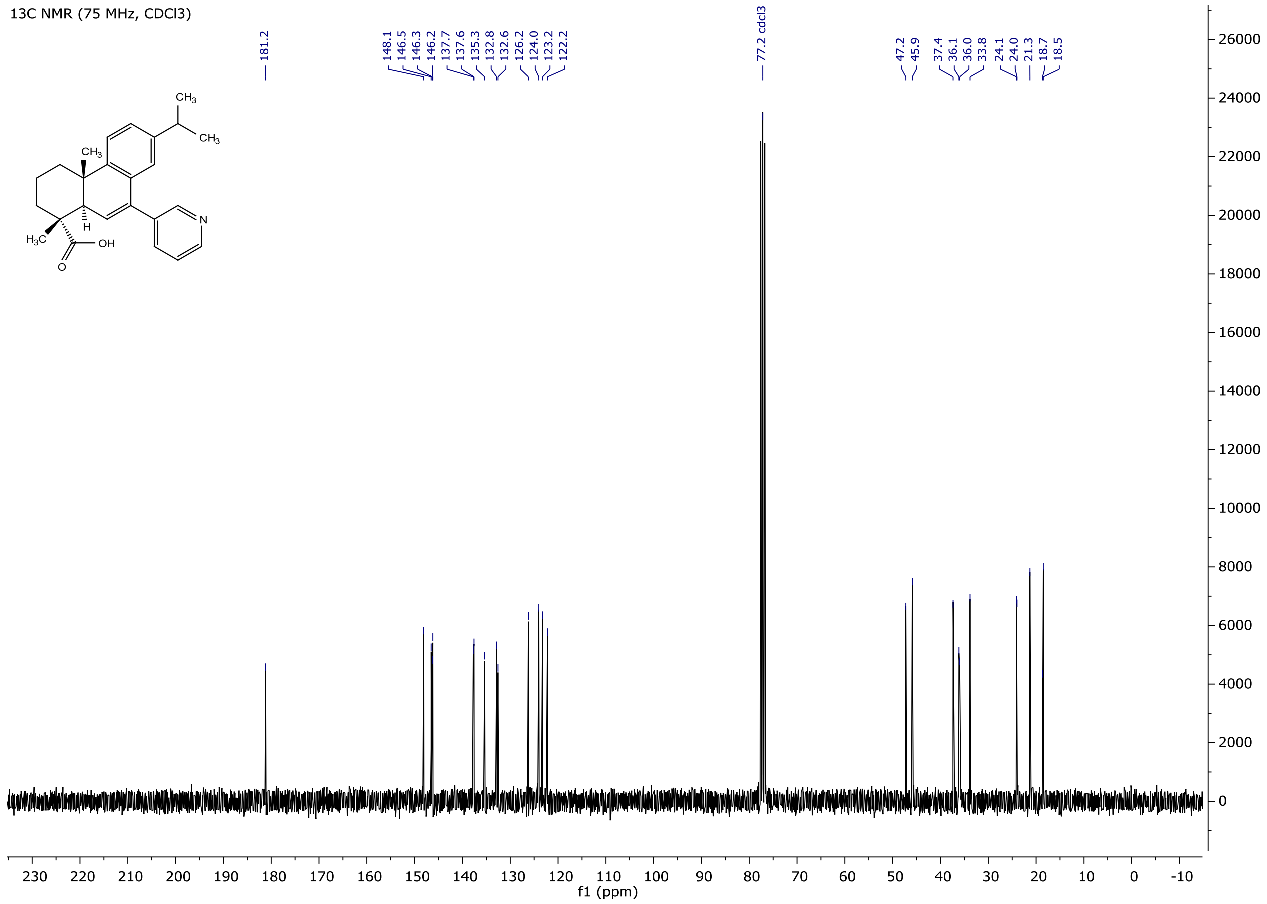

^{13}C-NMR spectrum of compound **14** recorded in $CDCl_3$

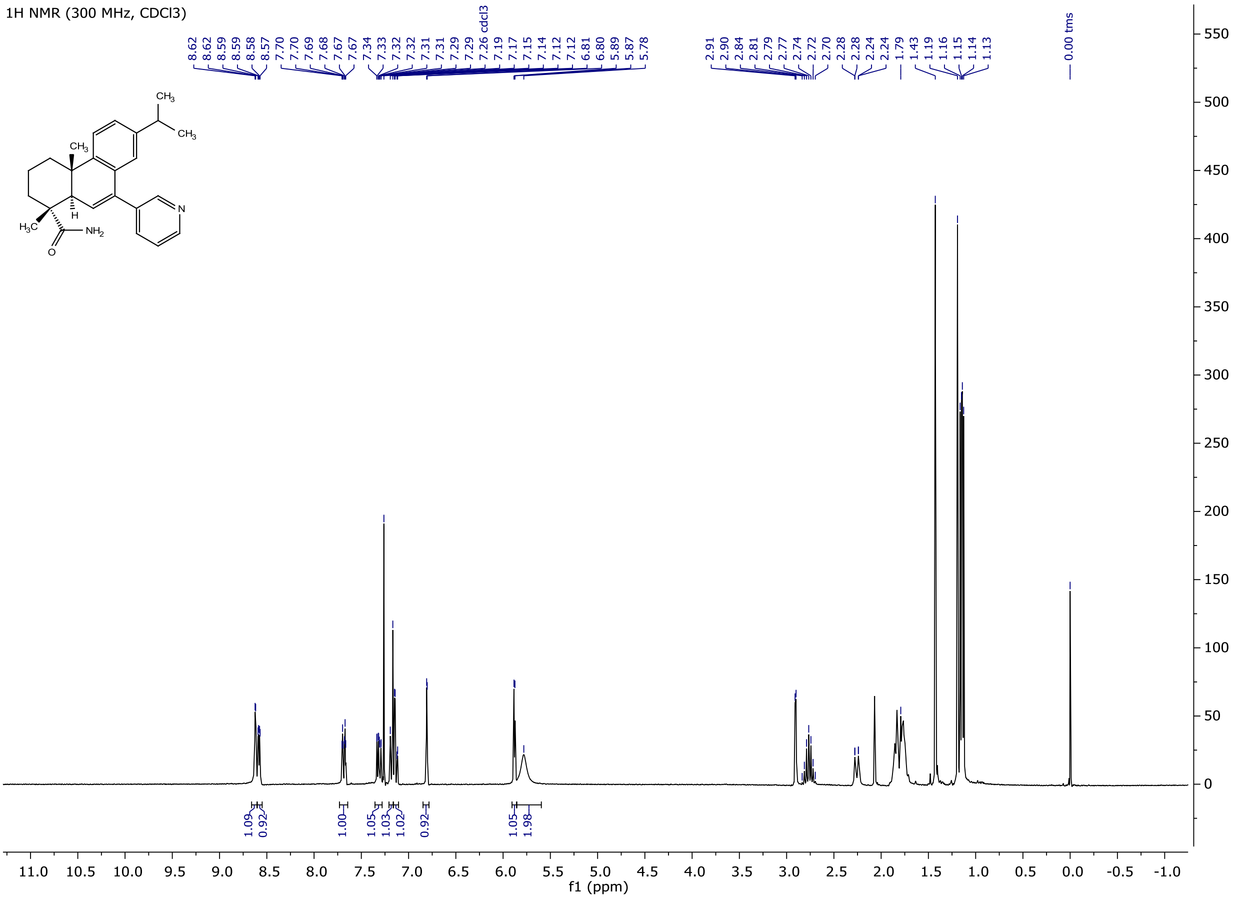

^{1}H-NMR spectrum of compound **15** recorded in $CDCl_3$

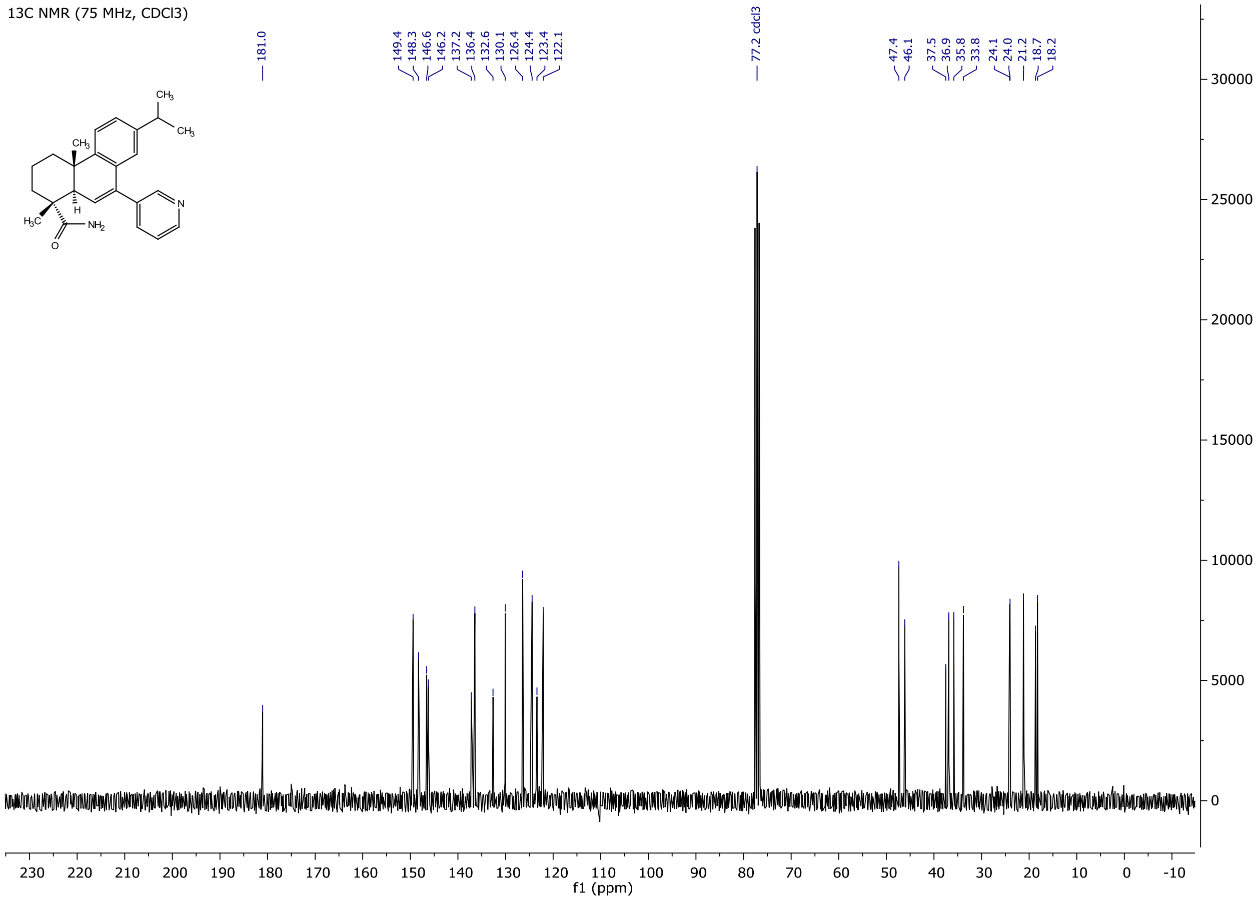

^{13}C-NMR spectrum of compound **15** recorded in $CDCl_3$

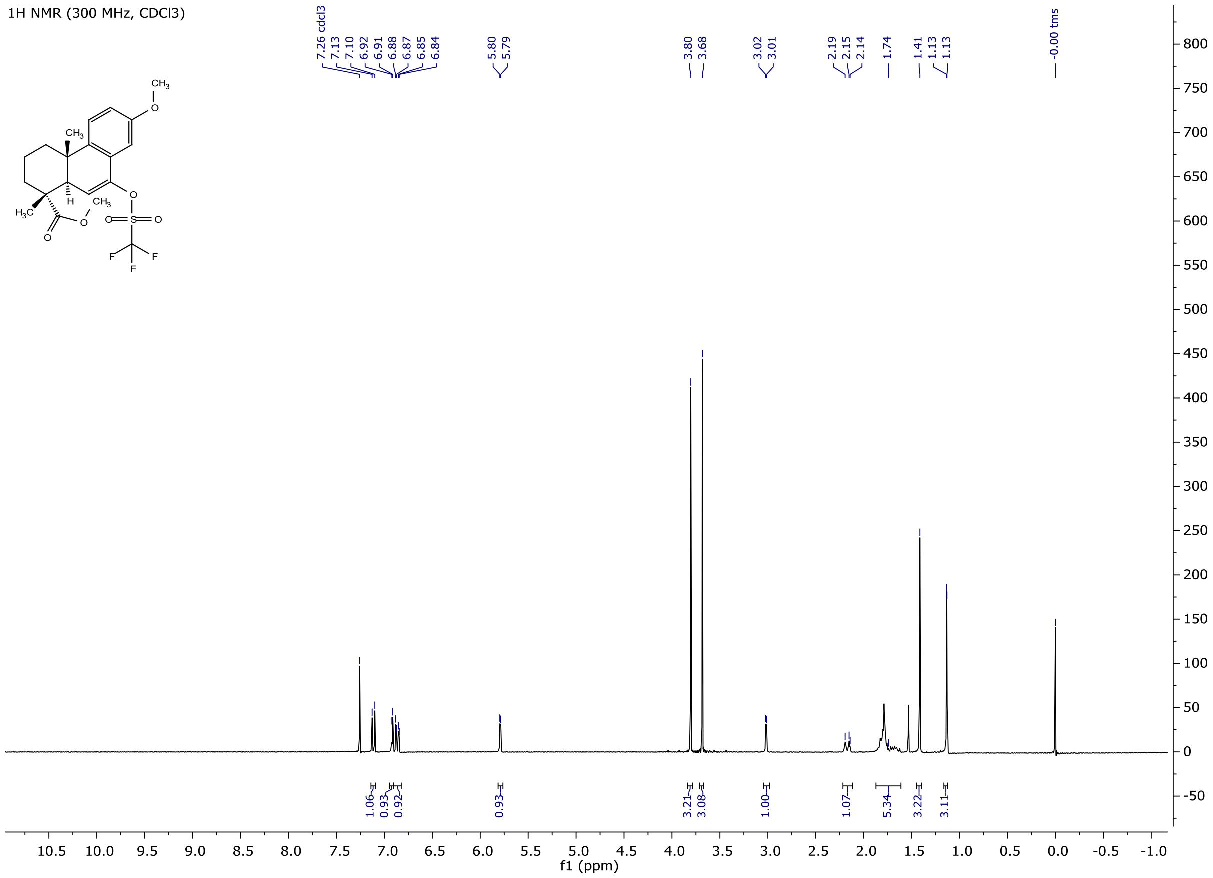

^{1}H-NMR spectrum of compound **16** recorded in $CDCl_3$

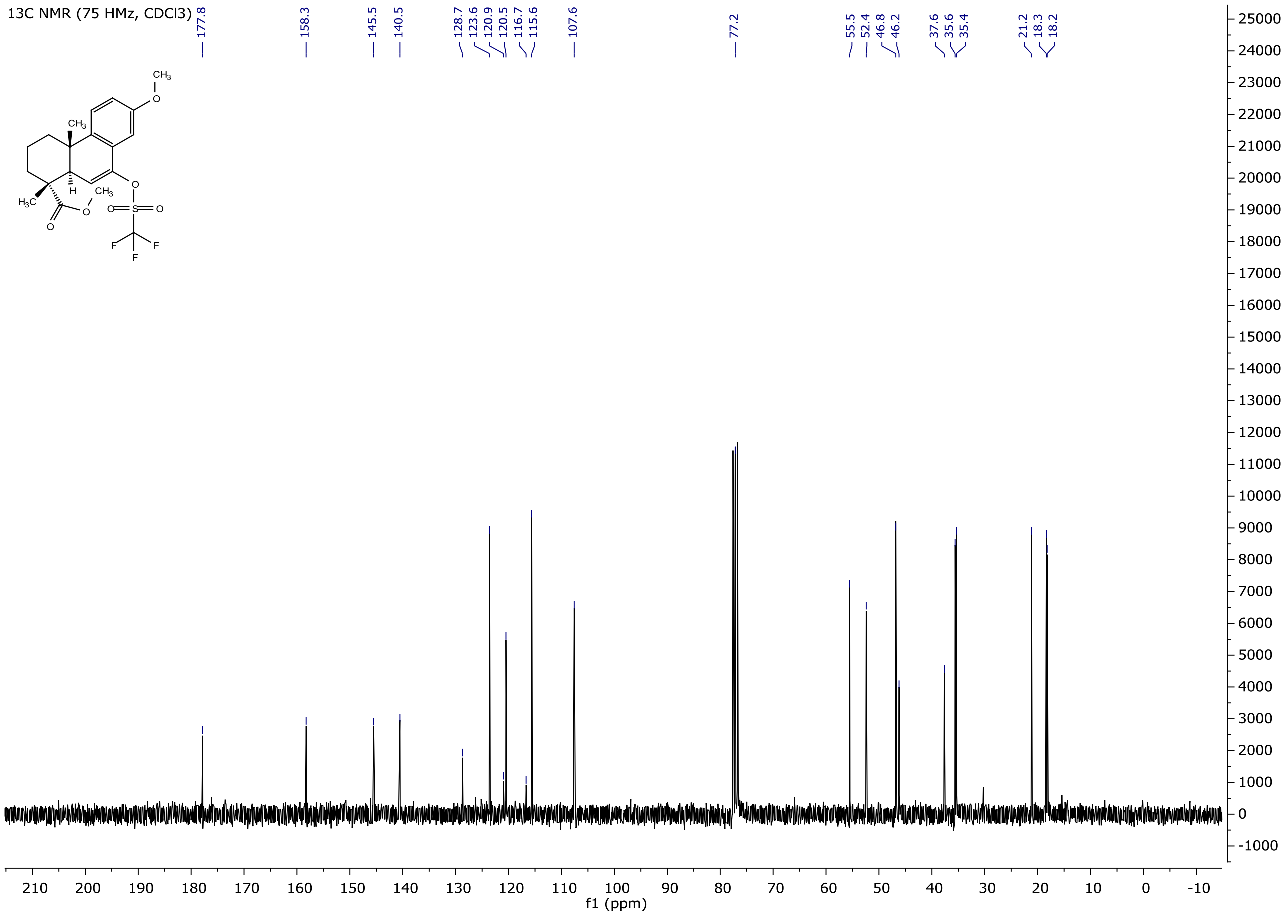

^{13}C-NMR spectrum of compound **16** recorded in $CDCl_3$

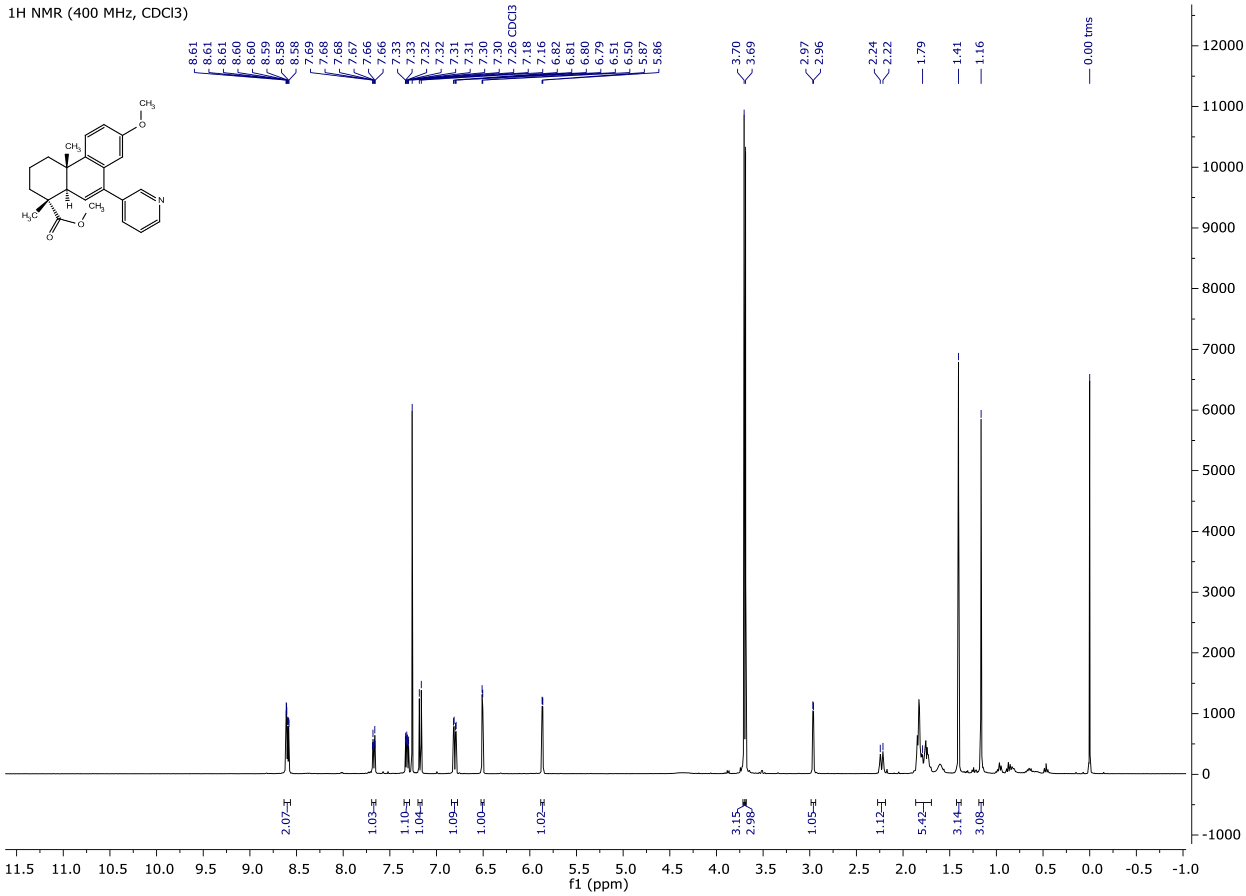

^{1}H-NMR spectrum of compound **17** recorded in $CDCl_3$

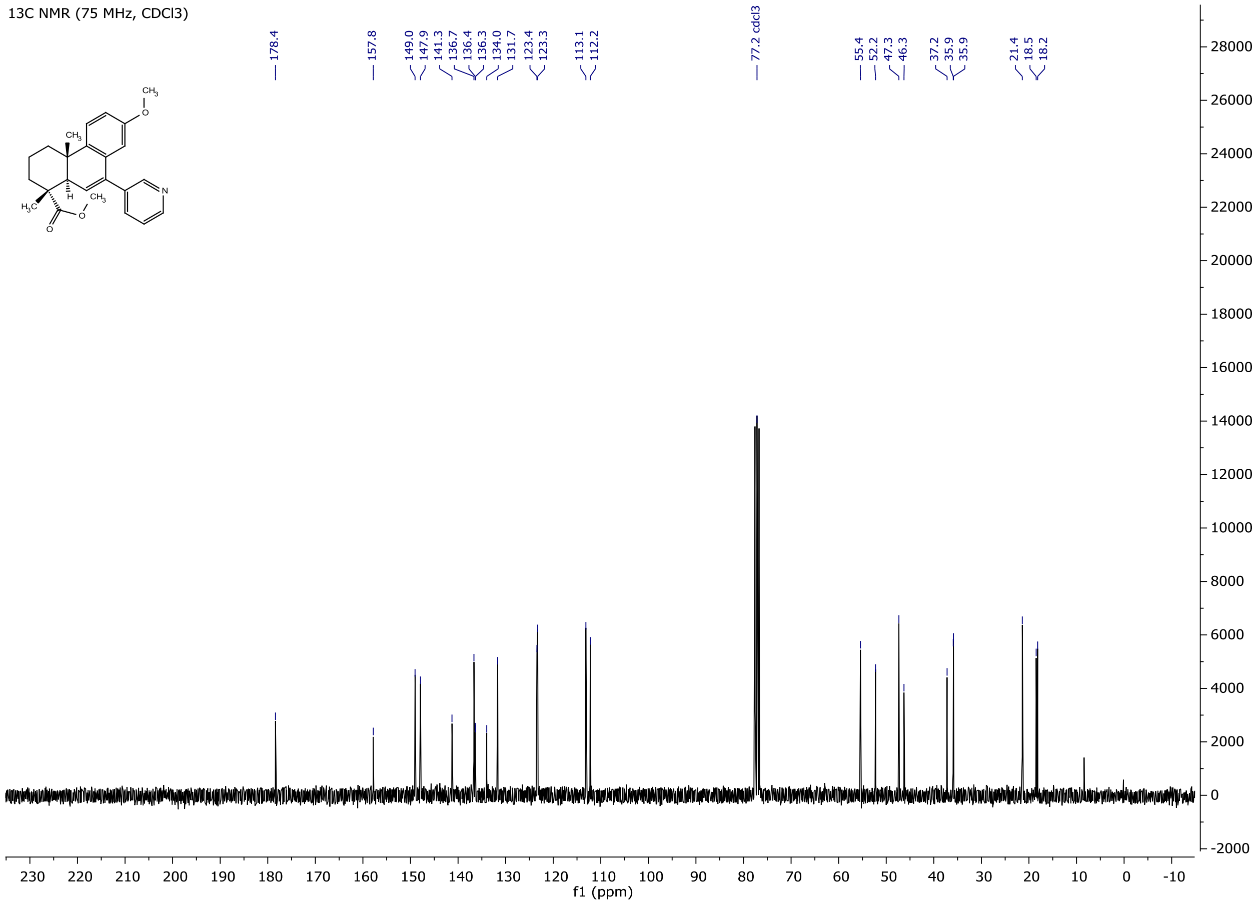

^{13}C-NMR spectrum of compound **17** recorded in $CDCl_3$

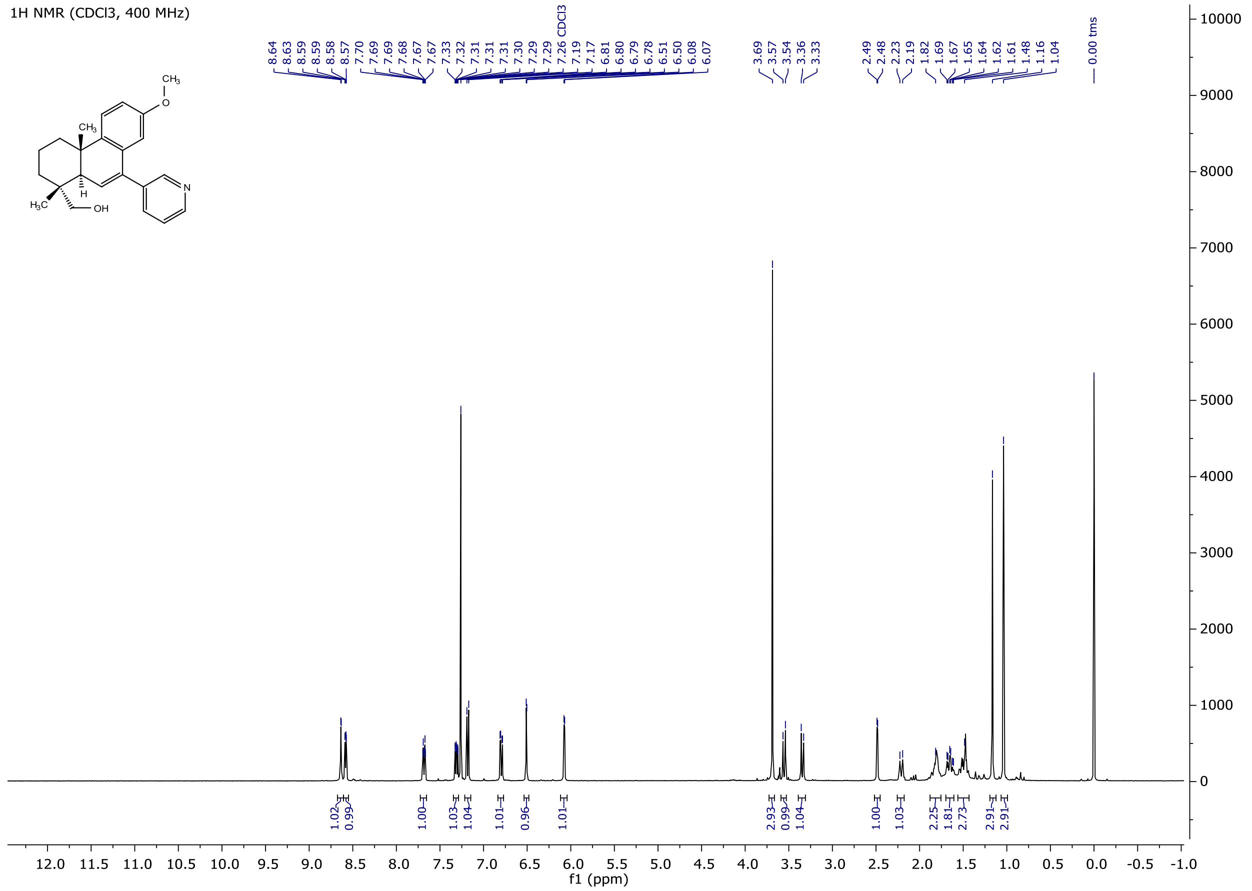

^{1}H-NMR spectrum of compound **18** recorded in $CDCl_3$

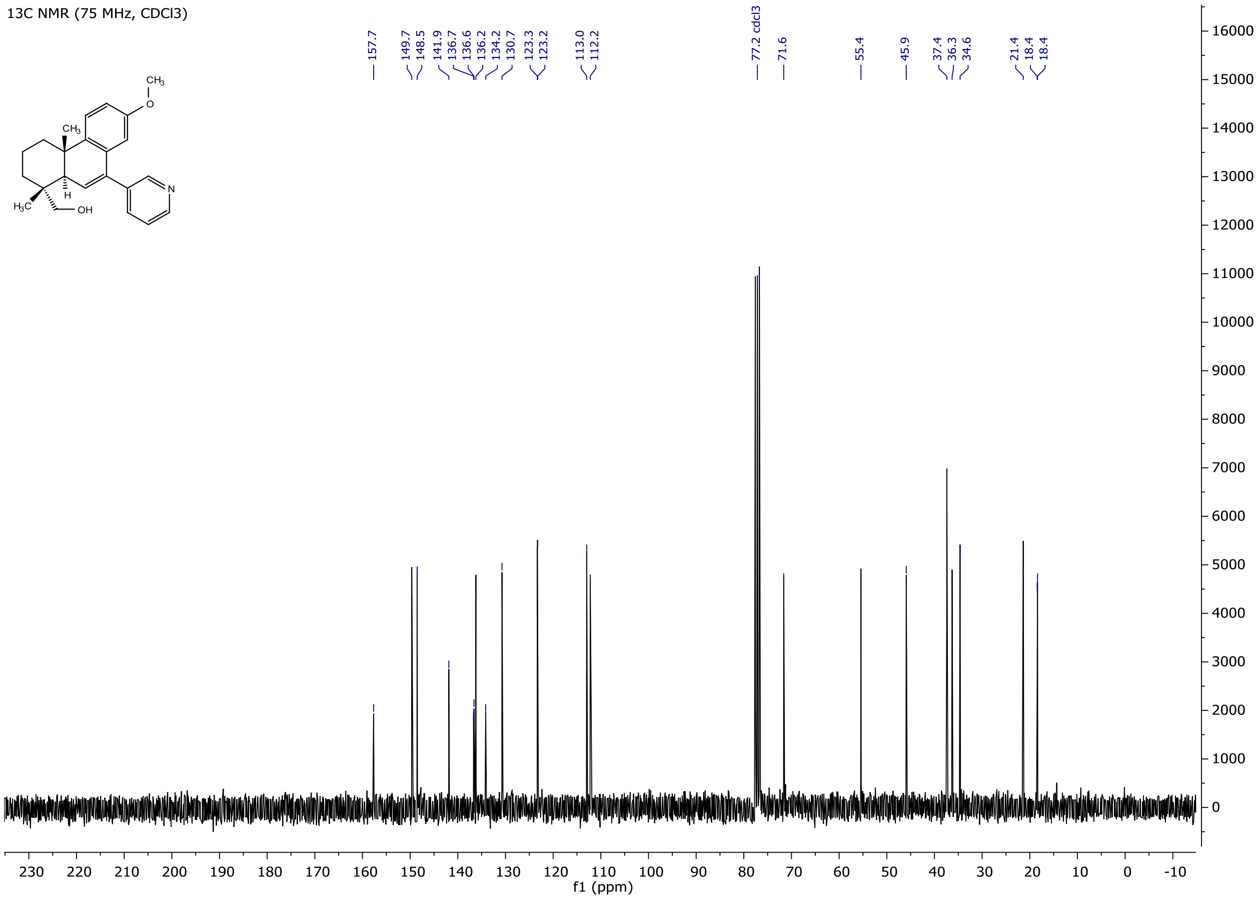

^{13}C-NMR spectrum of compound **18** recorded in $CDCl_3$

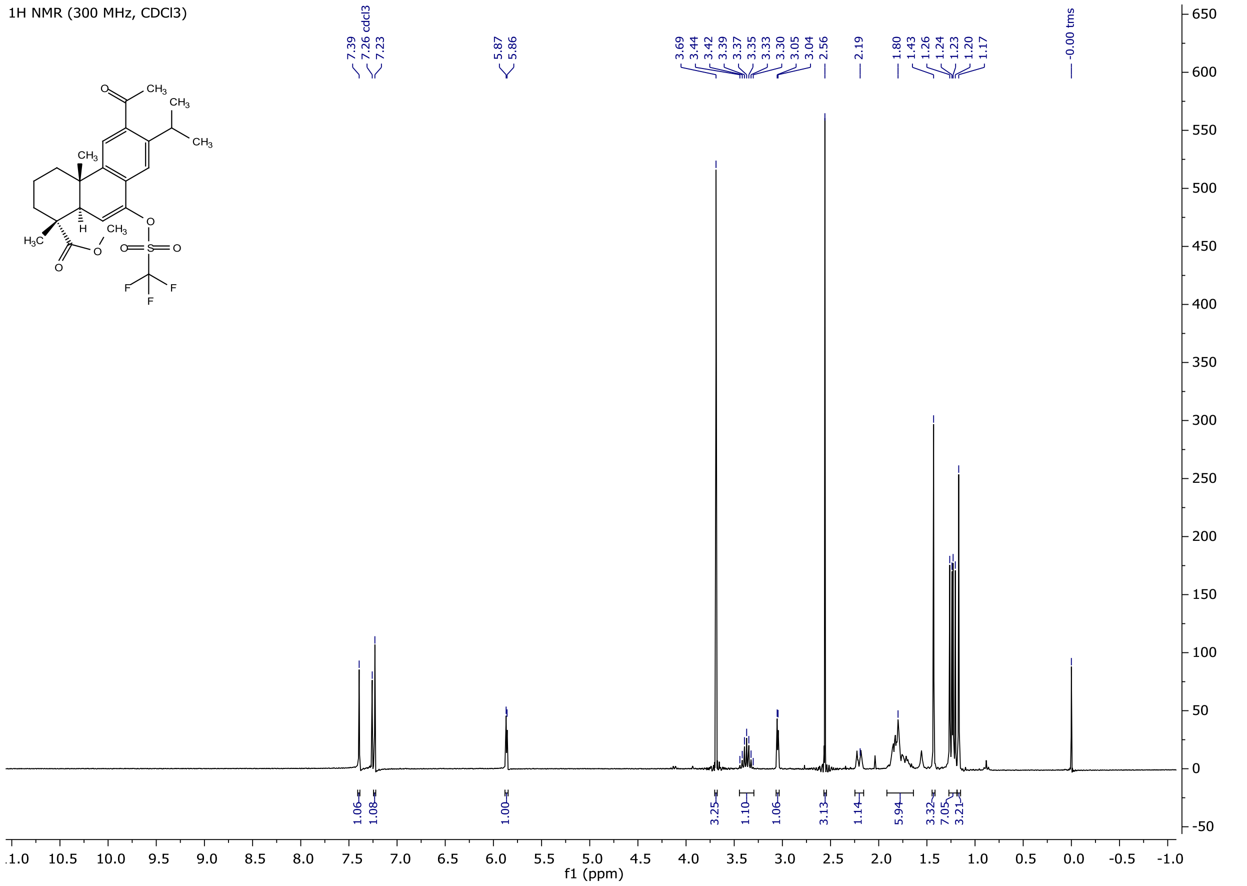

^{1}H-NMR spectrum of compound **19** recorded in $CDCl_3$

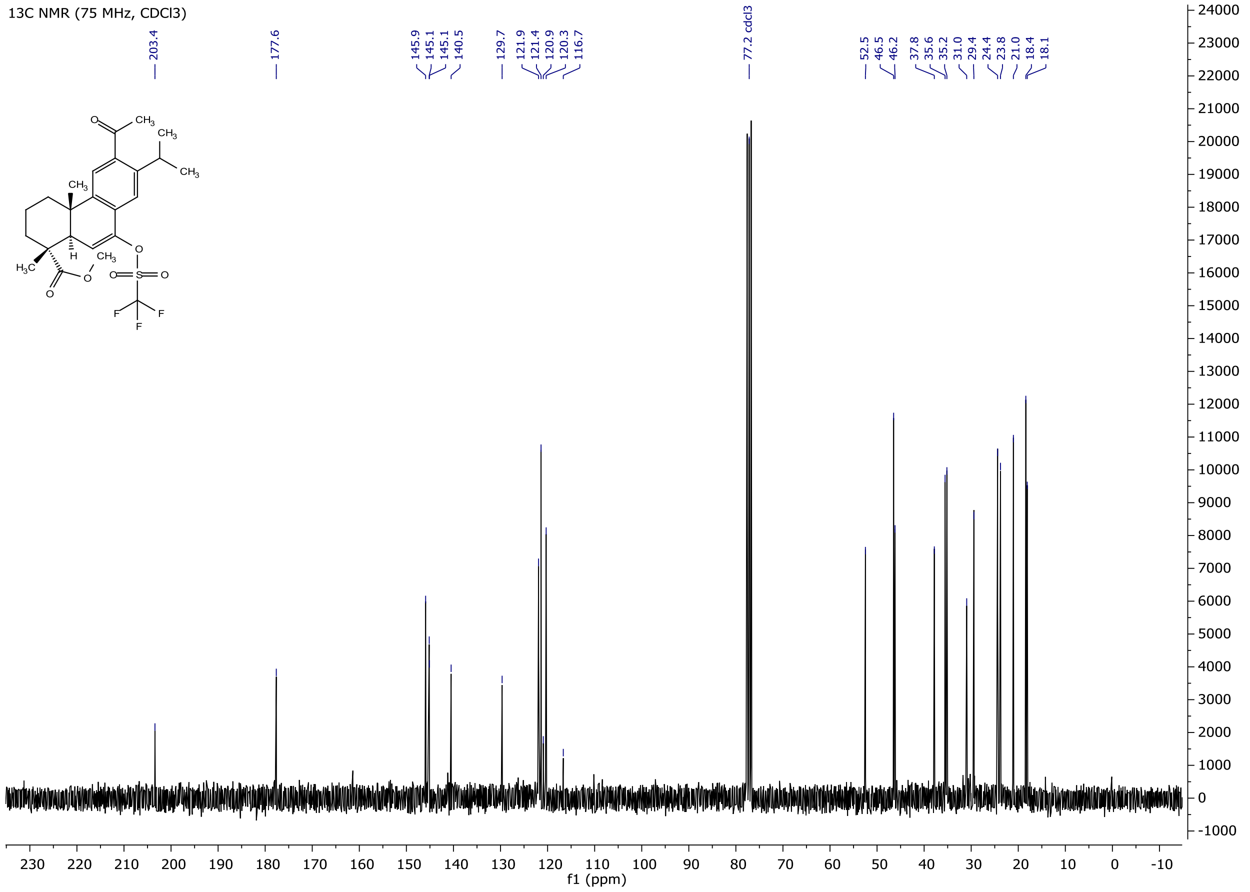

^{13}C-NMR spectrum of compound **19** recorded in $CDCl_3$

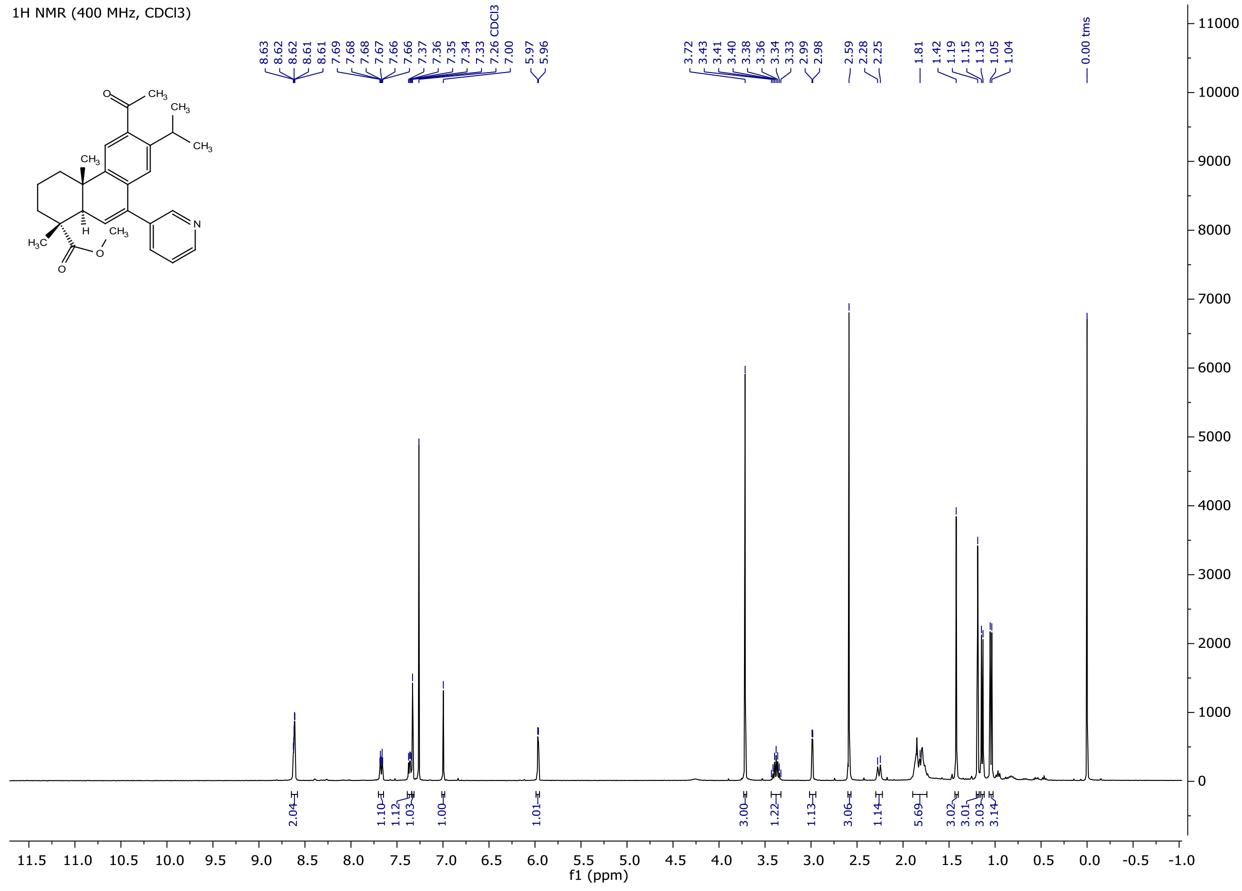

^{1}H-NMR spectrum of compound **20** recorded in $CDCl_3$

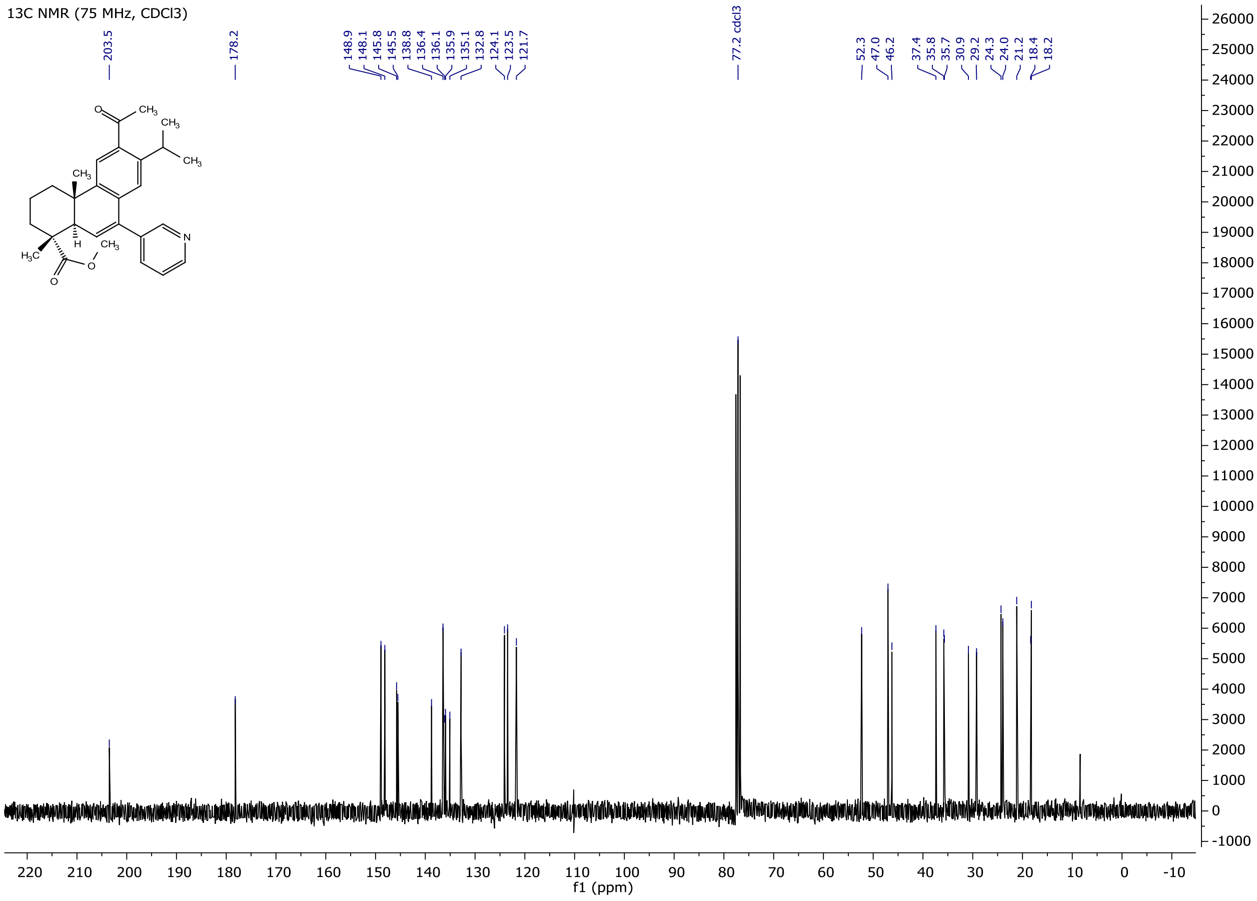

^{13}C-NMR spectrum of compound **20** recorded in $CDCl_3$

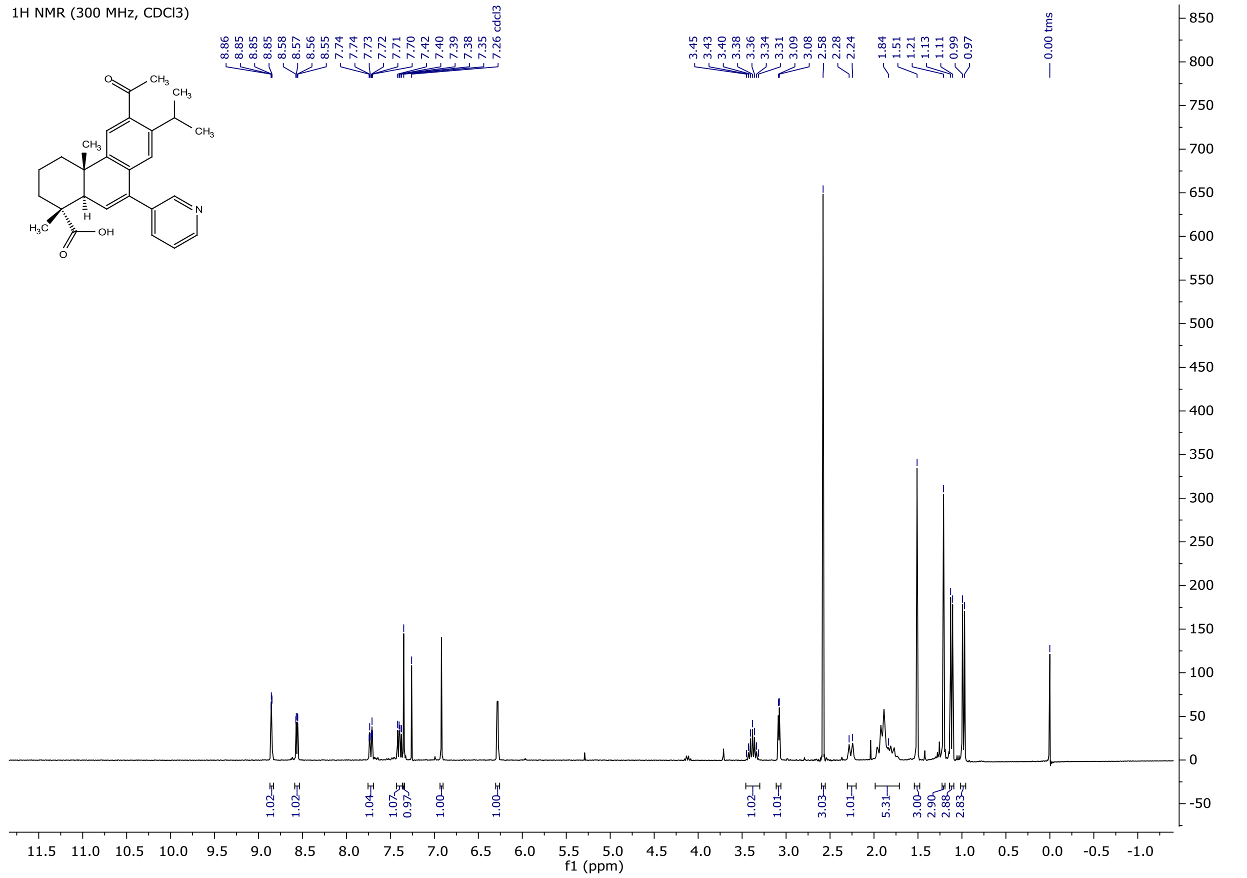

^{1}H-NMR spectrum of compound **21** recorded in $CDCl_3$

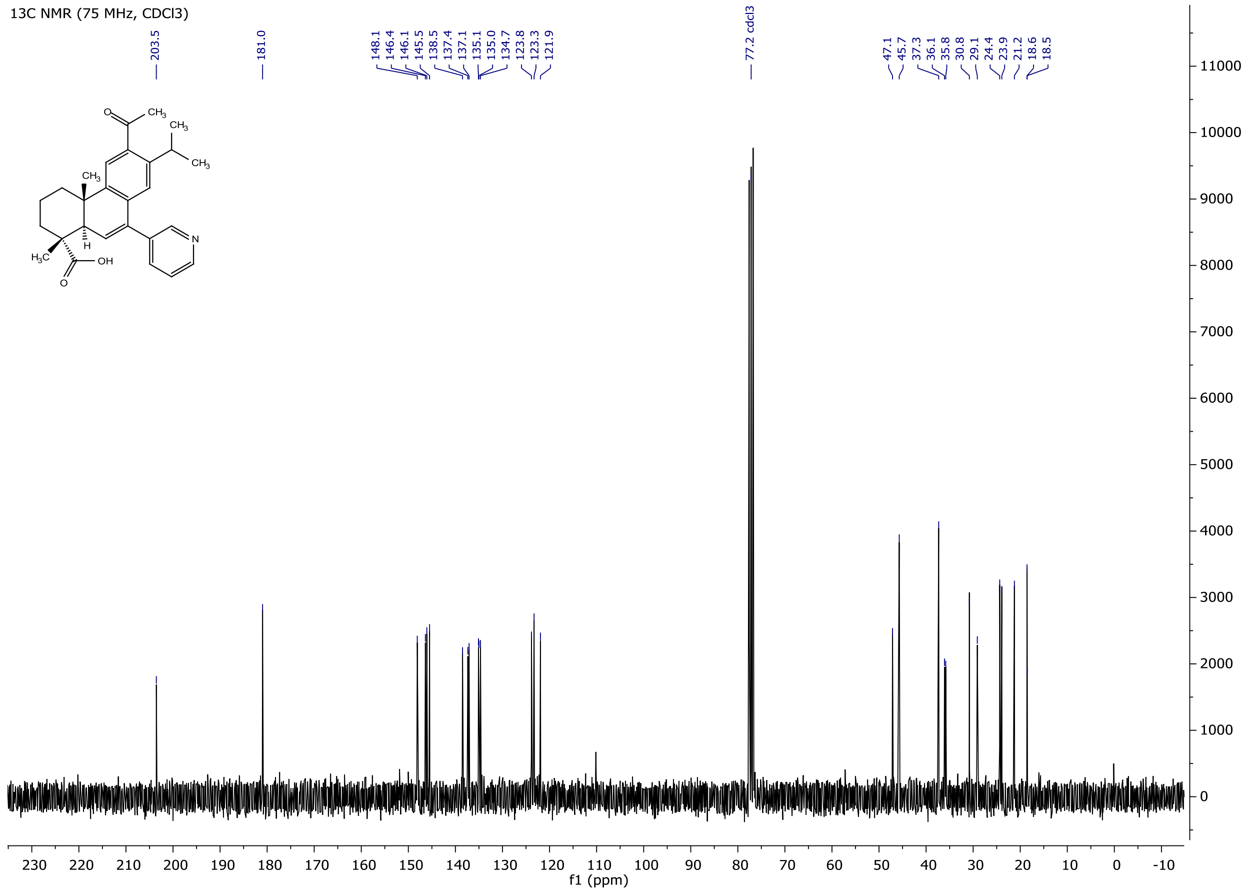

^{13}C-NMR spectrum of compound **21** recorded in $CDCl_3$

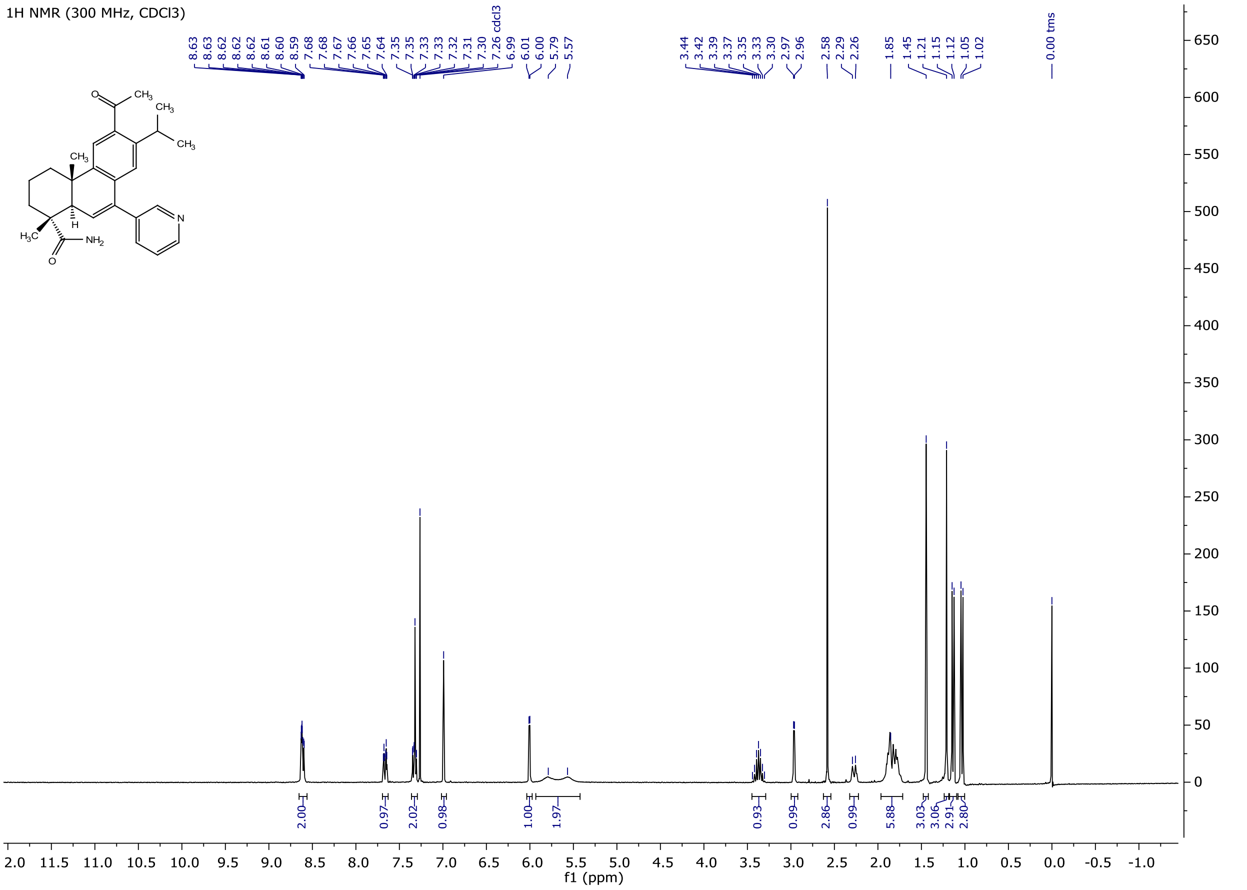

^{1}H-NMR spectrum of compound **22** recorded in $CDCl_3$

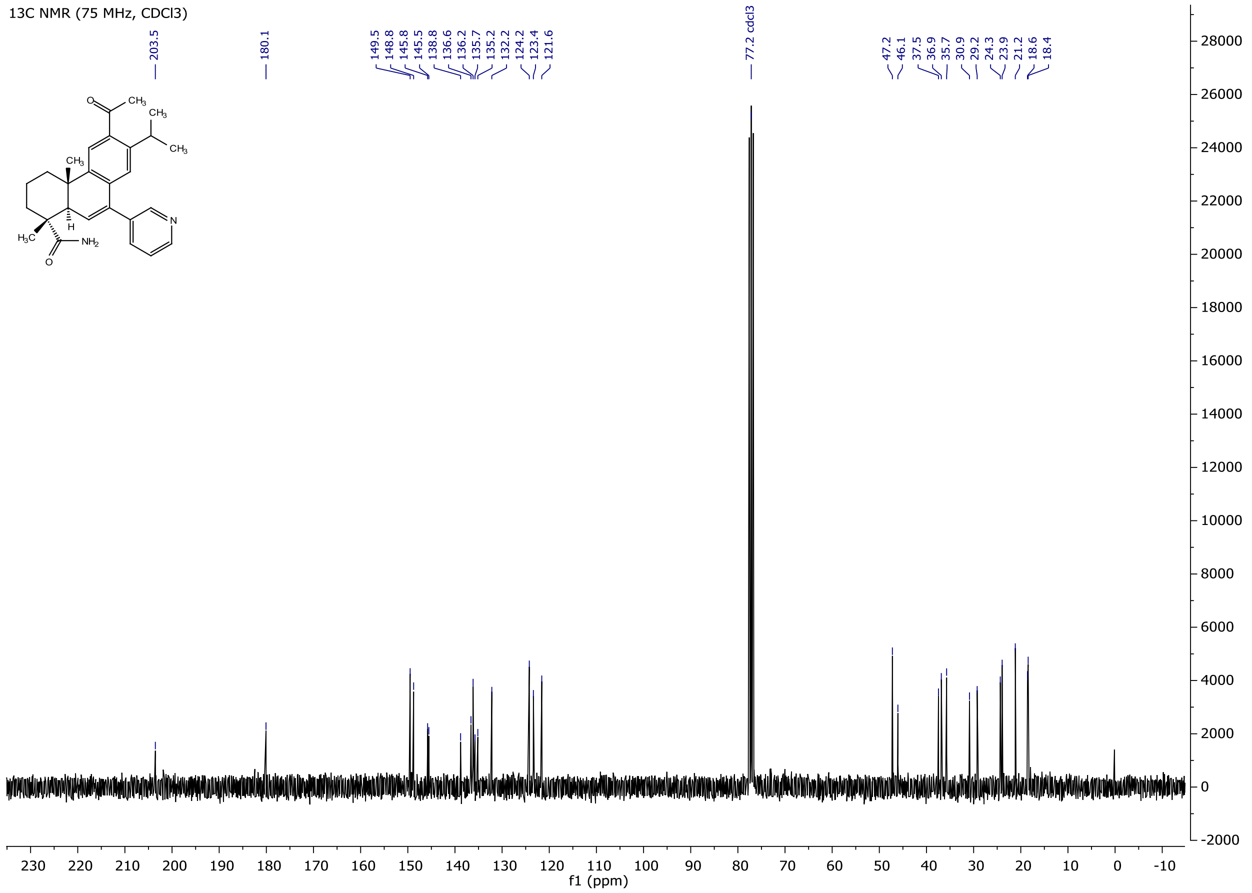

^{13}C-NMR spectrum of compound **22** recorded in $CDCl_3$

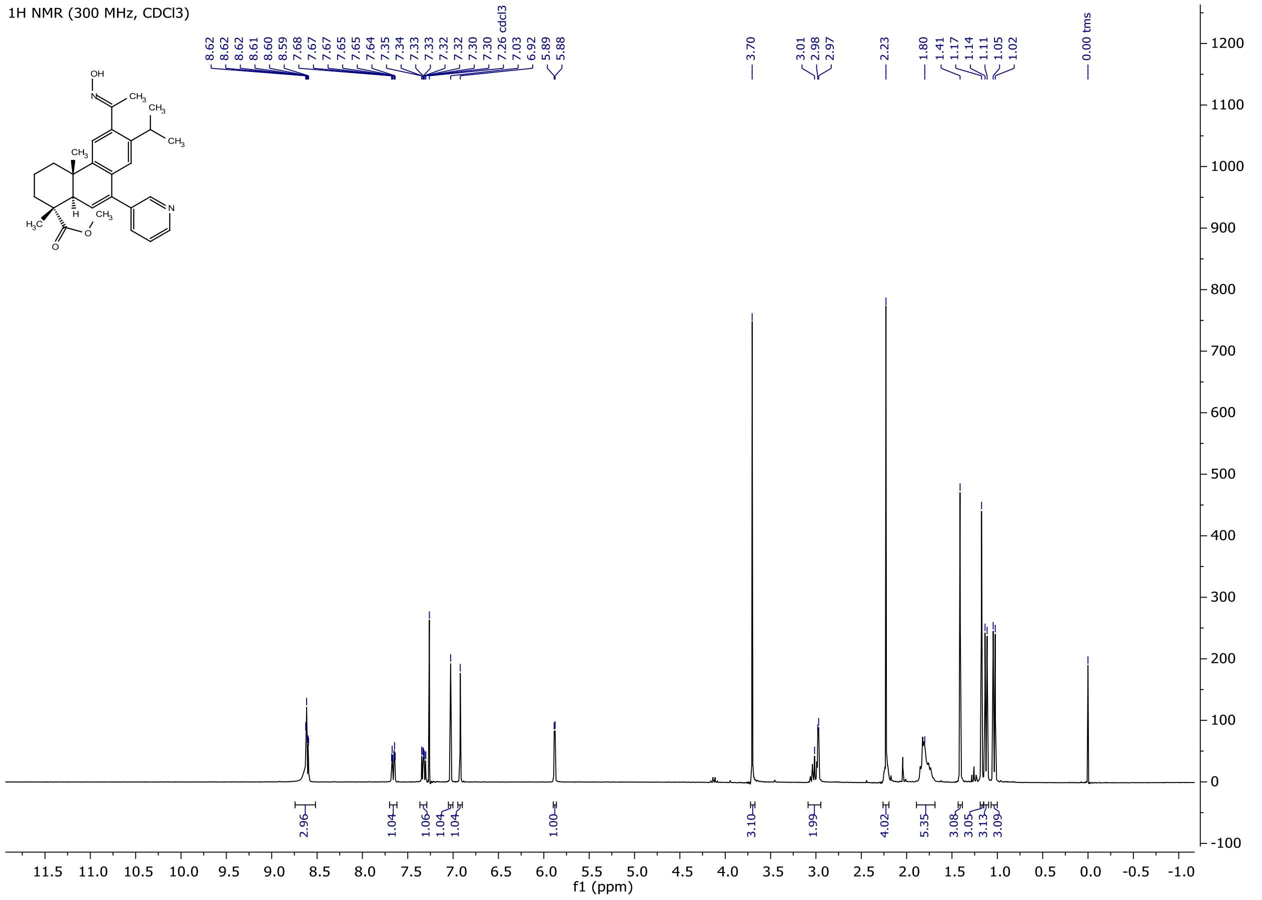

^{1}H-NMR spectrum of compound **23** recorded in $CDCl_3$

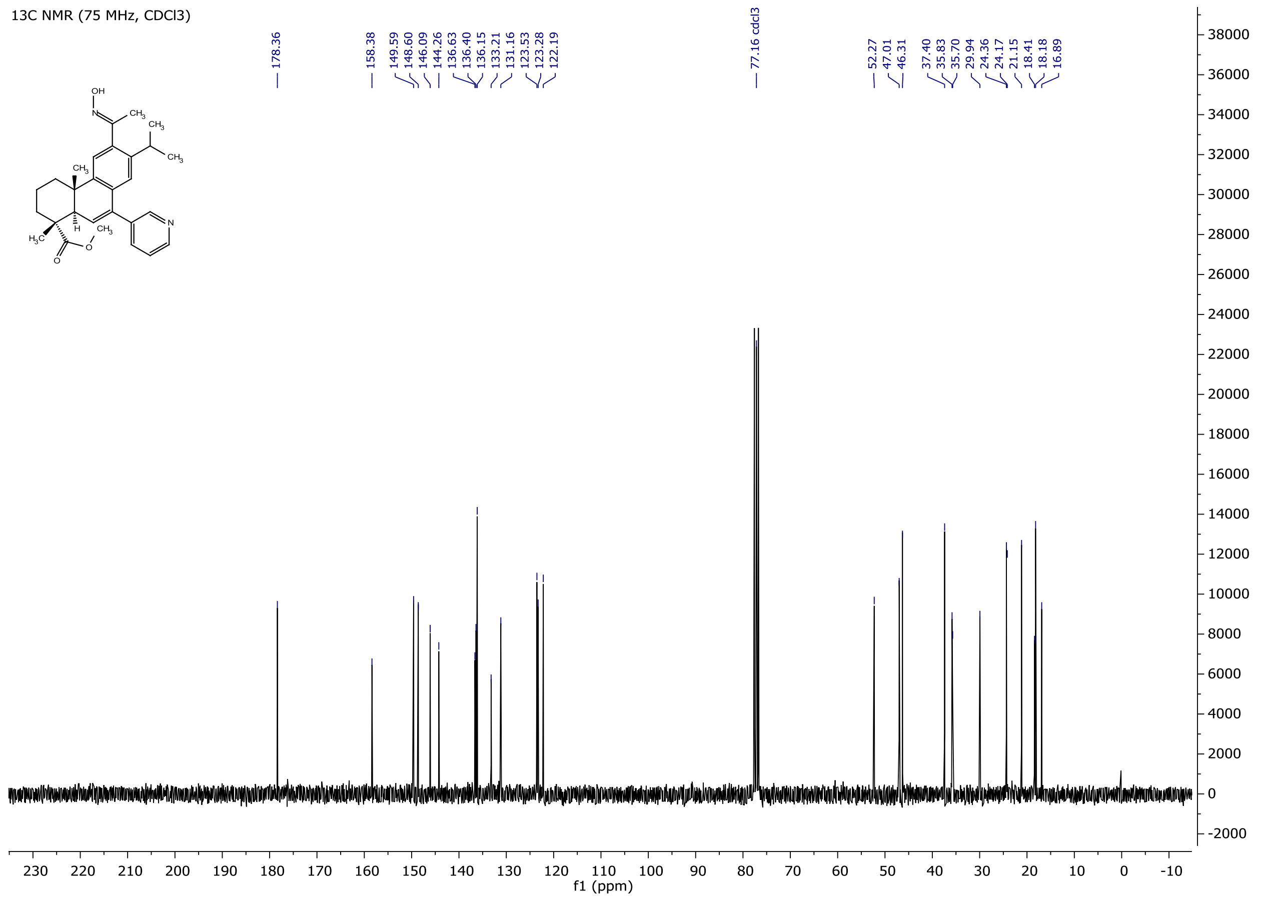

^{13}C-NMR spectrum of compound **23** recorded in $CDCl_3$

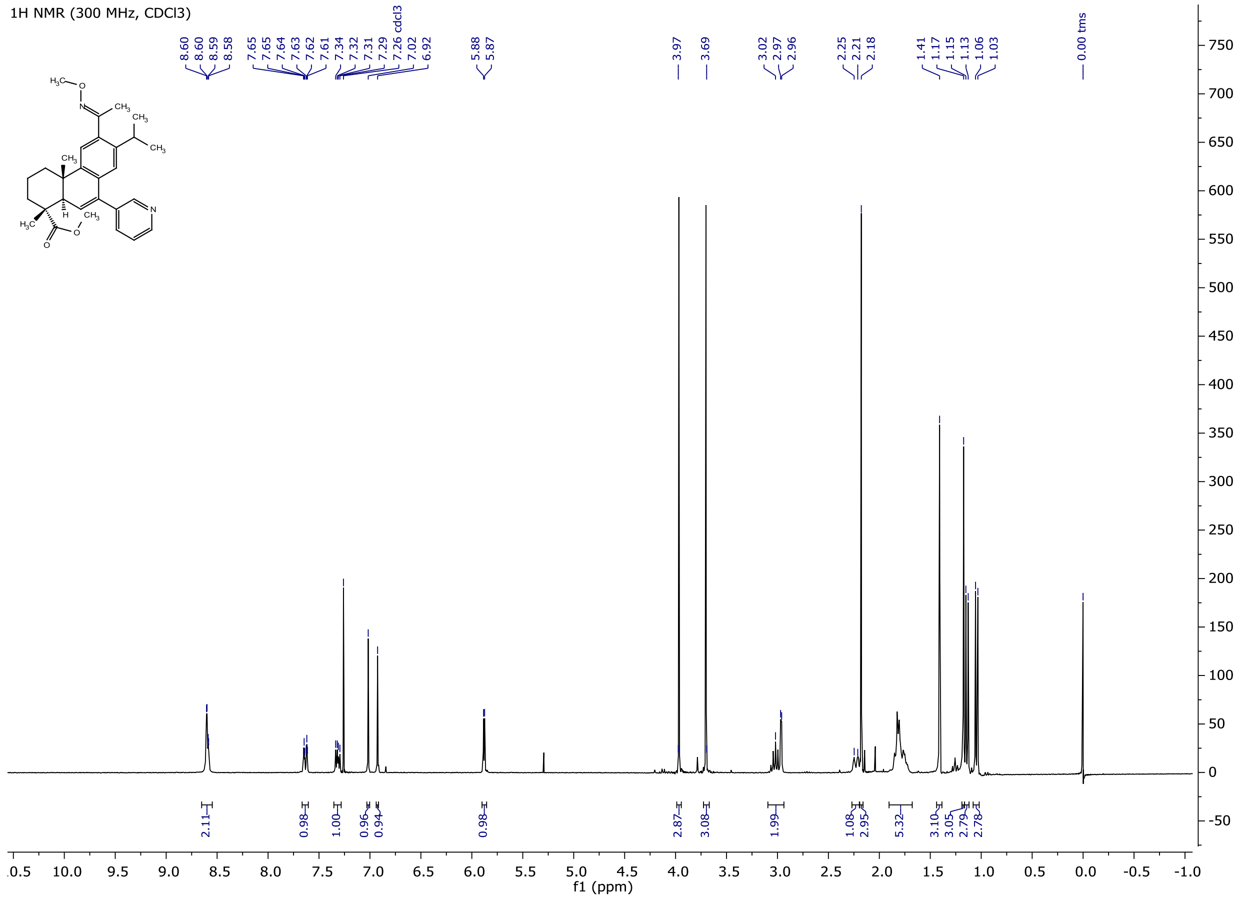

^{1}H-NMR spectrum of compound **24** recorded in $CDCl_3$

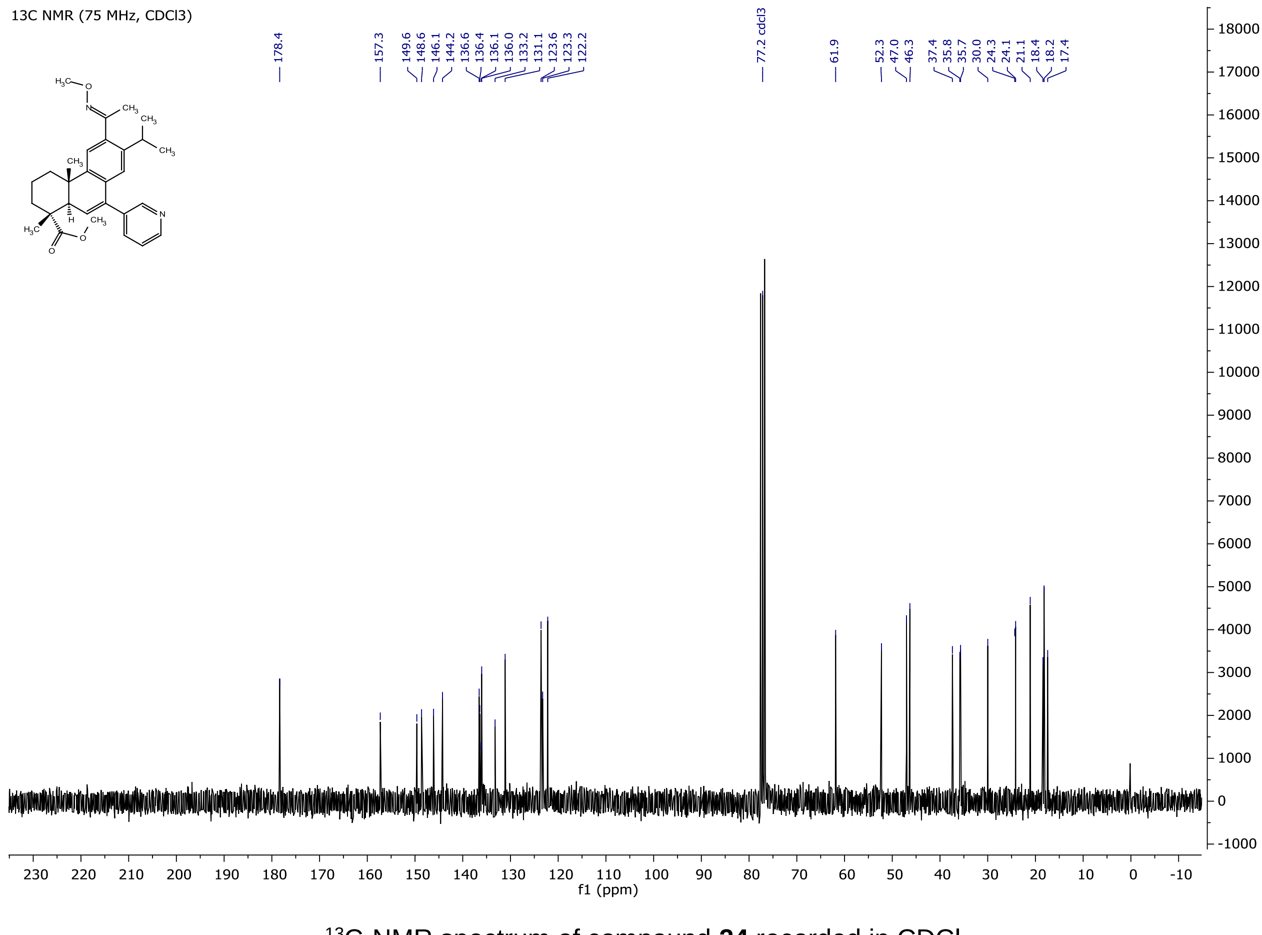

^{13}C-NMR spectrum of compound **24** recorded in $CDCl_3$

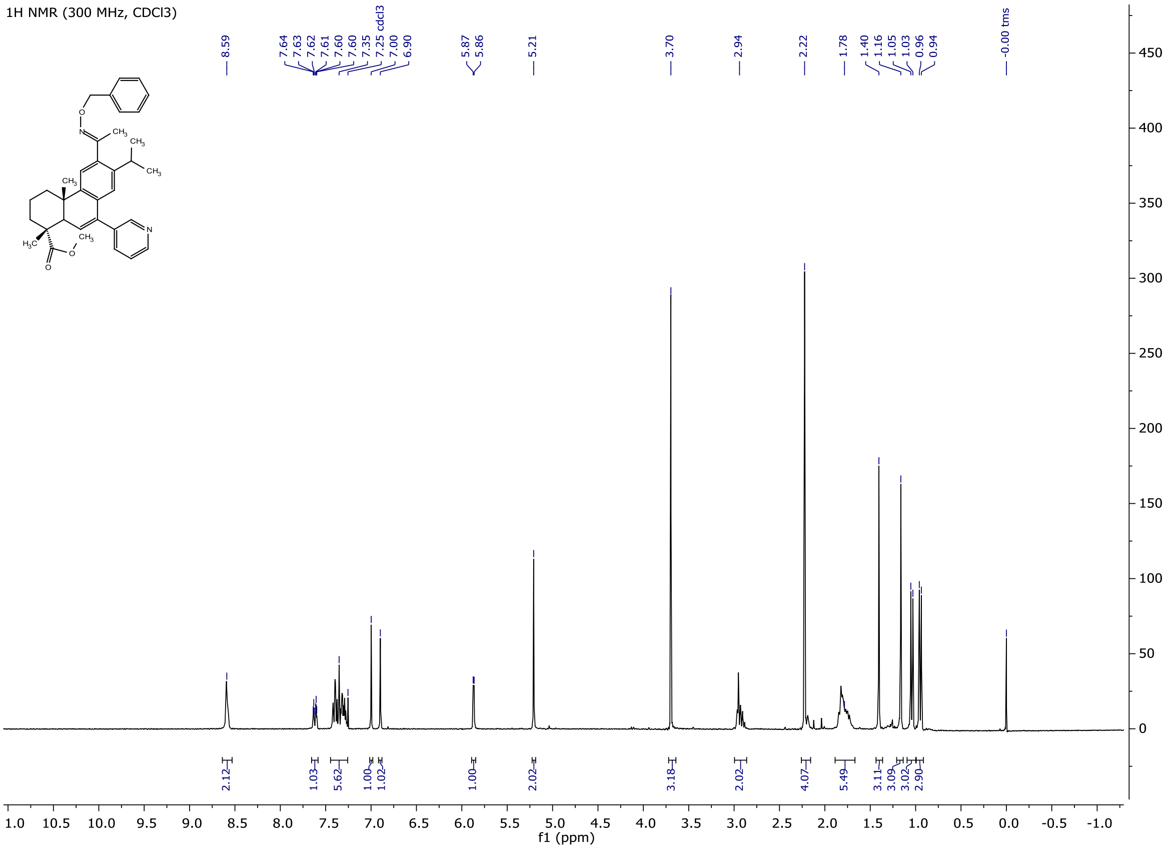

^{1}H-NMR spectrum of compound **25** recorded in $CDCl_3$

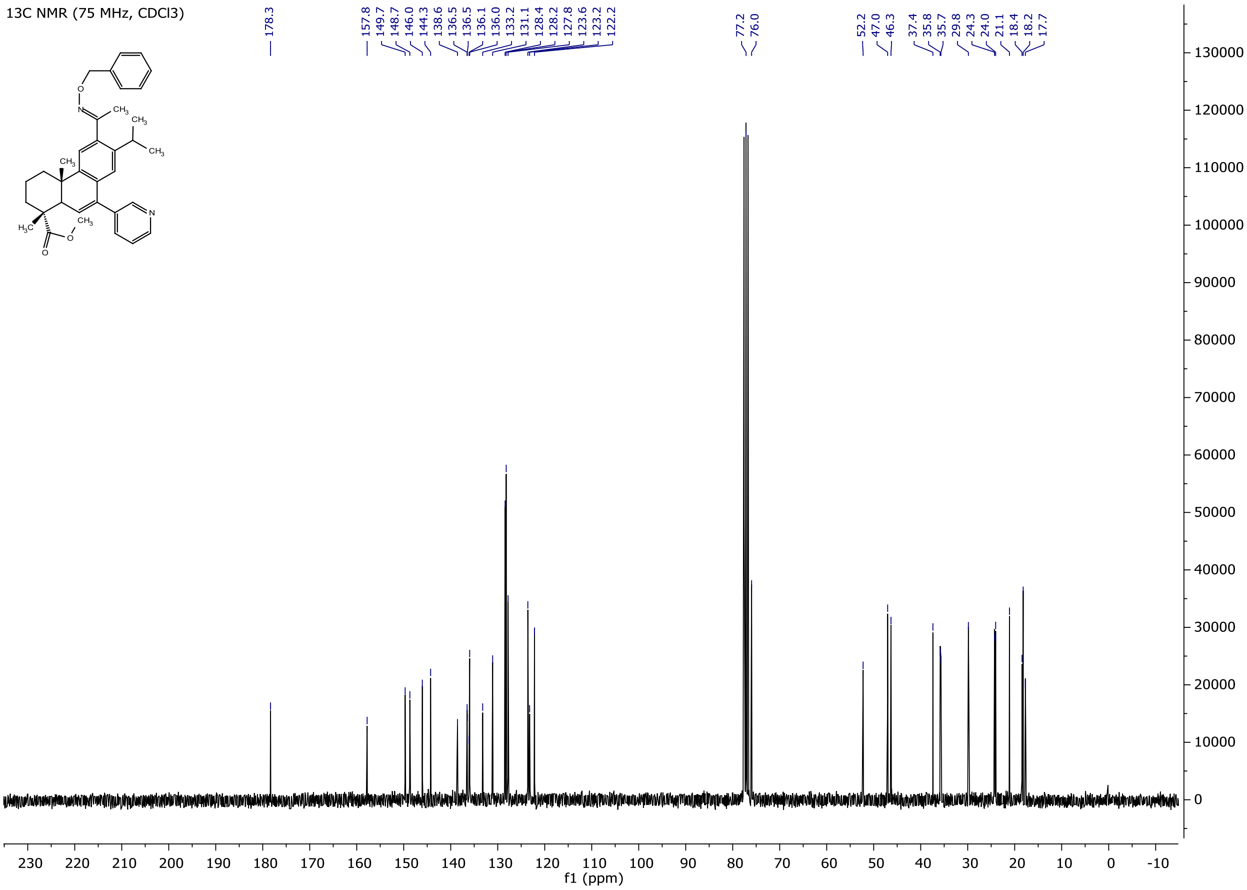

^{13}C-NMR spectrum of compound **25** recorded in $CDCl_3$

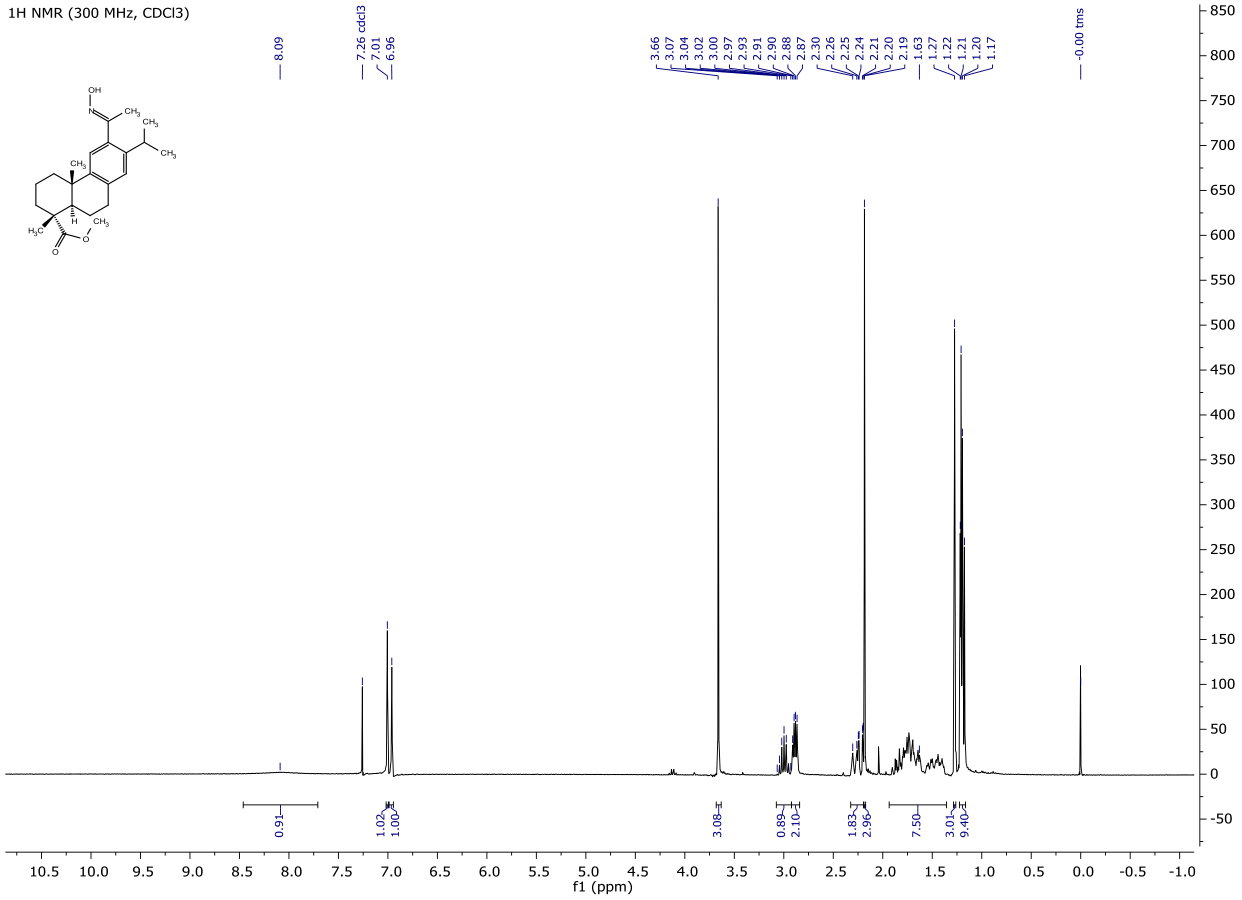

^{1}H-NMR spectrum of compound **26** recorded in $CDCl_3$

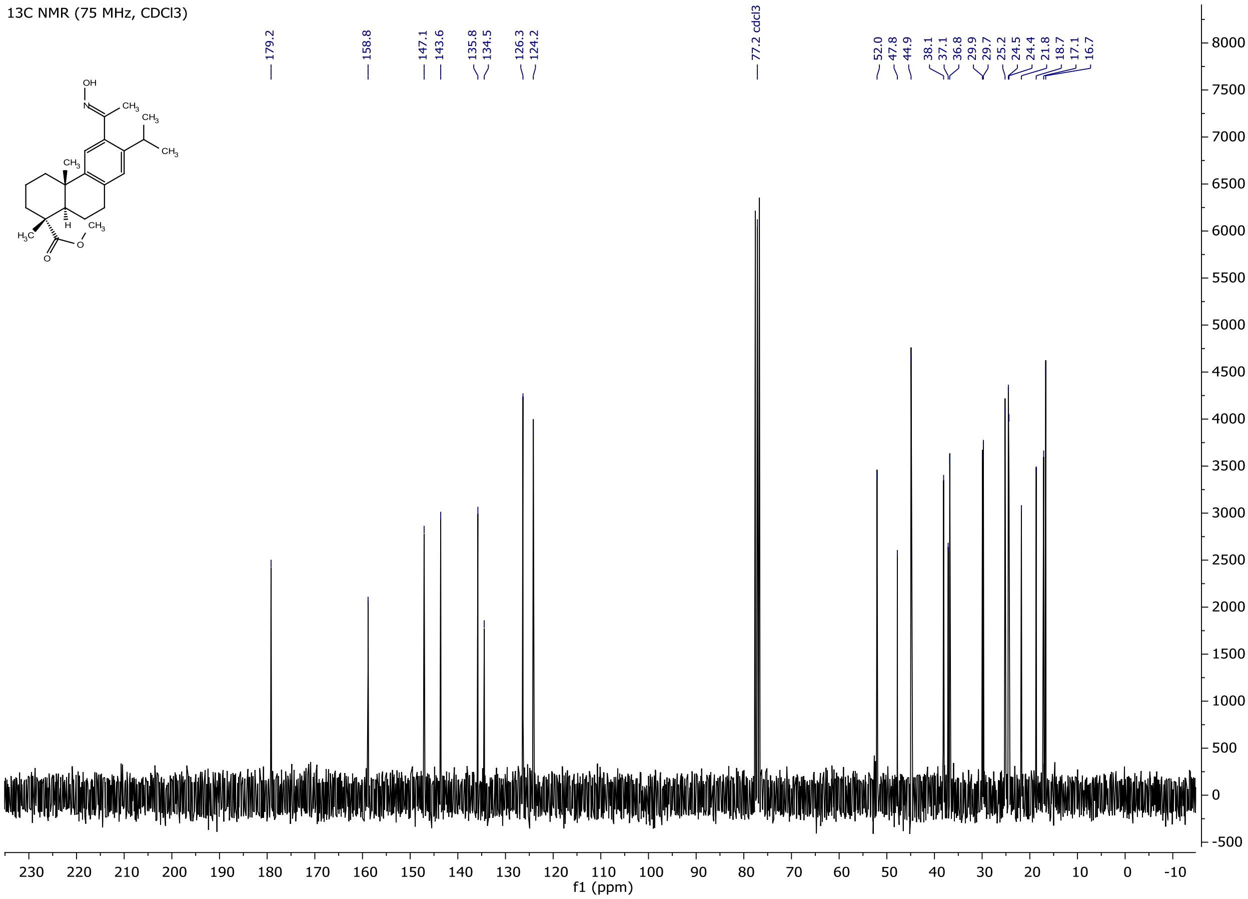

^{13}C-NMR spectrum of compound **26** recorded in $CDCl_3$

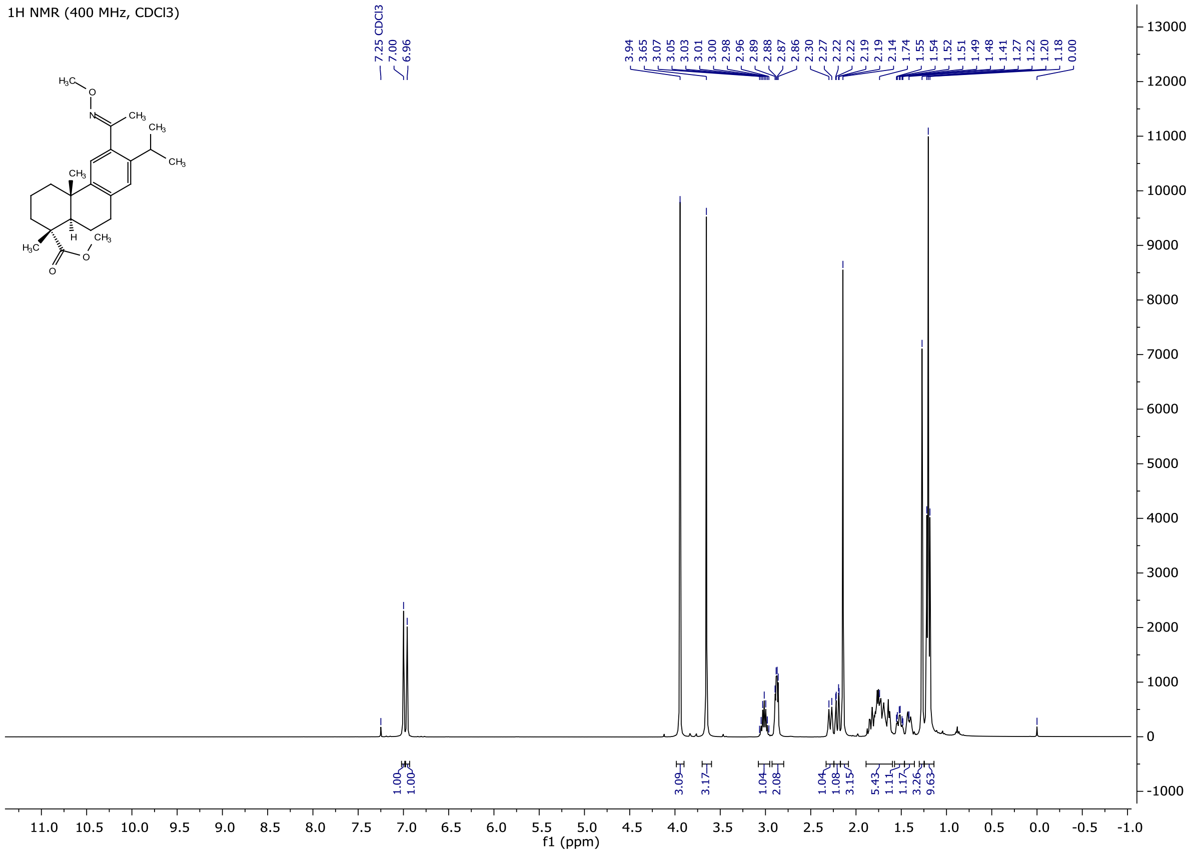

^{1}H-NMR spectrum of compound **27** recorded in $CDCl_3$

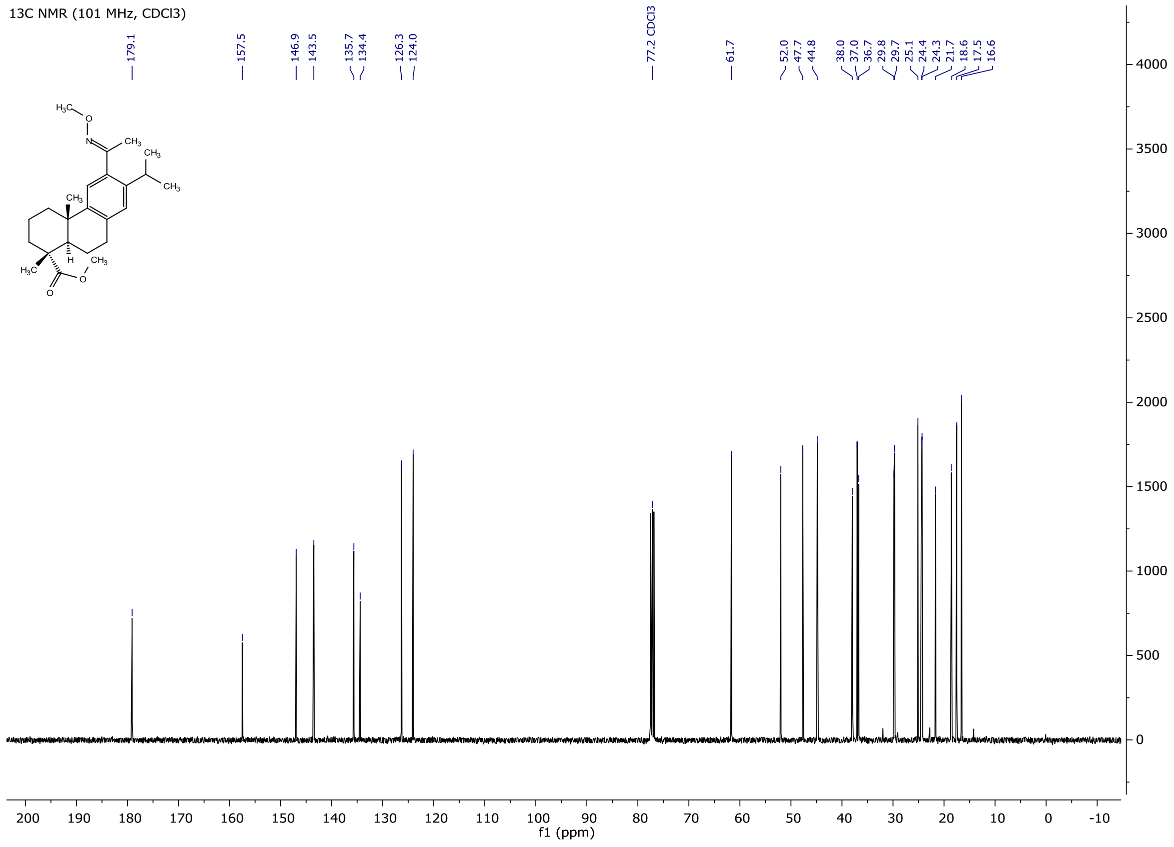

^{13}C-NMR spectrum of compound **27** recorded in $CDCl_3$

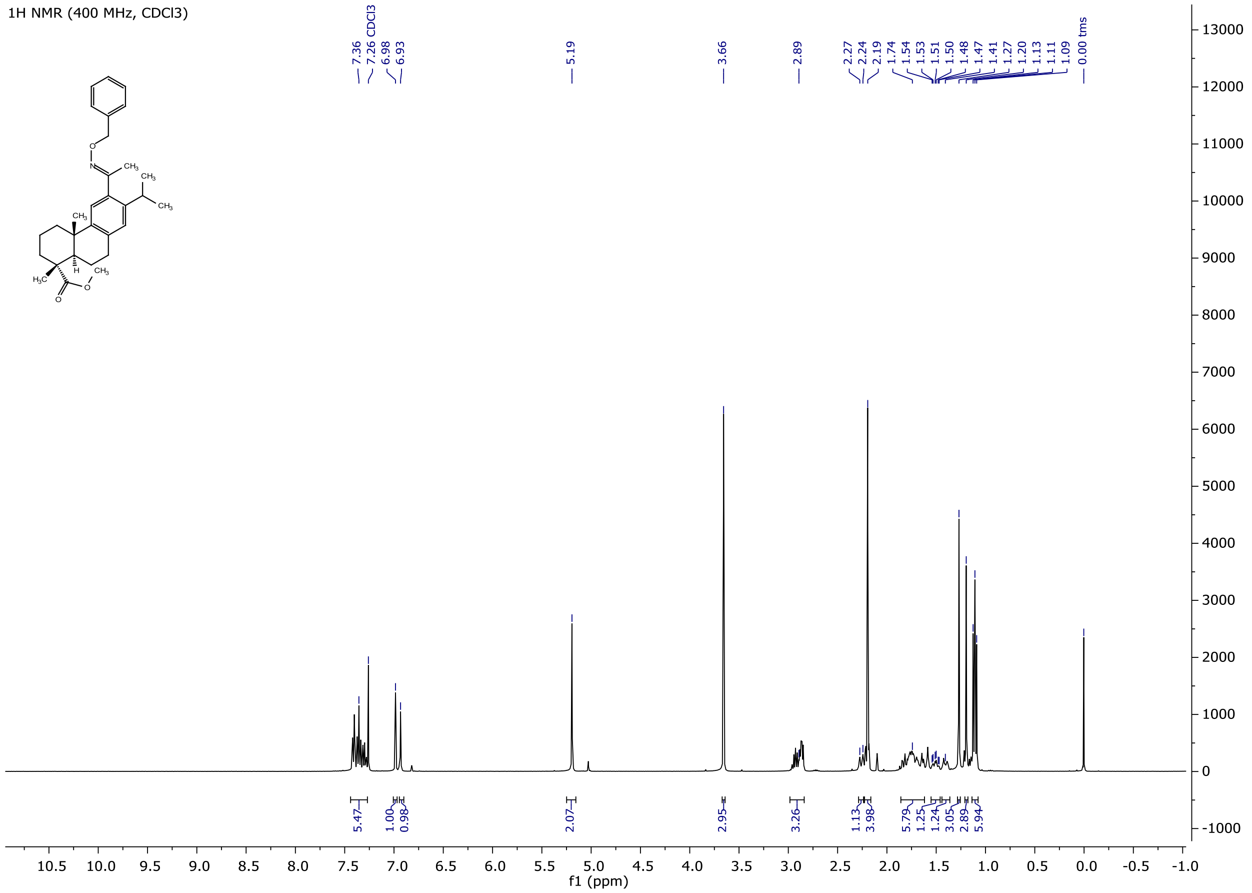

^{1}H-NMR spectrum of compound **28** recorded in $CDCl_3$

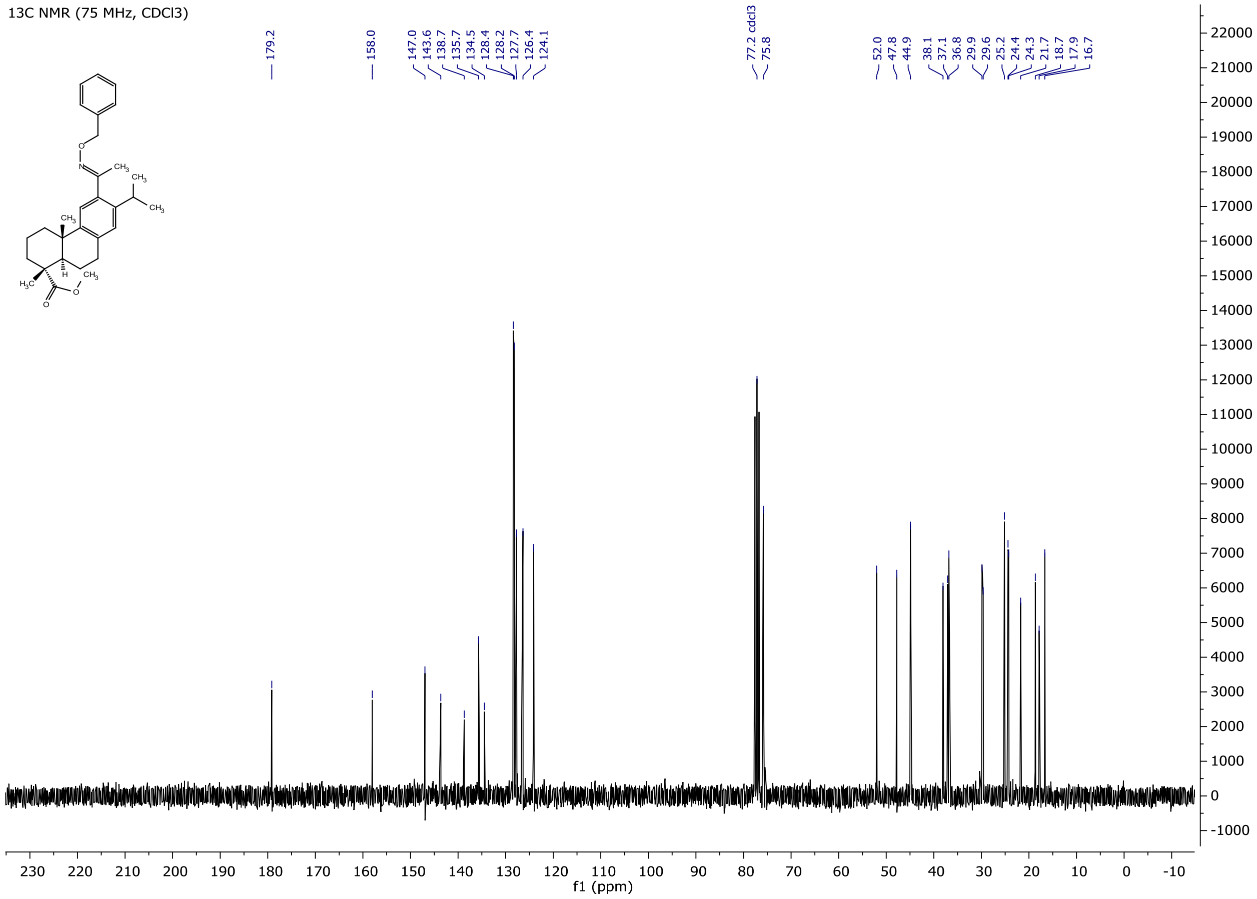

^{13}C-NMR spectrum of compound **28** recorded in $CDCl_3$

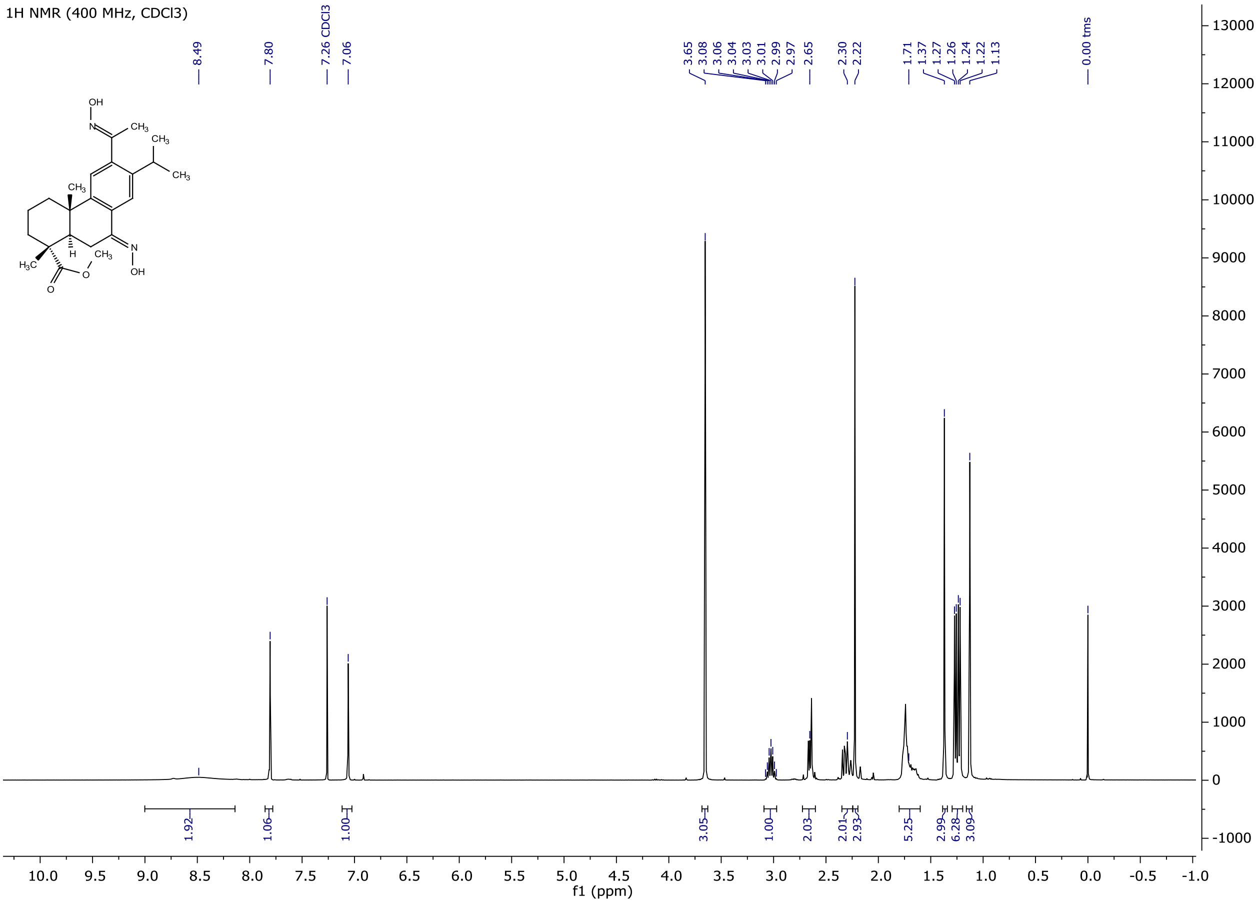

^{1}H-NMR spectrum of compound **29** recorded in $CDCl_3$

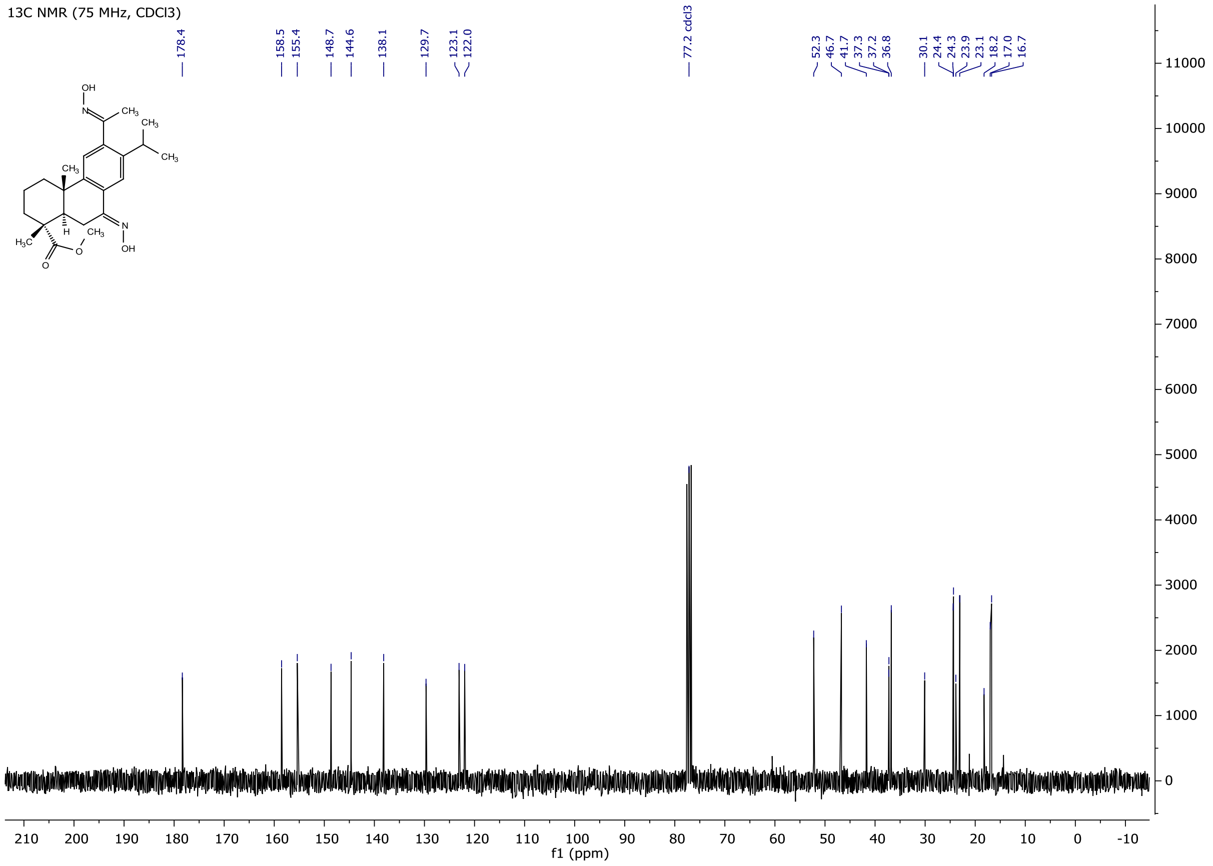

^{13}C-NMR spectrum of compound **29** recorded in $CDCl_3$

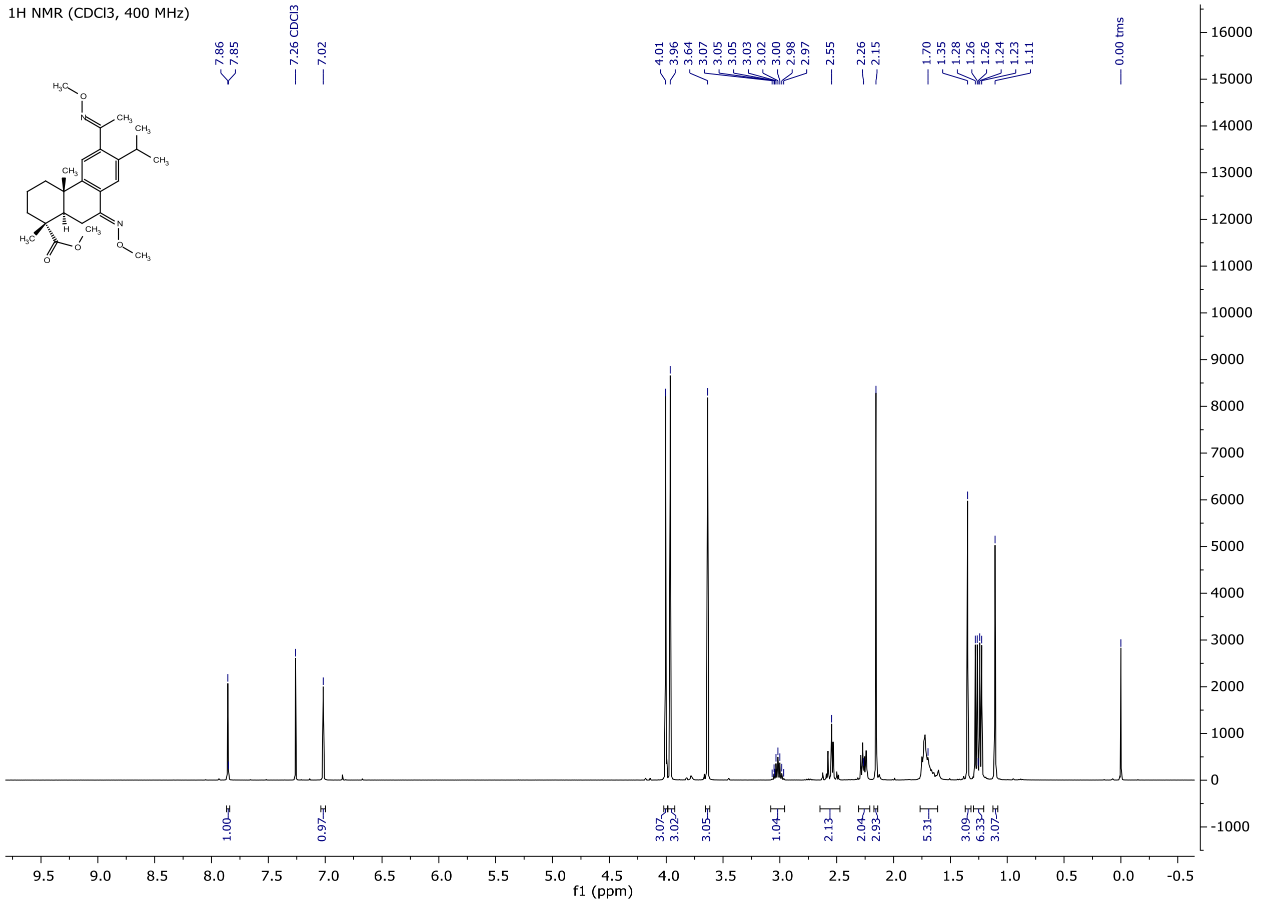

^{1}H-NMR spectrum of compound **30** recorded in $CDCl_3$

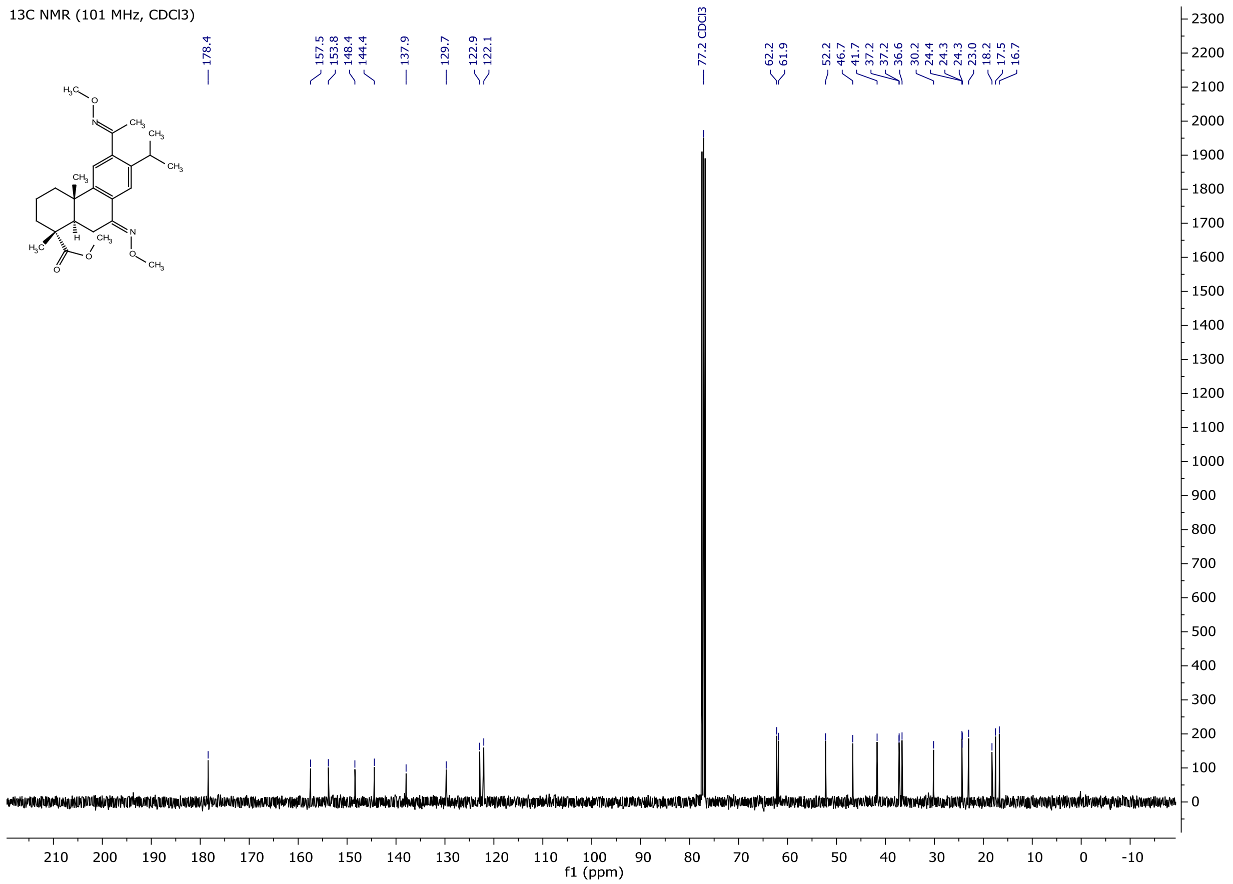

^{13}C-NMR spectrum of compound **30** recorded in $CDCl_3$

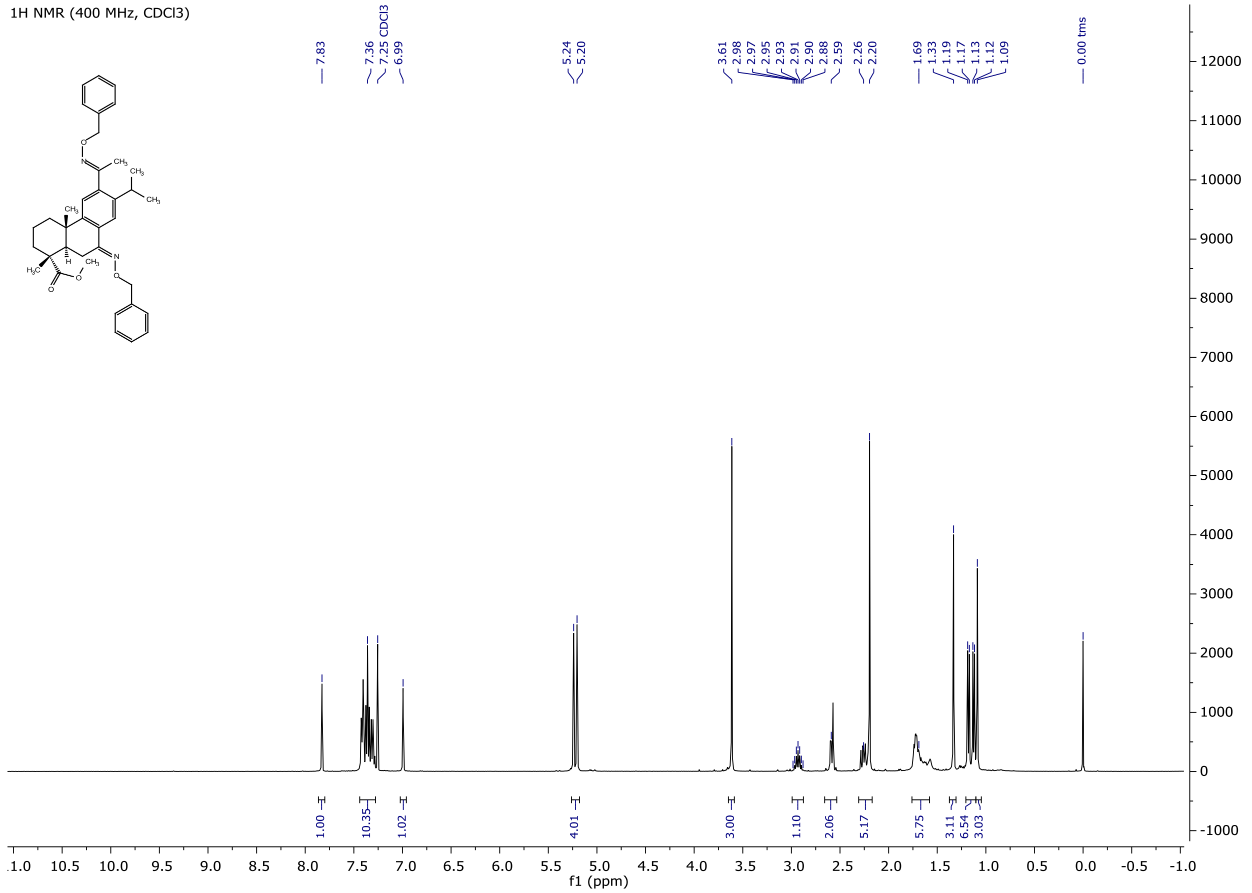

^{1}H-NMR spectrum of compound **31** recorded in $CDCl_3$

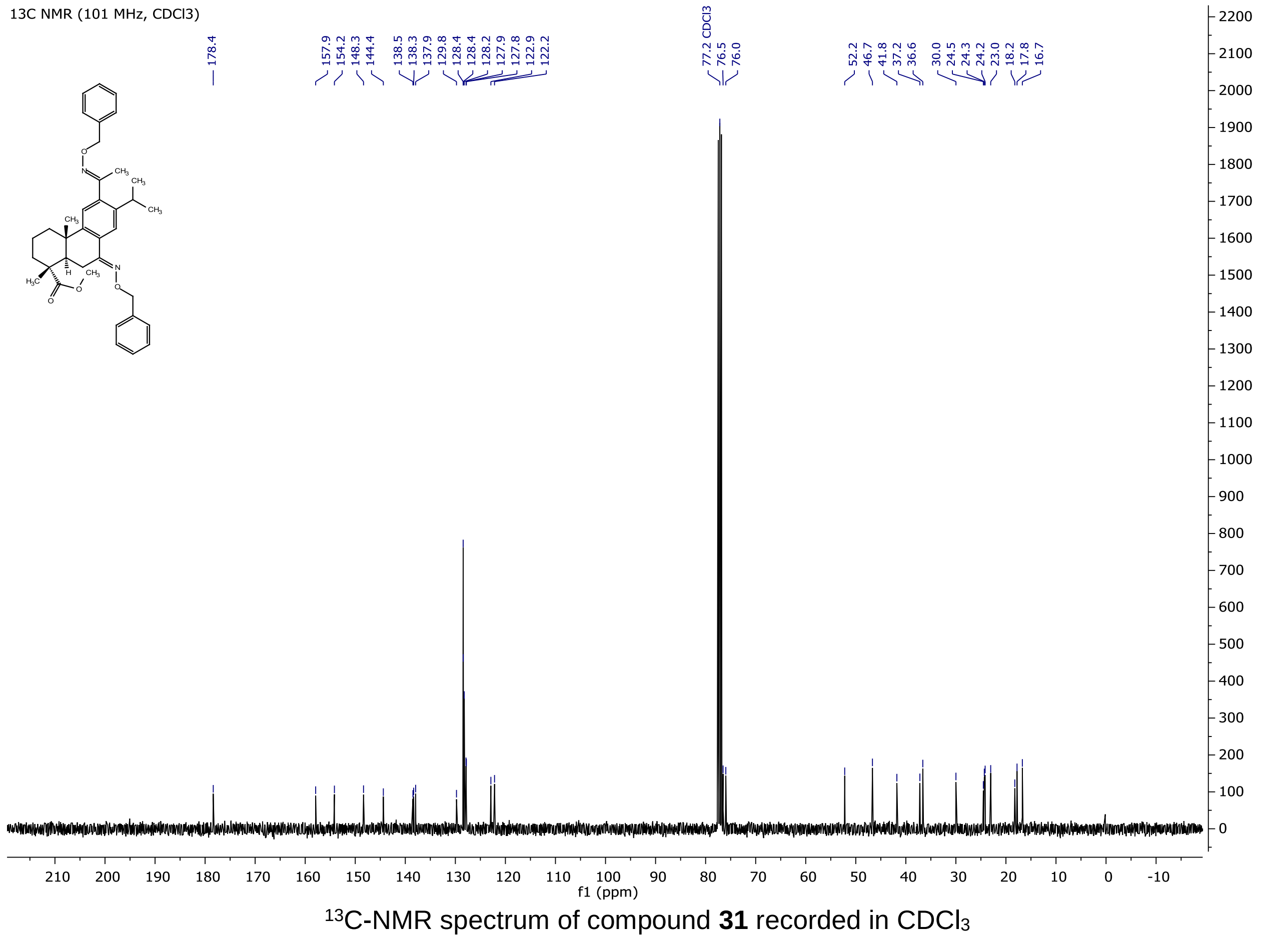

^{13}C-NMR spectrum of compound **31** recorded in $CDCl_3$

Original bands for Western blot studies

Bands selected for Figure 4 are marked with a rectangle.

Aspc-1 cells treated 48 hours

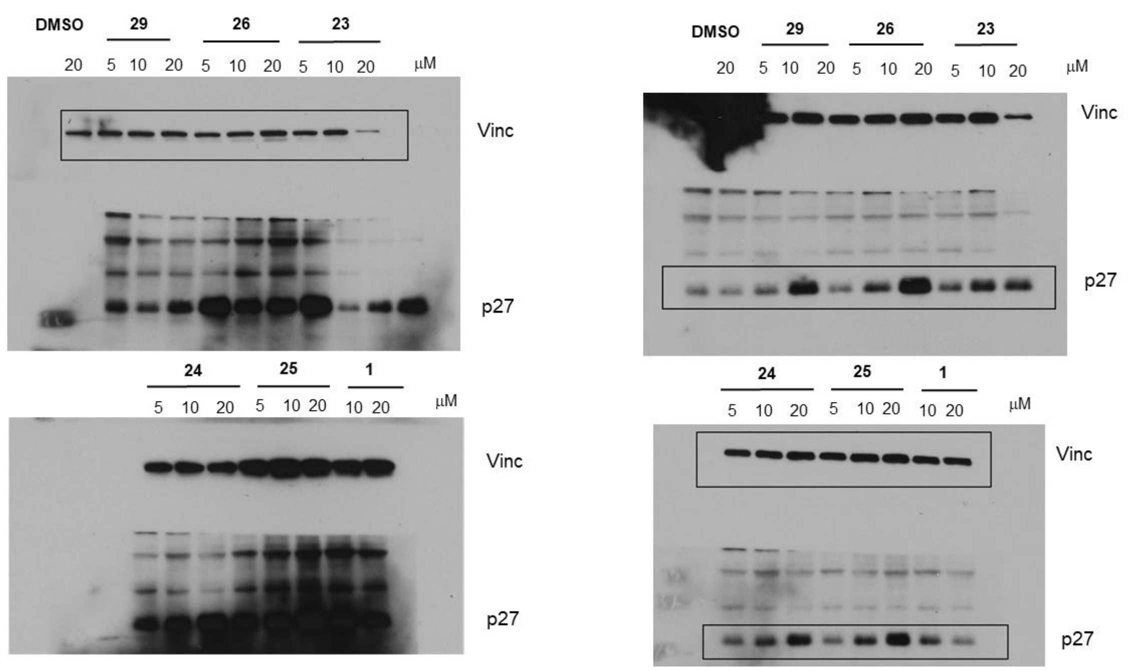

PanAsc 2159 cells treated 48 hours

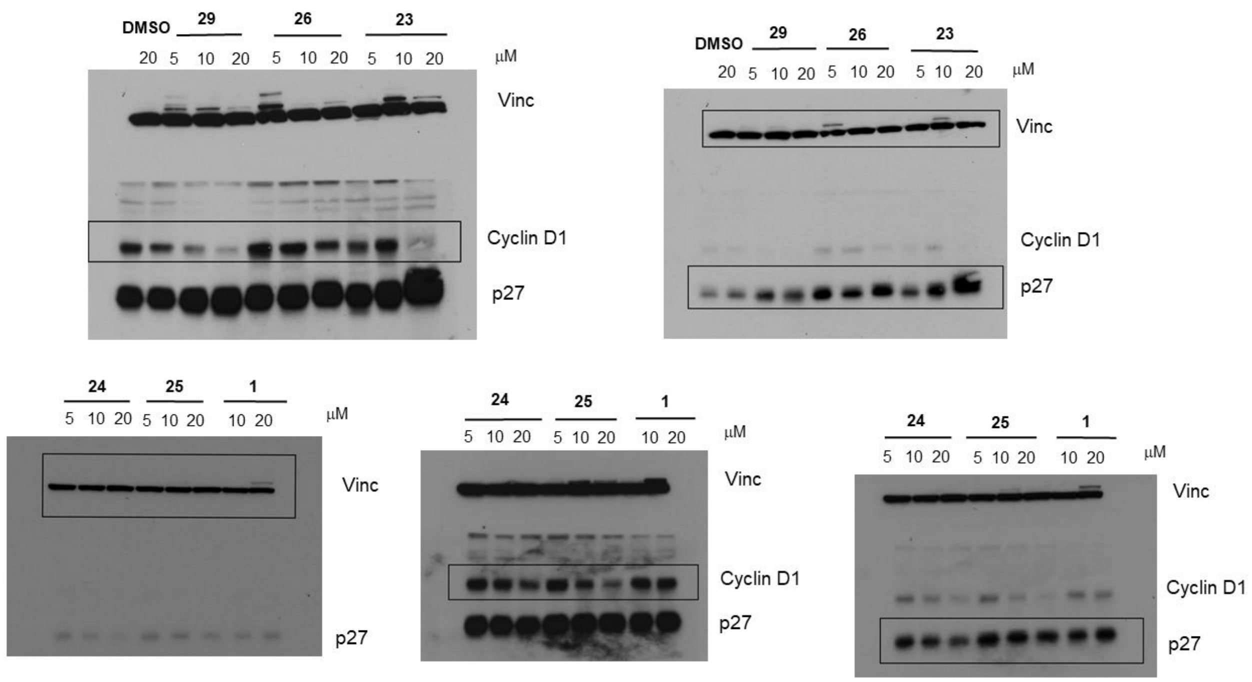

Cell cycle analysis data

A

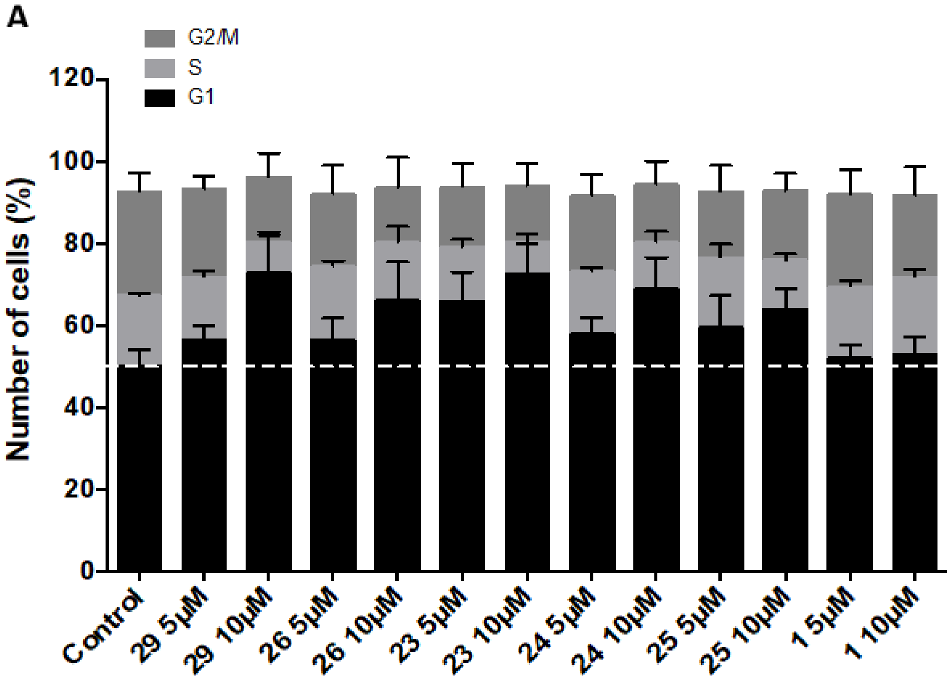

B

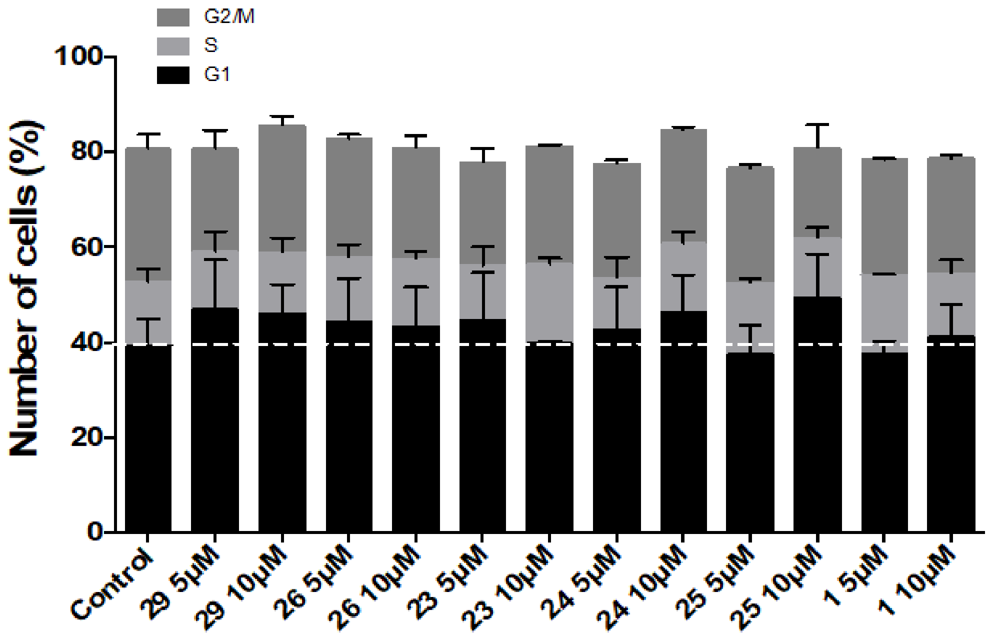

Cell cycle analysis of pancreatic cancer cells treated with the most active compounds and **1**. Aspc-1 (**A**) and PanAsc 2159 (**B**) cells were treated with the compounds at 5 and 10 µM for 48 hours. Results for the different stages of cell cycle are indicated for 3-4 independent experiments (mean ± SEM).

Kinase assay data

Entry	Kinase	% Activity ± SD (10 μM)	
		Compound **26**	Compound **23**
**1**	MKK1	108 ± 2	111 ± 20
**2**	MKK2	119 ± 5	100 ± 12
**3**	MKK6	119 ± 0	100 ± 12
**4**	ERK1	111± 8	98 ± 6
**5**	ERK2	103 ± 4	90 ± 2
**6**	ERK5	104 ± 5	111 ± 1
**7**	JNK1	110 ± 22	88 ± 9
**8**	JNK2	107 ± 1	100 ± 7
**9**	JNK3	104 ± 5	97 ± 1
**10**	p38a MAPK	102 ± 13	107 ± 1
**11**	p38b MAPK	84 ± 21	87 ± 17
**12**	p38g MAPK	132 ± 7	107 ± 18
**13**	p38d MAPK	107 ± 6	94 ± 1
**14**	ERK8	106 ± 2	105 ± 5
**15**	RSK1	84 ± 16	98 ± 5
**16**	RSK2	66 ± 15	65 ± 9
**17**	PDK1	100 ± 3	84 ± 13
**18**	PKBa	108 ± 20	107 ± 1
**19**	PKBb	90 ± 4	60 ± 2
**20**	SGK1	105 ± 16	87 ± 13
**21**	S6K1	119 ± 10	94 ± 9
**22**	PKA	101 ± 1	95 ± 5
**23**	ROCK 2	111 ± 13	95 ± 4
**24**	PRK2	123 ± 20	110 ± 13
**25**	PKCα	102 ± 5	101 ± 4
**26**	PKCγ	94 ± 3	98 ± 11
**27**	PKCζ	93 ± 7	98 ± 5
**28**	PKD1	125 ± 20	108 ± 1
**29**	STK33	121 ± 21	96 ± 14
**30**	MSK1	96 ± 12	97 ± 4
**31**	MNK1	98 ± 26	103 ± 0

**32**	MNK2	136 ± 4	130 ± 17
**33**	MAPKAP-K2	99 ± 3	106 ± 3
**34**	MAPKAP-K3	86 ± 19	99 ± 3
**35**	PRAK	108 ± 10	110 ± 1
**36**	CAMKKb	106 ± 9	88 ± 14
**37**	CAMK1	87 ± 4	78 ± 0
**38**	SmMLCK	103 ± 3	85 ± 1
**39**	PHK	102 ± 3	94 ± 4
**40**	DAPK1	104 ± 5	105 ± 0
**41**	CHK1	99 ± 7	87 ± 14
**42**	CHK2	122 ± 17	115 ± 4
**43**	GSK3b	92 ± 16	103 ± 8
**44**	CDK2-Cyclin A	111 ± 10	121 ± 29
**45**	CDK9-Cyclin T1	104 ± 6	109 ± 4
**46**	PLK1	92 ± 8	77 ± 3
**47**	Aurora A	101 ± 13	87 ± 1
**48**	Aurora B	93 ± 4	78 ± 0
**49**	TLK1	103 ± 1	96 ± 3
**50**	LKB1	110 ± 8	98 ± 2
**51**	AMPK (hum)	103 ± 2	96 ± 7
**52**	MARK1	105 ± 1	95 ± 3
**53**	MARK2	110 ± 1	85 ± 15
**54**	MARK3	102 ± 7	107 ± 14
**55**	MARK4	95 ± 1	92 ± 2
**56**	BRSK1	105 ± 1	89 ± 15
**57**	BRSK2	111 ± 1	97 ± 9
**58**	MELK	101 ± 6	96 ± 7
**59**	NUAK1	79 ± 10	96 ± 3
**60**	SIK2	100 ± 5	108 ± 2
**61**	SIK3	91 ± 4	97 ± 2
**62**	TSSK1	123 ± 3	101 ± 1
**63**	CK1γ2	90 ± 10	97 ± 4
**64**	CK1δ	106 ± 6	101 ± 1
**65**	CK2	104 ± 10	104 ± 4

**66**	TTBK1	104 ± 16	112 ± 2
**67**	TTBK2	117 ± 6	125 ± 1
**68**	DYRK1A	89 ± 3	109 ± 21
**69**	DYRK2	107 ± 2	115 ± 16
**70**	DYRK3	90 ± 6	92 ± 16
**71**	NEK2a	112 ± 11	110 ± 9
**72**	NEK6	97 ± 17	89 ± 2
**73**	IKKb	85 ± 10	70 ± 17
**74**	IKKe	101 ± 16	92 ± 6
**75**	TBK1	106 ± 3	92 ± 3
**76**	PIM1	89 ± 6	83 ± 1
**77**	PIM2	115 ± 15	109 ± 7
**78**	PIM3	94 ± 9	89 ± 6
**79**	SRPK1	116 ± 6	105 ± 8
**80**	EF2K	109 ± 4	102 ± 9
**81**	EIF2AK3	112 ± 9	102 ± 6
**82**	HIPK1	100 ± 3	100 ± 6
**83**	HIPK2	110 ± 21	114 ± 5
**84**	HIPK3	96 ± 8	91 ± 3
**85**	CLK2	100 ± 17	96 ± 2
**86**	PAK2	111 ± 16	105 ± 4
**87**	PAK4	96 ± 7	84 ± 25
**88**	PAK5	95 ± 16	105 ± 2
**89**	PAK6	102 ± 7	95 ± 4
**90**	MST2	86 ± 5	73 ± 11
**91**	MST3	125 ± 12	94 ± 4
**92**	MST4	116 ± 14	88 ± 1
**93**	GCK	110 ± 4	121 ± 3
**94**	MAP4K3	114 ± 17	123 ± 13
**95**	MAP4K5	98 ± 8	111 ± 6
**96**	MINK1	104 ± 7	97 ± 24
**97**	MEKK1	91 ± 6	89 ± 12
**98**	MLK1	101 ± 19	95 ± 1
**99**	MLK3	99 ± 1	92 ± 31

**100**	TESK1	103 ± 32	76 ± 21
**101**	TAO1	105 ± 4	93 ± 2
**102**	ASK1	103 ± 5	98 ± 2
**103**	TAK1	112 ± 2	103 ± 3
**104**	IRAK1	117 ± 4	105 ± 11
**105**	IRAK4	92 ± 14	91 ± 8
**106**	RIPK2	116 ± 9	117 ± 39
**107**	OSR1	107 ± 17	111 ± 3
**108**	TTK	90 ± 3	82 ± 9
**109**	MPSK1	100 ± 6	99 ± 2
**110**	WNK1	103 ± 4	95 ± 7
**111**	ULK1	103 ± 7	98 ± 2
**112**	ULK2	104 ± 12	97 ± 5
**113**	TGFBR1	97 ± 9	97 ± 20
**114**	Src	90 ± 2	72 ± 6
**115**	Lck	95 ± 15	68 ± 7
**116**	CSK	92 ± 3	106 ± 4
**117**	YES1	90 ± 5	95 ± 11
**118**	ABL	96 ± 6	101 ± 2
**119**	BTK	87 ± 1	71 ± 0
**120**	JAK3	90 ± 3	95 ± 15
**121**	SYK	92 ± 11	90 ± 1
**122**	ZAP70	91 ±15	102 ± 22
**123**	TIE2	95 ± 14	104 ± 22
**124**	BRK	89 ± 6	93 ± 18
**125**	EPH-A2	116 ± 55	94 ± 19
**126**	EPH-A4	124 ± 9	104 ± 55
**127**	EPH-B1	111 ± 33	110 ± 9
**128**	EPH-B2	106 ± 23	110 ± 16
**129**	EPH-B3	119 ± 7	115 ± 9
**130**	EPH-B4	111 ± 13	111 ± 12
**131**	FGF-R1	102 ± 31	88 ± 20
**132**	HER4	94 ± 7	88 ± 26
**133**	IGF-1R	109 ± 4	122 ± 10

**134**	IR	97 ± 11	93 ± 6
**135**	IRR	104 ± 3	85 ± 3
**136**	TrkA	95 ± 12	99 ± 14
**137**	DDR2	132 ± 38	97 ± 12
**138**	VEG-FR	96 ± 2	90 ± 7
**139**	PDGFRA	82 ± 4	82 ± 7
**140**	PINK	117 ± 5	103 ± 7

Table S1. Data from the kinase screening for compounds **23** and **26**.

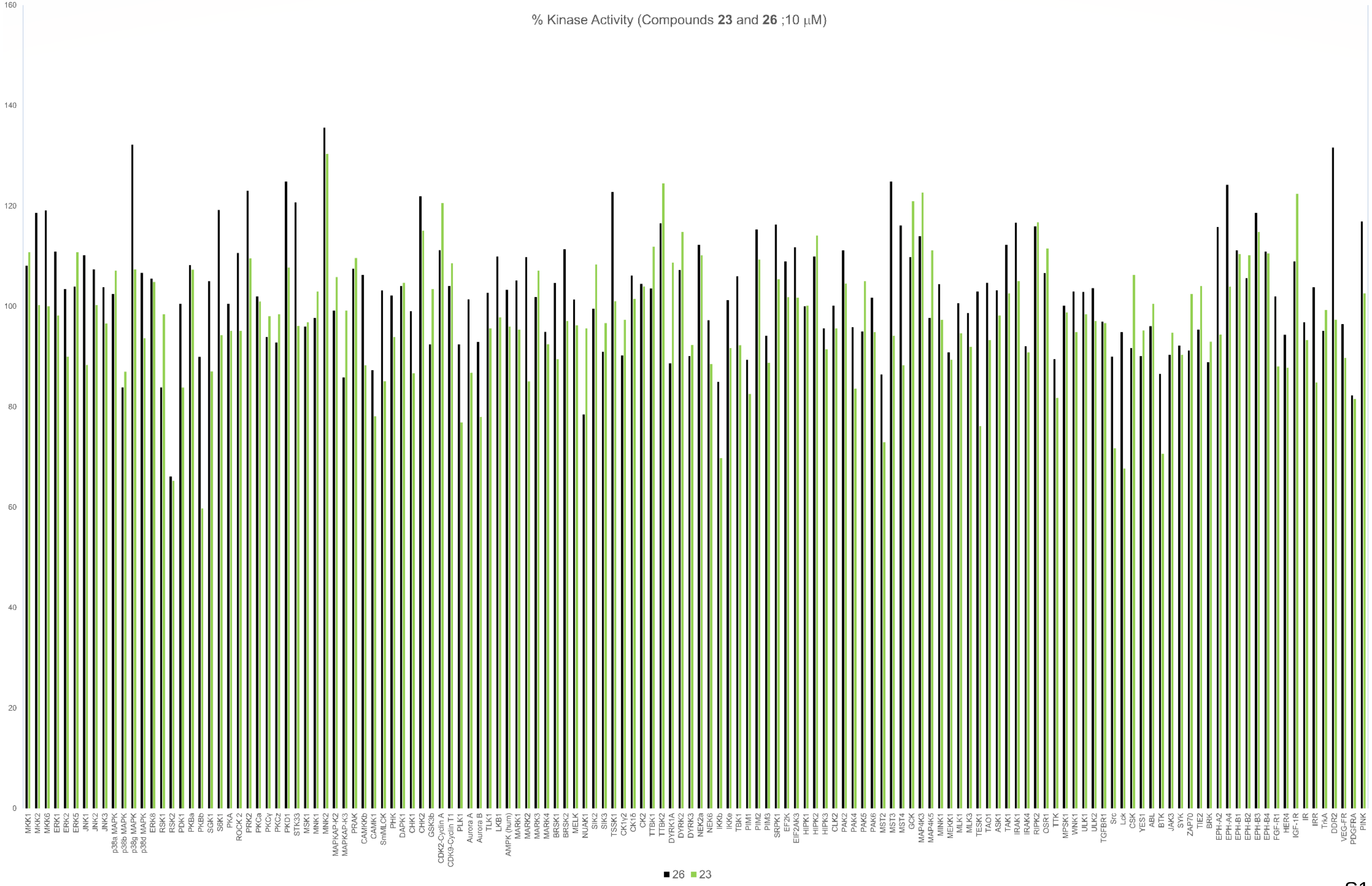
% Kinase Activity (Compounds 23 and 26 ;10 μM)
160
140
120
100
80
60
40
20
0
MKK1
MKK2
MKK6
ERK1
ERK2
ERK5
JNK1
JNK2
JNK3
p38a MAPK
p38b MAPK
p38g MAPK
p38d MAPK
ERK8
RSK1
RSK2
PDK1
PKBa
PKBb
SGK1
S6K1
PKA
ROCK 2
PRK2
PKCa
PKCγ
PKCz
PKD1
STK33
MSK1
MNK1
MNK2
MAPKAP-K2
MAPKAP-K3
PRAK
CAMKKb
CAMK1
SmMLCK
PHK
DAPK1
CHK1
CHK2
GSK3b
CDK2-Cyclin A
CDK9-Cyclin T1
PLK1
Aurora A
Aurora B
TLK1
LKB1
AMPK (hum)
MARK1
MARK2
MARK3
MARK4
BRSK1
BRSK2
MELK
NUAK1
SIK2
SIK3
TSSK1
CK1γ2
CK1δ
CK2
TTBK1
TTBK2
DYRK1A
DYRK2
DYRK3
NEK2a
NEK6
IKKb
IKKe
TBK1
PIM1
PIM2
PIM3
SRPK1
EF2K
EIF2AK3
HIPK1
HIPK2
HIPK3
CLK2
PAK2
PAK4
PAK5
PAK6
MST2
MST3
MST4
GCK
MAP4K3
MAP4K5
MINK1
MEKK1
MLK1
MLK3
TESK1
TAO1
ASK1
TAK1
IRAK1
IRAK4
RIPK2
OSR1
TTK
MPSK1
WNK1
ULK1
ULK2
TGFBR1
Src
Lck
CSK
YES1
ABL
BTK
JAK3
SYK
ZAP70
TIE2
BRK
EPH-A2
EPH-A4
EPH-B1
EPH-B2
EPH-B3
EPH-B4
FGF-R1
HER4
IGF-1R
IR
IRR
TrkA
DDR2
VEG-FR
PDGFRA
PINK
26
23
